# Asymmetric counteranion-directed Lewis acid organocatalysis for the scalable cyanosilylation of aldehydes

**DOI:** 10.1038/ncomms12478

**Published:** 2016-08-17

**Authors:** Zhipeng Zhang, Han Yong Bae, Joyram Guin, Constantinos Rabalakos, Manuel van Gemmeren, Markus Leutzsch, Martin Klussmann, Benjamin List

**Affiliations:** 1 Max-Planck-Institut für Kohlenforschung, Kaiser-Wilhelm-Platz 1, Mülheim an der Ruhr 45470, Germany

## Abstract

Due to the high versatility of chiral cyanohydrins, the catalytic asymmetric cyanation reaction of carbonyl compounds has attracted widespread interest. However, efficient protocols that function at a preparative scale with low catalyst loading are still rare. Here, asymmetric counteranion-directed Lewis acid organocatalysis proves to be remarkably successful in addressing this problem and enabled a molar-scale cyanosilylation in quantitative yield and with excellent enantioselectivity. Also, the catalyst loading could be lowered to a part-per-million level (50 ppm: 0.005 mol%). A readily accessible chiral disulfonimide was used, which in combination with trimethylsilyl cyanide, turned into the active silylium Lewis acid organocatalyst. The nature of a peculiar phenomenon referred to as a “dormant period”, which is mainly induced by water, was systematically investigated by means of *in situ* Fourier transform infrared analysis.

## Abstract (web summary)

Asymmetric cyanosilyation is a powerful method to convert carbonyls to chiral, configurationally stable cyanohydrins. Here, the authors report a catalyst capable of carrying out this reaction on large scales with extremely low catalyst loading, and also identify and explain a dormant period in the cycle.

## Graphical abstract

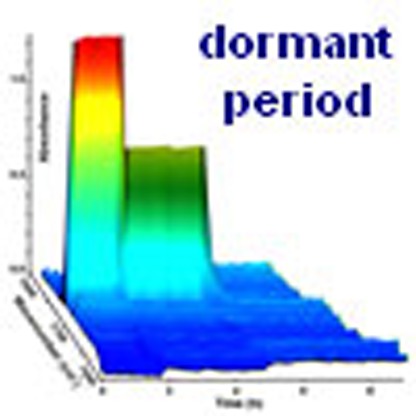

As a remarkably efficient approach to obtain enantioenriched organic compounds, asymmetric catalysis has attracted significant attention^1^,^2^. In particular, asymmetric organocatalysis, which is an effective complement to well-established methods such as biocatalysis and transition metal catalysis, has emerged as a powerful tool^3^,^4^,^5^,^6^,^7^. While metal-based Lewis acids have been intensively studied in asymmetric catalysis^8^,^9^, the complementary metal-free Lewis acid organocatalysts have been rarely explored. Our research group has recently developed chiral disulfonimides as efficient Brønsted acid organocatalysts^10^,^11^, which can conveniently be utilized as precursors for silylium-mediated Lewis acid organocatalysts, when combined with silicon containing organic nucleophiles^11^,^12^,^13^. This catalytic system afforded remarkably high enantioselection, by pairing a silylium ion equivalent with a chiral enantiopure counteranion, exploiting the concept of asymmetric counteranion-directed catalysis^14^. This strategy recently led to a number of applications involving several different silicon-containing nucleophiles, for example, silyl ketene acetals^13^,^15^,^16^, Danishefsky-type dienes^17^, allylsilanes^18^,^19^ and silyl phosphites^20^.

The catalytic asymmetric cyanation of carbonyl compounds (for example, aldehydes and ketones) is an attractive protocol to obtain chiral cyanohydrin derivatives^21^. This methodology offers a convenient access to a variety of valuable chiral building blocks, as well as biologically active compounds, such as *α*-hydroxy acids, *β*-amino alcohols and *α*-amino acids^22^,^23^,^24^,^25^. After the initial report on the enantioselective addition of hydrogen cyanide to benzaldehyde in the presence of an extract of almonds as catalyst^26^, numerous enzymatic^27^,^28^ and metal-based Lewis acid catalytic systems^29^,^30^,^31^,^32^,^33^,^34^,^35^,^36^ have been investigated for the synthesis of enantioenriched cyanohydrins. A few efficient metal-free, hydrogen bonding or boron promoted protocols have also been reported.^37^,^38^,^39^,^40^,^41^,^42^,^43^ However, the use of asymmetric counteranion-directed catalysis towards this goal remained unprecedented.

Herein we disclose an asymmetric Lewis acid organocatalysis approach for the cyanosilylation of wide range of aldehydes (**2**) with trimethylsilyl cyanide (TMSCN, **3**), which utilizes a chiral disulfonimide (**1**) as pre-catalyst (Fig. 1). The developed catalytic system proved to be highly active and scalable, enabling a cyanohydrin synthesis at a scale of greater than 150 g in the presence of merely 0.05 mol% of the catalyst. Moreover, the catalyst loading could be reduced to as little as 0.005 mol% (50 ppm). Furthermore, we observed an interesting phenomenon referred to as dormant period, during which the catalyst is completely inactive and which is reversibly induced by water. This phenomenon was systematically investigated by means of *in situ* Fourier transform infrared (FT-IR) analysis, providing important mechanistic insight into the pre-catalytic cycle.

## Results

### Optimization of the reaction conditions

**Figure 2:**
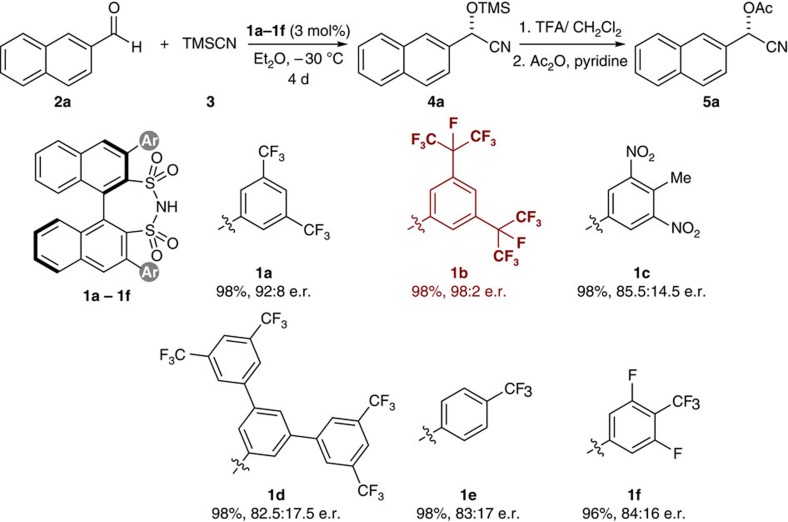
Influence of the catalyst structure on the reactivity and enantioselectivity. Conditions: aldehyde **2a** (0.05 mmol), TMSCN **3** (0.1 mmol) and catalyst **1** (3 mol%) in Et_2_O (0.16 M) at –30 °C for 4 days unless noted otherwise. Isolated yields were determined after converting to the corresponding acetate **5a**. The e.r. of acetates **5a** was determined by HPLC on a chiral stationary phase.

We commenced our study by carrying out the asymmetric cyanosilylation of 2-naphthaldehyde (**2a**) with TMSCN (**3**) as a model reaction to afford chiral cyanohydrin **4a**. After screening a wide variety of solvents in the presence of disulfonimide catalyst **1a** (3 mol%), diethyl ether (Et_2_O) was identified as the most efficient reaction medium in terms of enantioselectivity (Supplementary Table 1). Next, several chiral disulfonimide catalysts **1a**–**1f** were screened to elucidate the relationship between the catalyst's structure and the reaction outcome (Fig. 2). The performance of screened catalysts, regarding enantioselectivity was highly dependent on the steric demand of the aryl substituents. To our delight, disulfonimide catalyst **1b,** which incorporated a larger perfluoroisopropyl substituent gave an excellent enantiomeric ratio of 98:2 e.r.

### Substrate scope of the reaction

**Figure 3:**
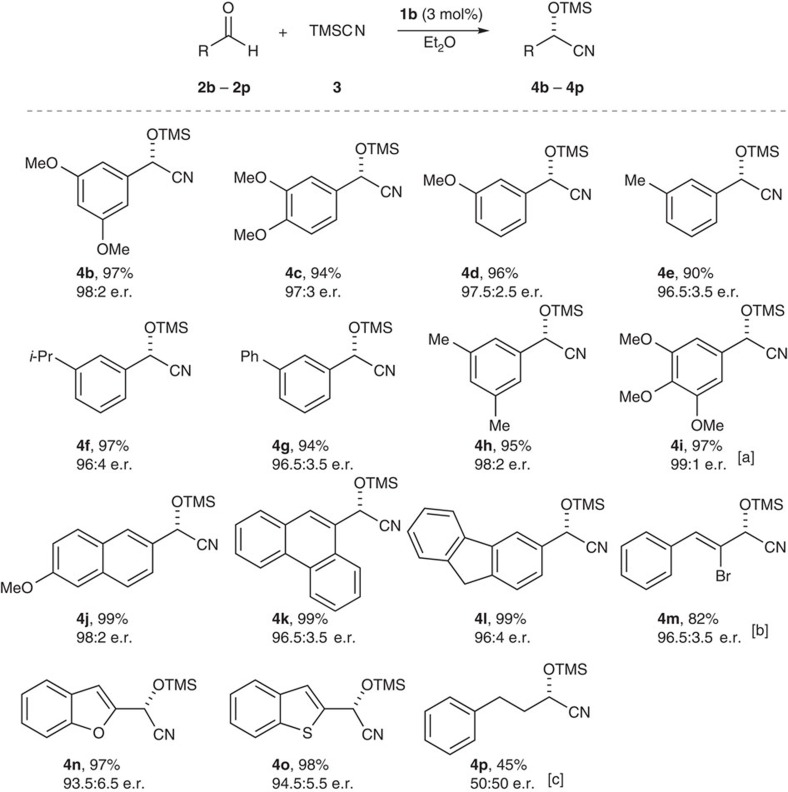
Scope of the asymmetric cyanosilylation of aldehydes (2b-2p) catalysed by disulfonimide 1b. Conditions: aldehyde **2** (0.05 mmol), TMSCN (0.1 mmol) and catalyst **1b** (3 mol%) in Et_2_O (0.16 M) at –30 °C for 4 days unless noted otherwise. Isolated yields were determined after converting to the corresponding acetate **5**. The e.r. of acetates **5** was determined by HPLC on a chiral stationary phase. [a] –50 °C, 7 days. [b] 7 days. [c] –10 °C, 7 days.

Under optimized reaction conditions, a variety of aldehydes **2** were subjected to this protocol. As shown in Fig. 3, several aryl aldehydes **2b**–**2l** were smoothly converted into the corresponding chiral cyanohydrins **4b**–**4l** with excellent to quantitative yields (90-99%) and excellent enantiomeric ratios (96:4 to 99:1). An alkenyl substrate, α-bromocinnamaldehyde **2m**, was also smoothly converted to the desired product **4m** in good yield with excellent enantiomeric ratio (82%, 96.5:3.5 e.r.). Furthermore, heteroaromatic aldehydes **2n** and **2o** also successfully afforded enantioenriched products **4n** and **4o** (97 and 98%, 93.5:6.5 e.r. and 94.5:5.5 e.r. respectively). The primary alkyl substrate hydrocinnamaldehyde (**2p**) gave product **4p** with moderate yield (45%) and essentially no enantioselection.

### Scale-up syntheses and part-per-million level catalysis

**Figure 4:**
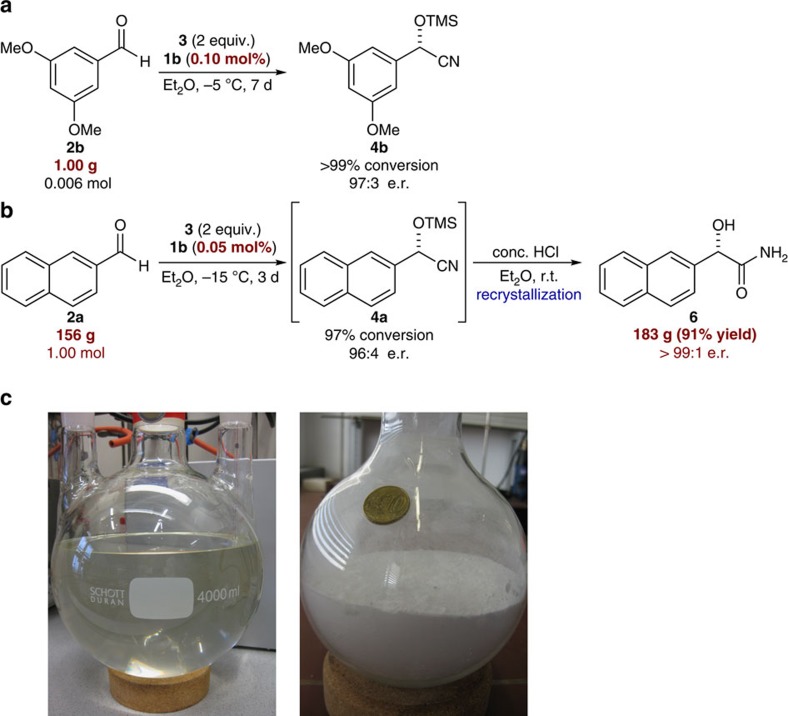
Large-scale syntheses of catalytic cyanosilylation. (**a**) 1.00 g scale synthesis of cyanohydrin **4b**. (**b**) 156 g scale synthesis of acetamide **6**. (**c**) Reaction mixture of the cyanosilylation of aldehyde **2a** (156 g) towards **4a** after 3 days (left) and the obtained acetamide **6** (91% yield, 183 g, right).

To demonstrate the practicability of the developed protocol, preparative scale syntheses were conducted. At the beginning, a cyanosilylation with 1.00 g scale (0.006 mol) of aldehyde **2b** in the presence of 0.10 mol% of disulfonimide catalyst **1b** was carried out (Fig. 4a). The reaction proceeded smoothly and gave the desired product **4b** in >99% conversion (^1^H NMR) and with an excellent enantiomeric ratio (97:3 e.r.). Next, a significantly increased scale was chosen and the catalytic cyanosilylation starting with 156 g (1.00 mol) of aldehyde **2a** was conducted (Fig. 4b). It is noteworthy that a catalyst loading of merely 0.05 mol% was sufficient to achieve almost full conversion with excellent enantioselection (Fig. 4c left, 97% ^1^H NMR conversion, 96:4 e.r.) The obtained cyanohydrin **4a** was directly hydrolysed under acidic conditions and a subsequent recrystallization provided 183 g (91% yield) of enantiomerically pure (>99:1 e.r.) (*S*)-2-hydroxy-2-(naphthalen-2-yl)acetamide **6** (Fig. 4c right). In addition, ca. 80% of catalyst **1b** was recovered by chromatographic purification in high purity (see Supplementary Methods for details).

An even further reduced catalyst loading of only 0.005 mol% (50 ppm) of catalyst **1b** could be successfully utilized to catalyse the reaction of aldehyde **2b** to give cyanohydrin **4b**, in excellent conversion (92%, turnover number=18,400) and with 88.5:11.5 e.r. Enantioselective organocatalytic C-C bond forming reactions with such low catalyst loadings have been extremely rare^44^ (see Supplementary Methods for details).

### *In situ* FT-IR study on the dormant period

Despite the fact that in most cases full conversions with excellent enantioselectivities were achieved using our disulfonimide catalyst, rather long reaction times (3–7 days) were usually required. While investigating this particular issue, an interesting phenomenon was observed. When the reaction progress was monitored in the early stage of the reaction using thin layer chromatography and ^1^H NMR spectroscopy, absolutely no conversion to product was detected. However, at some point in time, the reaction suddenly proceeded very rapidly and was soon found to be completed. In order to gain insight into the details of this unusual phenomenon, we decided to follow the reaction progress by means of *in situ* FT-IR spectroscopic analysis. This method was chosen, as it allows investigating the actual course of the reaction in real time, without requiring taking aliquots from the reaction mixture. The stretching vibration of the carbonyl group of an aldehyde gives a strong and distinctive infrared band, which is well suited for the *in situ* FT-IR measurement. Commercially available disulfonimide catalyst **1a**^12^ was selected for this mechanistic study.

**Figure 5:**
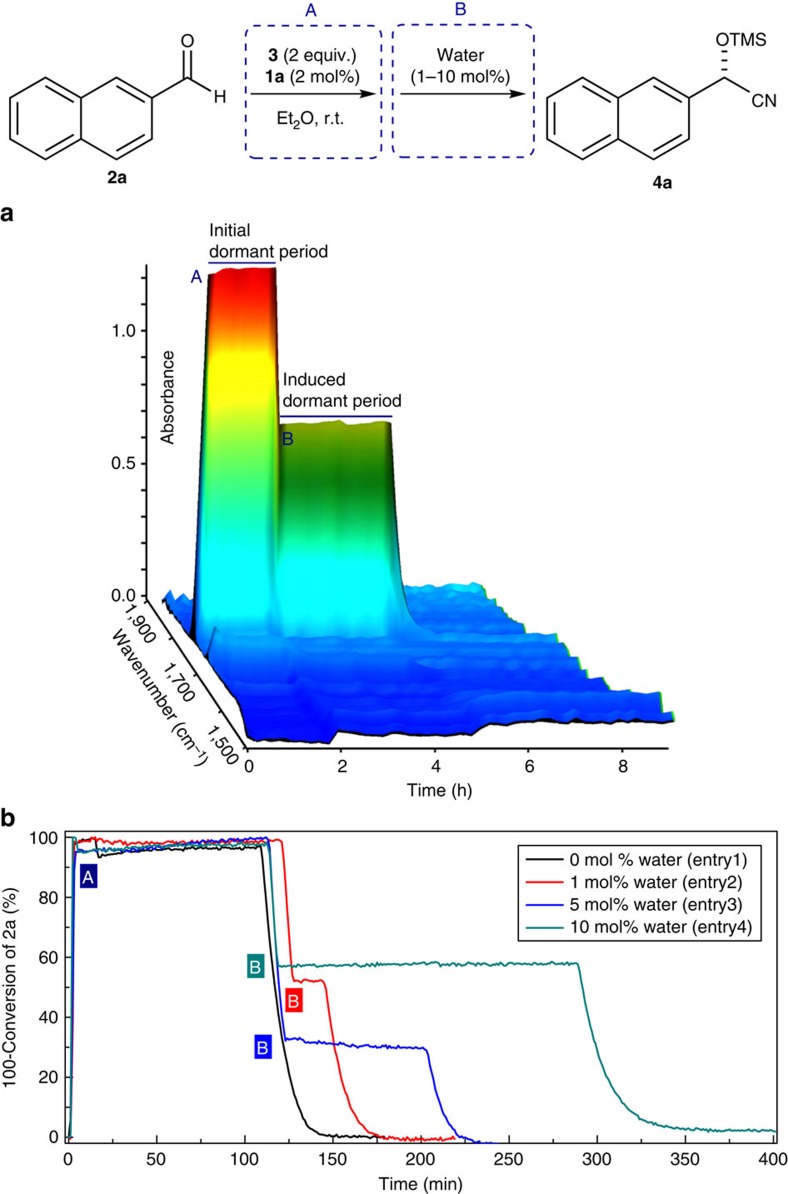
Study on the dormant period monitored by means of *in situ* FT-IR analysis. Conditions of step A: aldehyde **2a** (0.10 mmol), TMSCN **3** (2.0 equiv.) and catalyst **1a** (2 mol%) in Et_2_O (5.0 ml) at 25 °C. Water (1–10 mol%) was added at the marked time point (step B). (**a**) Three-dimensional stack plot of the IR spectra (10 mol% water). (**b**) Plot of conversion of **2a** versus time (1–10 mol% water). See Table 1 for the length of dormant periods.

As displayed in Fig. 5, the absorption peak of the carbonyl group of aldehyde **2a** at 1703, cm^−1^ was monitored during the progress of the catalytic cyanosilylation and an interesting trend was observed. After dissolving aldehyde **2a**, catalyst **1a** and TMSCN (**3**) in Et_2_O, no conversion was detected in the first 110 min. Surprisingly, after this initial dormant period, the reaction abruptly proceeded and reached >99% conversion (by ^1^H NMR) within 30 min (Table 1, entry 1). We hypothesized that this reluctance in the early stage was derived mainly from water present in the reaction mixture and thus decided to conduct further control experiments by deliberately adding varied amounts of water. After the initiation of the reaction (step A) and 118 min of the initial dormant period, which results from humidity that is not deliberately added, 1 mol% of water (0.2 μl) was added (step B) when the amount of remaining aldehyde **2a** had reached 52%. Here, consistent with our expectation, the reaction was immediately suspended and an induced dormant period was observed, which lasted for 17 min. After this period, the reaction restarted automatically and aldehyde **2a** was gradually consumed within the next 20 min (Table 1, entry 2). Using a higher loading of water led to an approximately linear correlation with the length of the induced dormant period. Accordingly, induced dormant periods of 80 and 170 min respectively were observed, when 5 mol% (0.9 μl) and 10 mol% (1.8 μl) of water were added (Table 1, entries 3 and 4). These control experiments clearly indicate that water can induce the dormant period, presumably by hydrolysing the catalytically active species (**1a**-**TMS**), which prevents efficient catalysis (see Supplementary Figs. 1–4 for details).

**Figure 6:**
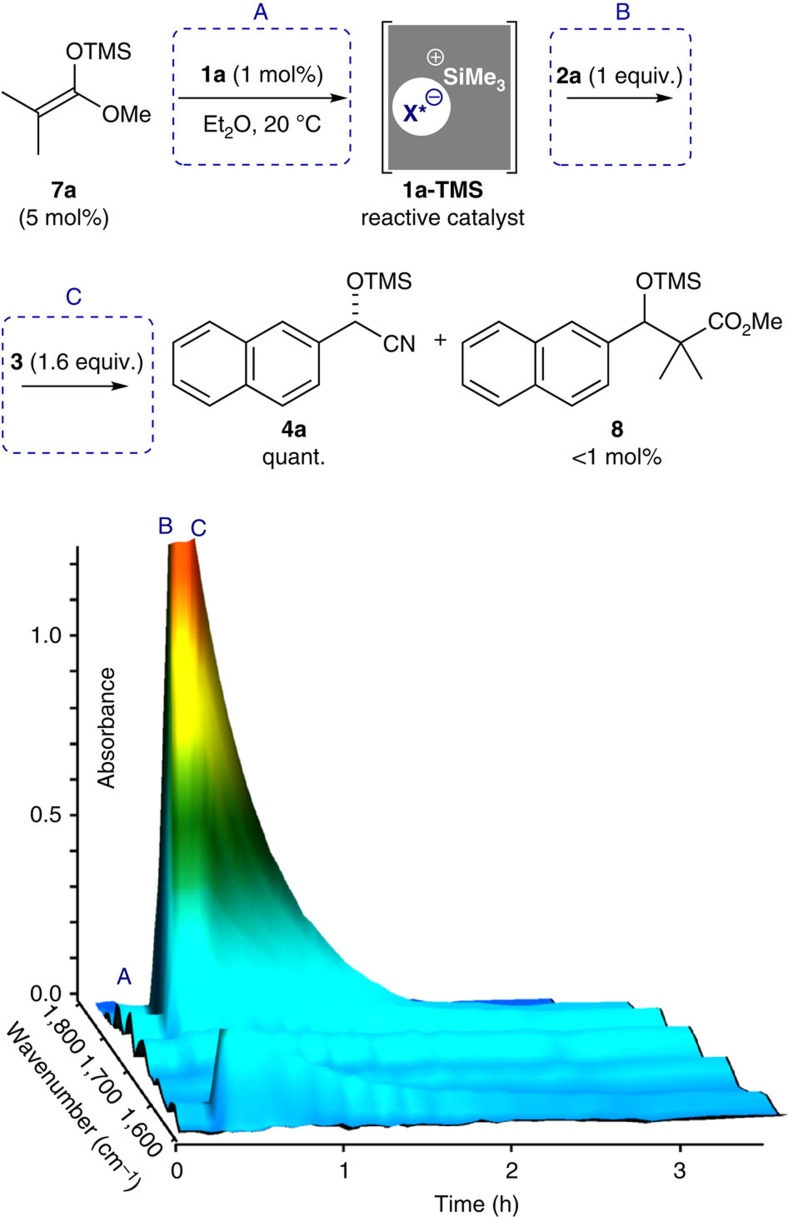
Catalytic cyanosilylation using *in situ* generated reactive catalyst 1a-TMS. Reaction conditions: **1a** (0.01 mmol, 1 mol%), 20 °C, Et_2_O (5.0 ml), **7a** (0.05 mmol, 5 mol%), **2a** (1.0 mmol, 0.20 M), **3** (1.6 mmol, 0.32 M). The reaction was monitored by means of *in situ* FT-IR analysis.

In contrast to the above findings, in our previous *in situ* FT-IR study on the disulfonimide **1a** catalysed Mukaiyama aldol reaction of silyl ketene acetal with aldehyde **2h**, the reaction started immediately and no dormant period was observed^45^. This might be due to the high reactivity of the silyl ketene acetal with the pre-catalyst **1a**, which instantaneously (re-)generates the active Lewis acid organocatalyst. Based on this knowledge, we hypothesized that the utilization of a catalytic amount of silyl ketene acetal as an activator, such as for example methyl trimethylsilyl dimethylketene acetal **7a**, might shorten or avoid the dormant period. In this context, we implemented an experiment regarding pre-activation of **1a** to **1a**-**TMS** by the treatment of 5 mol% of silyl ketene acetal **7a** (step A), then successive addition of aldehyde **2a** (step B) and TMSCN **3** (step C) as depicted in Fig. 6. To our delight, the dormant period was avoided. The reaction started immediately and reached full conversion to product **4a** within less than 1 h with formation of only 0.9 mol% of the Mukaiyama aldol product **8** (see Supplementary Fig. 5 for details).

**Figure 7:**
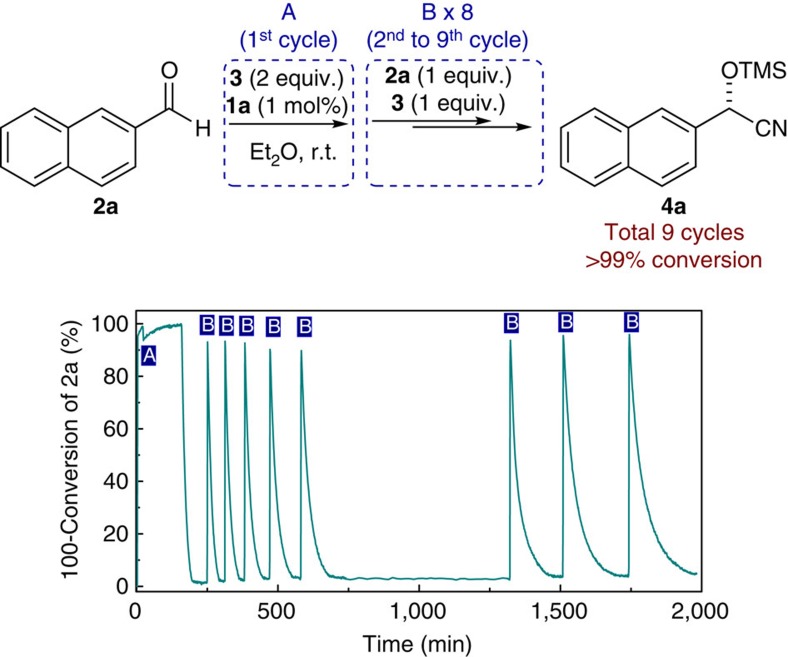
Experiment on repetitive substrate addition cycles. Reaction conditions (first cycle): **1a** (0.01 mmol, 1 mol%), 25 °C, Et_2_O (4.6 ml), **2a** (1.0 mmol, 1.0 equiv.), **3** (2.0 mmol, 2.0 equiv.). Reaction conditions (second to ninth cycle): addition of **2a** (1.0 mmol, 1.0 equiv.), **3** (2.0 mmol, 2.0 equiv.). The reaction was monitored by means of *in situ* FT-IR analysis.

Importantly, after completion of the reaction, the generated active catalyst **1a**-**TMS** remained stable in the absence of humidity and its repeated utilization could be successfully realized. As shown in Fig. 7, one equivalent of each compound **2a** and TMSCN (**3**) was added to the reaction mixture (step B), after the first catalytic reaction (initiated by step A) was completed. Eight further cycles of substrate addition were conducted and the completion of each independent cycle was monitored by *in situ* FT-IR. Here, no further dormant periods were observed and full conversion to the desired cyanohydrin **2a** occurred in each of the overall nine cycles (see Supplementary Fig. 6 for details).

## Discussion

**Figure 8:**
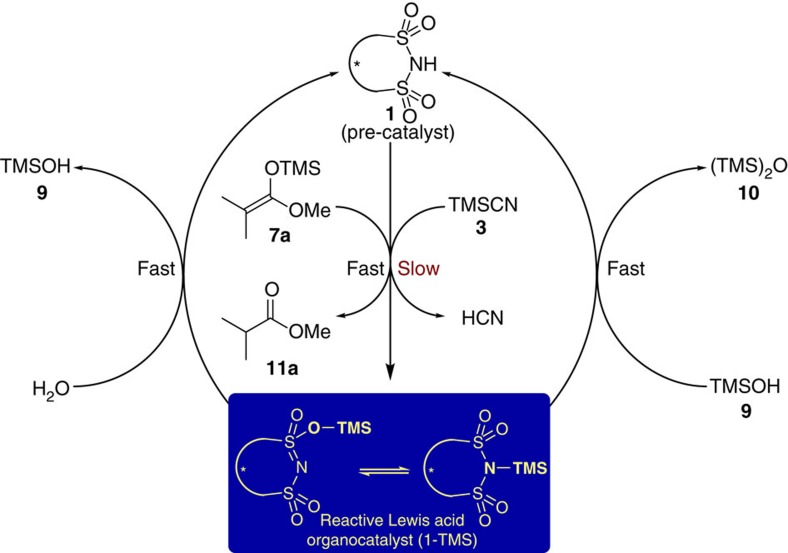
Proposed pre-catalytic cycle (dormant period). The reactive Lewis acid organocatalyst (**1-TMS**) is formed in the presence of TMSCN (**3**) or silyl ketene acetal (**7a**) and quenched in the presence of H_2_O or silanol **9**.

During the pre-catalytic cycle (dormant period), Brønsted acid **1** slowly reacts with TMSCN (**3**) to generate the active Lewis acid organocatalyst **1**-**TMS** and HCN. The resulting species **1**-**TMS** is immediately hydrolysed back to the pre-catalyst **1**, while water and silanol **9** are being converted to silanol **9** and hexamethyldisiloxane **10**, respectively. After water and silanol **9** are completely consumed, the silylated species **1**-**TMS** starts to catalyse the addition of TMSCN (**3**) to aldehyde **2**. Further studies revealed that the addition of trimethylsilanol **9** during the course of the reaction also induces a dormant period, while hexamethyldisiloxane **10** has no influence on the reaction progress (see Supplementary Fig. 7). ^1^H-^29^Si-HMBC and 2D-EXSY NMR studies revealed that **1a**-**TMS** exists as a mixture of *O*- and *N*-silylated tautomers (see Supplementary Figs. 8, 9 and Supplementary Table 2). The build-up and decay of the silylated species **1a-TMS** were also studied by ^1^H NMR and the results are in accordance with the *in situ* FT-IR studies regarding the dormant period, which is induced by water addition (see Supplementary Fig. 10). Based on these results, we propose a pre-catalytic cycle (dormant period) as shown in Fig. 8.

In conclusion, we have successfully developed an asymmetric counteranion-directed cyanosilylation promoted by a silylium Lewis acid organocatalyst. In the presence of the highly accessible disulfonimide catalyst **1**, a variety of aldehydes were converted into the corresponding cyanohydrins in excellent yields and enantiomeric ratios. The developed protocol is scalable to more than 150 g of substrate. The catalyst loading could be reduced to as little as 0.005 mol% (50 ppm). The reaction is triggered through the formation of active catalyst **1**-**TMS** via a dormant period causing pre-catalytic cycle (see Supplementary Discussion concerning induction period versus dormant period). This phenomenon was shown to be predominantly induced by water, and was systematically studied by means of *in situ* FT-IR analysis.

## Methods

### Procedure for the asymmetric cyanosilylation reactions

A dried vial with a Teflon-coated magnetic stirring bar was charged with disulfonimide **1** (0.0015, mmol, 3 mol%), aldehyde **2** (0.05 mmol, 1.0 equiv.) and Et_2_O (0.31 ml). TMSCN **3** (0.1 mmol, 2.0 equiv.) was added to the reaction mixture via a microlitre syringe and the vial was cooled to –30 °C immediately. The reaction mixture was stirred at –30 °C and the progress of the reaction was monitored by TLC. The reaction was quenched with 2% (v/v) trifluoroacetic acid in CH_2_Cl_2_ (0.38 ml) and water (2 μl) after the progress of the reaction was determined to be complete. The reaction mixture was warmed up to room temperature and stirred for 2 h to hydrolyse the TMS-protected cyanohydrin. The solvent and the volatile compounds were evaporated under reduced pressure at ambient temperature. Then CH_2_Cl_2_ (1 ml), acetic anhydride (47 μl, 10 equiv.) and pyridine (36 μl, 9 equiv.) were added and the reaction mixture was stirred at room temperature overnight. The solvent and volatile compounds were evaporated under reduced pressure at 40 °C. The residue was purified by column chromatography on silica gel using *iso*-hexane/ethyl acetate (4:1) as the eluent thus delivering the pure cyanohydrin acetate. The e.r. was determined by HPLC analysis of the cyanohydrin acetate on a chiral stationary phase.

### Procedure for the *in situ* study monitored by *in situ* FT-IR

Disulfonimide **1a** (8.2 mg, 0.010 mmol) and Et_2_O (4.54 ml) were placed in a pre-dried newly designed reactor (see Supplementary Fig. 11) equipped with a Teflon-coated magnetic stirring bar and thermometer under argon. A probe rod of ReactIR 15 (Mettler Toledo) was inserted into the solution, the reactor was connected with a high performance cryostat and the temperature was set to be 20 °C. The scanning (interval: 1 min) was started and 2-naphthaldehyde **2a** (156.2 mg, 1.000 mmol) was added to the mixture after a few minutes. The stretching vibration absorption of the carbonyl group (1703, cm^−1^) in **2a** was monitored. TMSCN (250 μl, 2.00 mmol) was added to the reaction mixture after 5 min and the reaction profile (IR versus time) was recorded. The reaction was quenched with water (10 μl) after most of the aldehyde **2a** had been converted to the product. The conversion of **2a** was determined by ^1^H NMR.

Other detailed experimental procedures and characterization data for all the new compounds are given in Supplementary Methods. For NMR and HPLC analysis of the compounds in this article, see Supplementary Figs. 12–45.

### Data availability

The authors declare that the data supporting the findings of this study are available within the article and its Supplementary Information files.

## Additional information

**How to cite this article**: Zhang Z. *et al.* Asymmetric counteranion-directed Lewis acid organocatalysis for the scalable cyanosilylation of aldehydes. *Nat. Commun.* 7:12478 doi: 10.1038/ncomms12478 (2016).

## Supplementary Material

Supplementary Information Supplementary Figures 1-45, Supplementary Tables 1-2, Supplementary Discussion, Supplementary Methods and Supplementary References

## Figures and Tables

**Figure 1:**
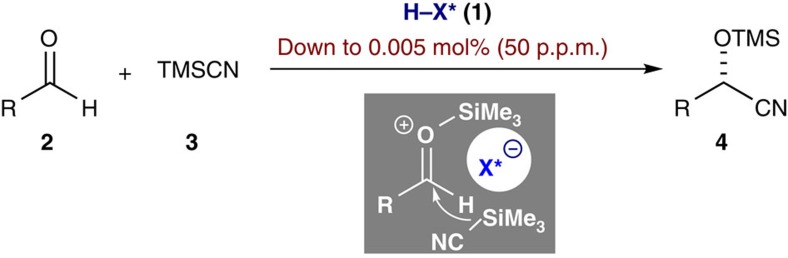
ACDC strategy for enantioselective cyanosilylation of aldehydes (2) with TMSCN (3). Chiral disulfonimide (**1**) was utilized as pre-catalyst and the loading could be reduced to as little as 0.005 mol%. ACDC, asymmetric counteranion-directed catalysis.

**Table 1:** Study on the dormant period monitored by means of *in situ* FT-IR analysis.

Entry	Initial dormant period (min)	Water added (mol%)	Induced dormant period (min)
1	110	0	0
2	118	1	17
3	109	5	80
4	109	10	170

Conditions of step A (Fig. 5): aldehyde **2a** (0.10 mmol), TMSCN **3** (2.0 equiv.) and catalyst **1a** (2 mol%) in Et_2_O (5.0 ml) at 25 °C. Water (1–10 mol%) was added at the marked time point step B (Fig. 5).
