# Supplementary material for: Asymmetric counteranion-directed Lewis acid organocatalysis for the scalable cyanosilylation of aldehydes
Source: Nat Commun. 2016 Aug 17;7:12478. doi: 10.1038/ncomms12478 (PMC4992067; doi:10.1038/ncomms12478)
Supplement: Supplementary Information — Supplementary Figures 1-45, Supplementary Tables 1-2, Supplementary Discussion, Supplementary Methods and Supplementary References [file ncomms12478-s1.pdf]

Supplementary Figures

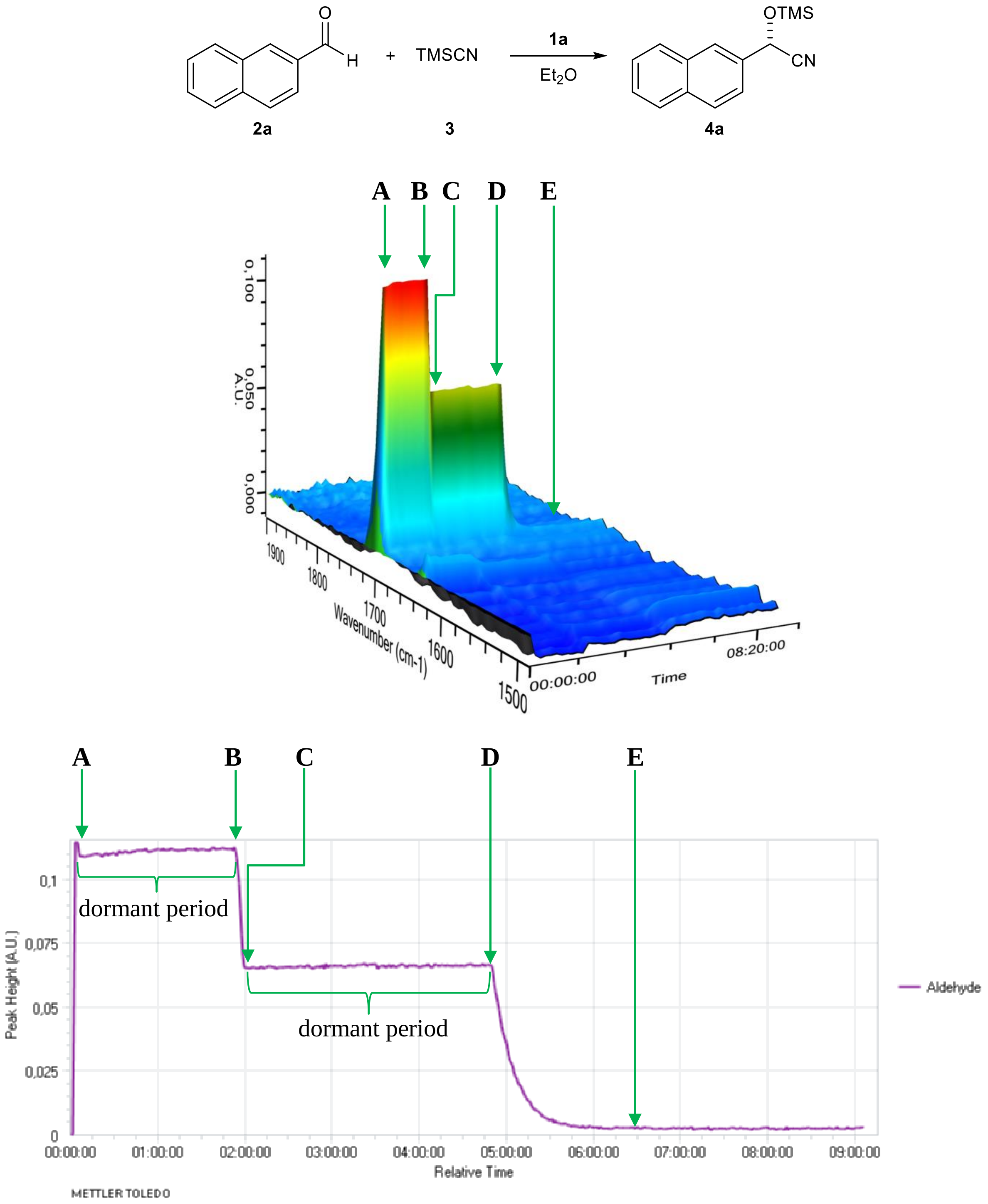

Supplementary Figure 1. The Influence of H_2O on the Dormant Period. The stretching vibration of the carbonyl group in **2a** (1703 cm^{-1}) was monitored by *in situ* FT-IR (**10 mol%** of water addition). Reaction conditions: **1a** (0.01 mmol, 2 mol%), **2a** (0.50 mmol, 0.20 M), **3** (1.0 mmol, 0.40 M), 25 ℃, Et_2O (2.5 mL). **A: 1a**, **2a**, Et_2O and **3** were added. **B:** Reaction started after dormant period. **C:** 10 mol% (0.9 μL) of H_2O were added at 40% conversion; reaction stopped. **D:** Reaction restarted after approximately 3 hours. **E:** Reaction completed.

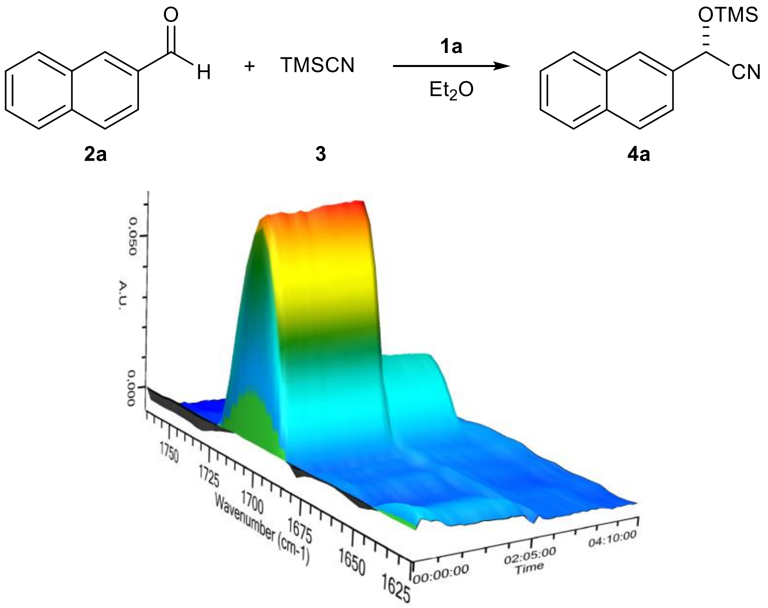

Supplementary Figure 2. The Influence of H_2O on the Dormant Period. The stretching vibration of the carbonyl group in **2a** (1703 cm^{-1}) was monitored by *in situ* FT-IR (**5 mol%** of water addition). Reaction conditions: **1a** (0.02 mmol, 2 mol%), **2a** (1.00 mmol, 0.20 M), **3** (2.00 mmol, 0.40 M), 25 °C, Et_2O (5.0 mL). 5 mol% of H_2O were added at about 70% conversion; reaction stopped. The reaction started again after 80 minutes.

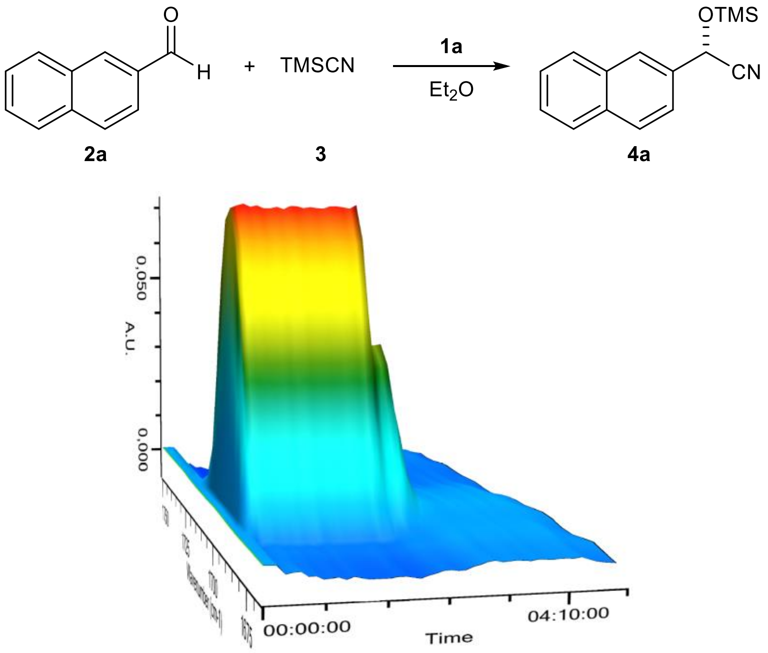

Supplementary Figure 3. The Influence of H_2O on the Dormant Period. The stretching vibration of the carbonyl group in **2a** (1703 cm^{-1}) was monitored by *in situ* FT-IR (**1 mol%** of water addition). Reaction conditions: **1a** (0.02 mmol, 2 mol%), **2a** (1.00 mmol, 0.20 M), **3** (2.00 mmol, 0.40 M), 25 °C, Et_2O (5.0 mL). 1 mol% of H_2O were added at about 50% conversion; reaction stopped. The reaction started again after 17 minutes.

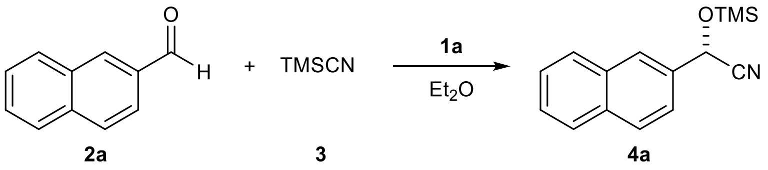

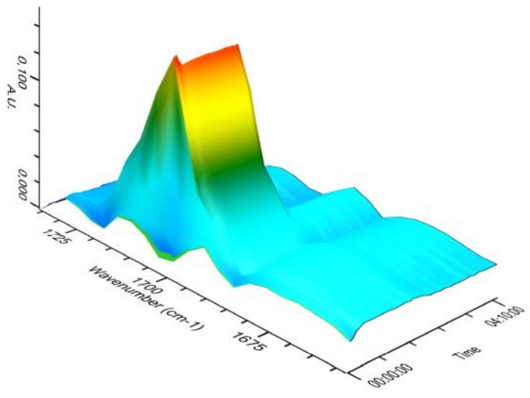

Supplementary Figure 4. Reaction without water addition monitored by *in situ* FT-IR. The stretching vibration of the carbonyl group in **2a** (1703 cm^{-1}) was monitored by *in situ* FT-IR. Reaction conditions: **1a** (0.02 mmol, 2 mol%), **2a** (1.00 mmol, 0.20 M), **3** (2.00 mmol, 0.40 M), 25 ℃, Et_2O (5.0 mL).

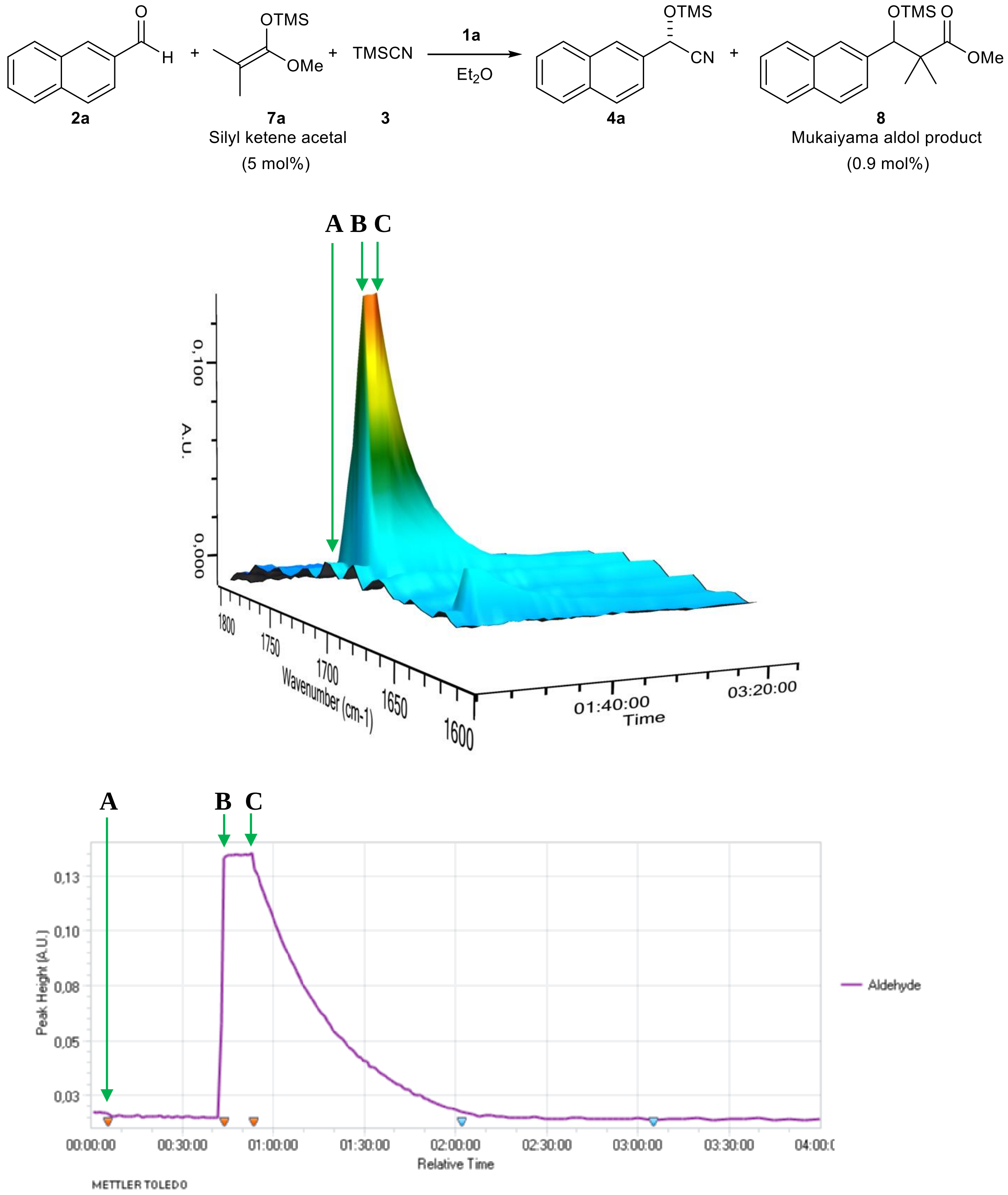

Supplementary Figure 5. Catalytic cyanosilylation using *in situ* generated reactive catalyst 1a-TMS. Silyl ketene acetal **7a** was used to react with precatalyst **1a** to generate reactive catalyst**.** The stretching vibration of the carbonyl group in **2a** (1703 cm^{-1}) was monitored by *in situ* FT-IR. Reaction conditions: **1a** (0.01 mmol, 1 mol%), 20 ℃, Et_2O (5.0 mL), **7a** (0.05 mmol, 5 mol%), **2a** (1.0 mmol, 0.20 M), **3** (1.6 mmol, 0.32 M)**. A: 1a**, **7a**, and Et_2O were added. **B: 2a** was added. **C: 3** was added. The reaction started immediately.

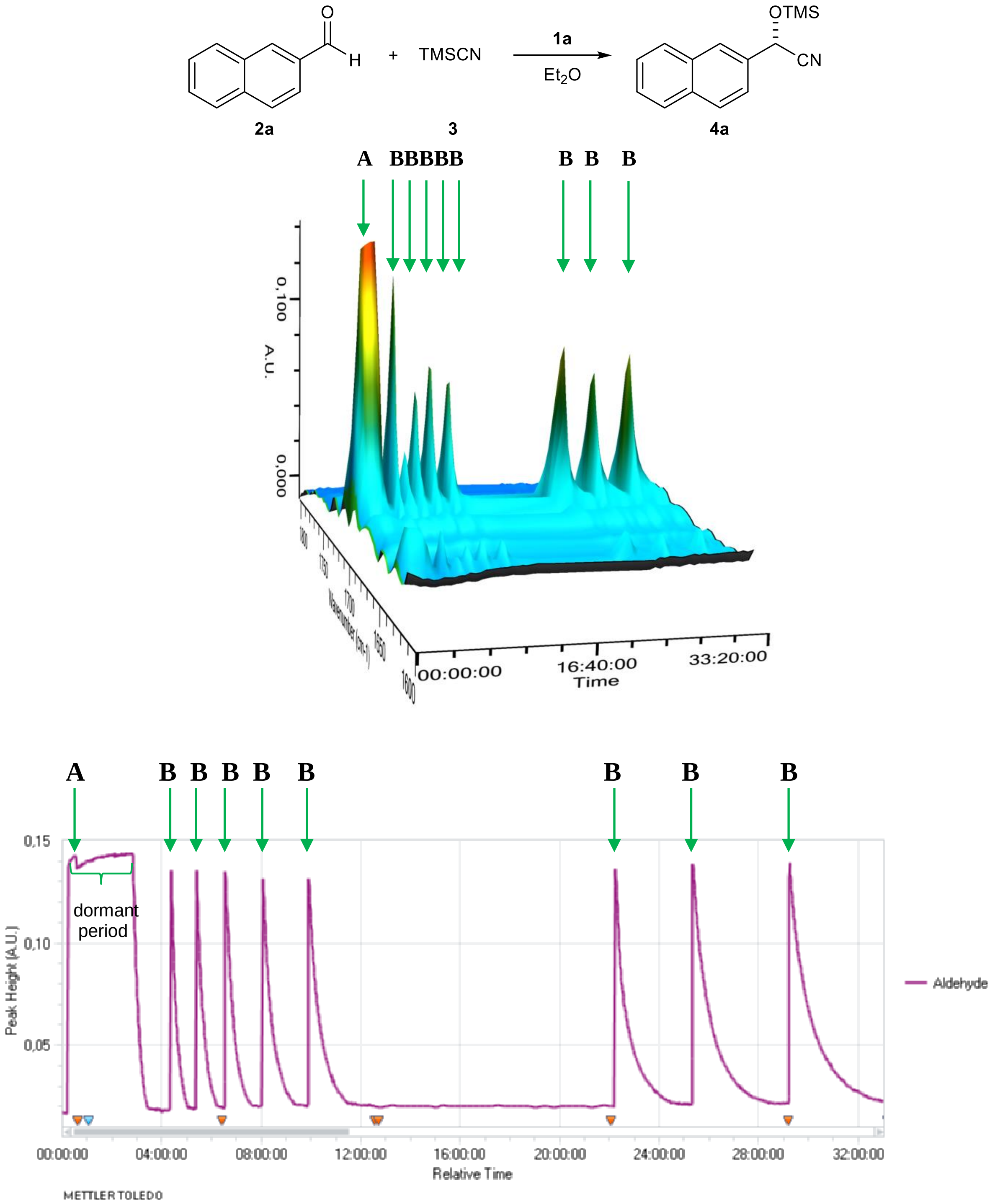

Supplementary Figure 6. Experiment on repetitive substrate addition cycles. the stretching vibration of the carbonyl group in **2a** (1703 cm^{-1}) was monitored by *in situ* FT-IR. **A:** **1st** catalytic cycle: **1a** (0.01 mmol, 1 mol%), 25 ℃, Et_2O (4.6 mL), **2a** (1.0 mmol, 1.0 equiv.), **3** (2.0 mmol, 2.0 equiv.). **B:** **2nd** to **9th** catalytic cycle: **2a** (1.0 mmol, 1.0 equiv.), **3** (1.0 mmol, 1.0 equiv.) for each cycle**.**

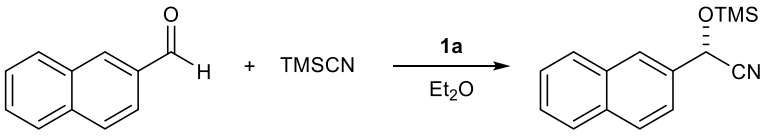

2a **3** **4a**

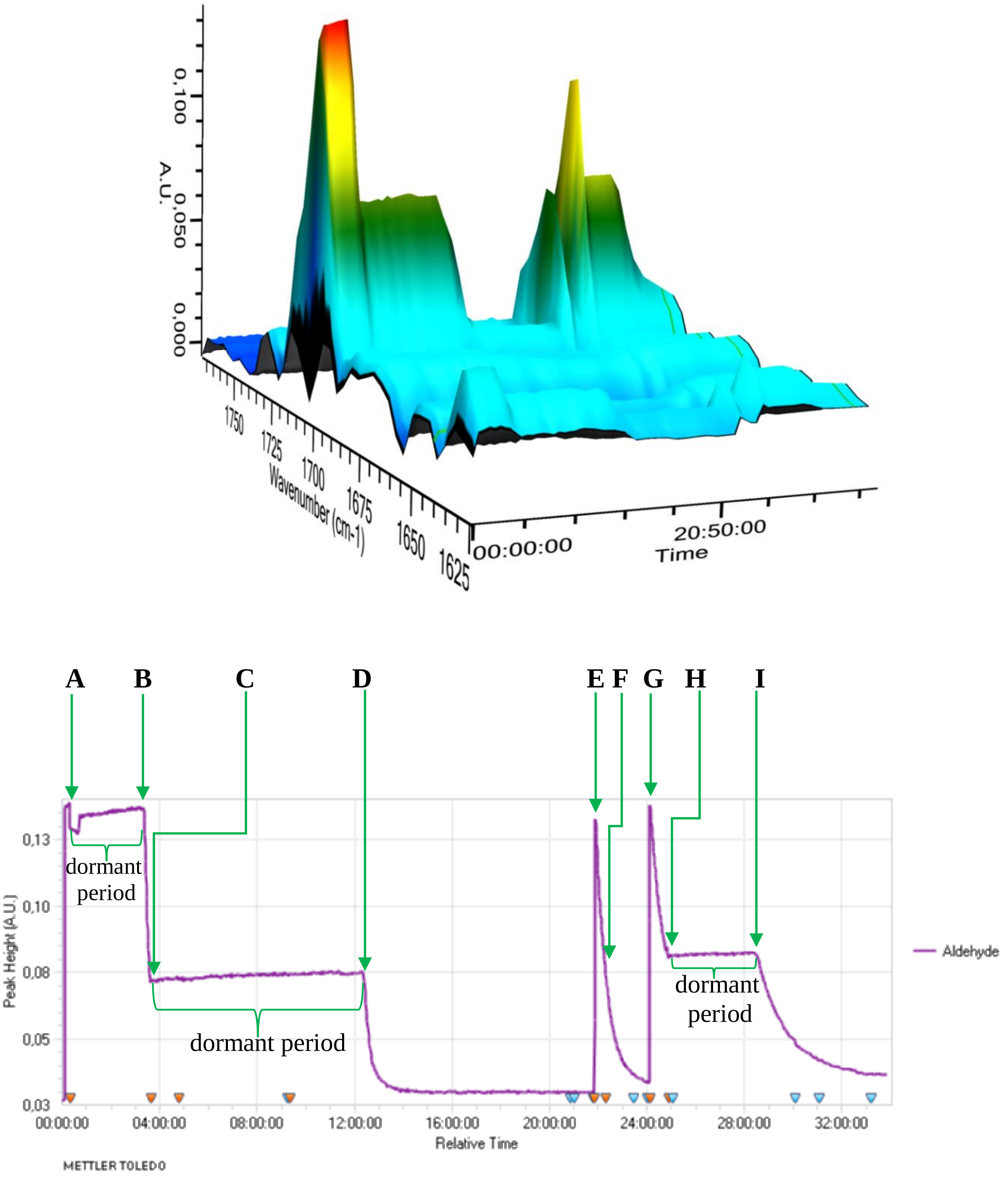

Supplementary Figure 7. The Influence of TMSOH 9 and $(TMS)_2O$ 10 on the Dormant Period. The stretching vibration of the carbonyl group in **2a** (1703 cm^{-1}) was monitored by *in situ* FT-IR. Reaction conditions: **1a** (0.01 mmol, 1 mol%), 20 ℃, Et_2O (5.0 mL), **2a** (1.0 mmol, 0.20 M), **3** (2.0 mmol, 0.40 M).

A: **1a**, **2a**, Et_2O and **3** were added.

B: Reaction started.

C: 10 mol% (1.8 µL) of H_2O and 20 mol% **3** were added at 60% conversion; reaction stopped.

D: Reaction restarted after approximately 8.7 hours.

E: 1.0 equiv. of **3** and 1.0 equiv. of **2a** were added after the first catalytic cycle was completed, reaction started immediately.

F: 20 mol% of $(TMS)_2O$ **10** was added at 58% conversion. No influence on the reaction progress was observed.

G: 1.0 equiv. of TMSCN and 1.0 equiv. of **2a** were added after the second catalytic cycle was completed, reaction started immediately.

H: 10 mol% of TMSOH **9** were added at 54% conversion; reaction stopped.

I: Reaction started again after approximately 3.7 hours.

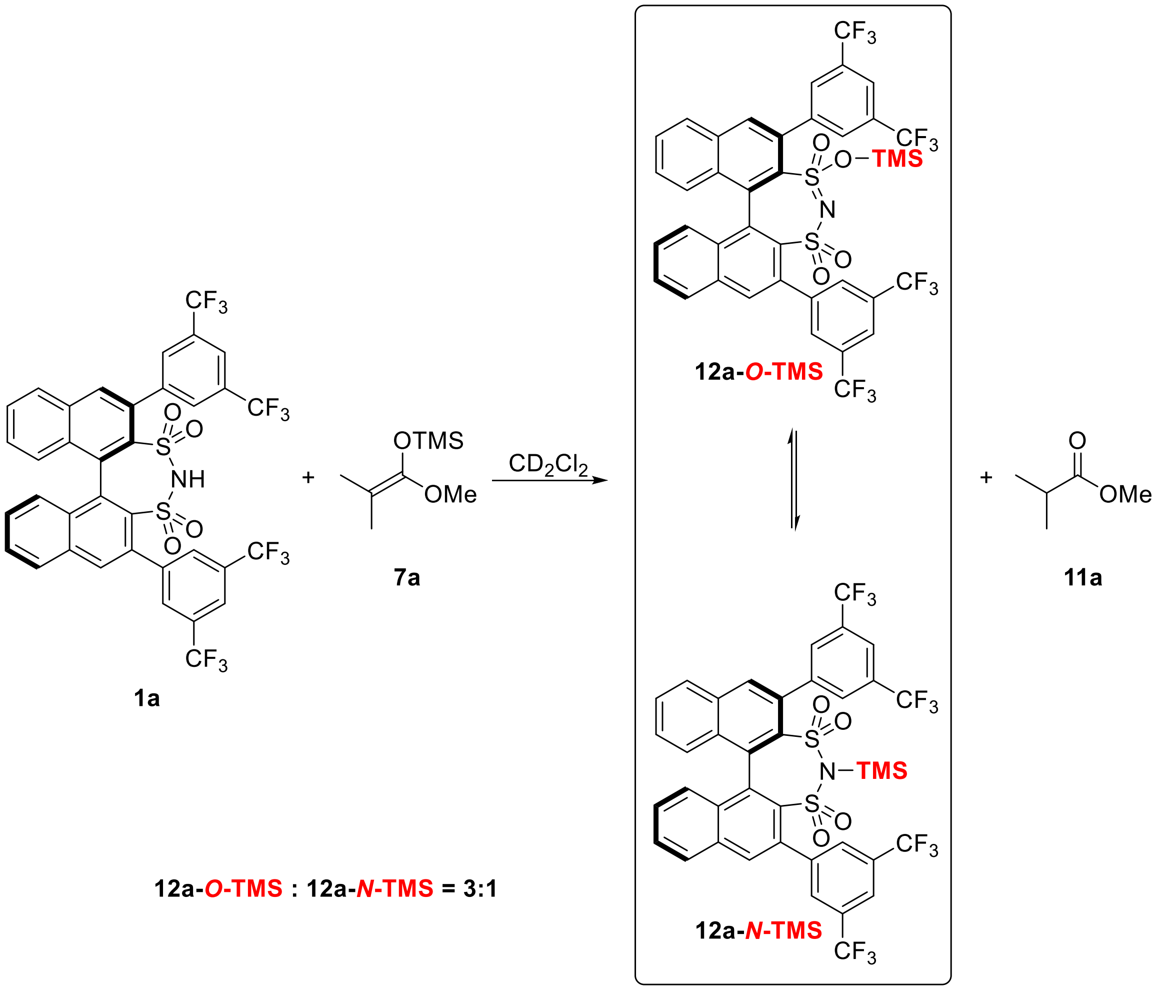

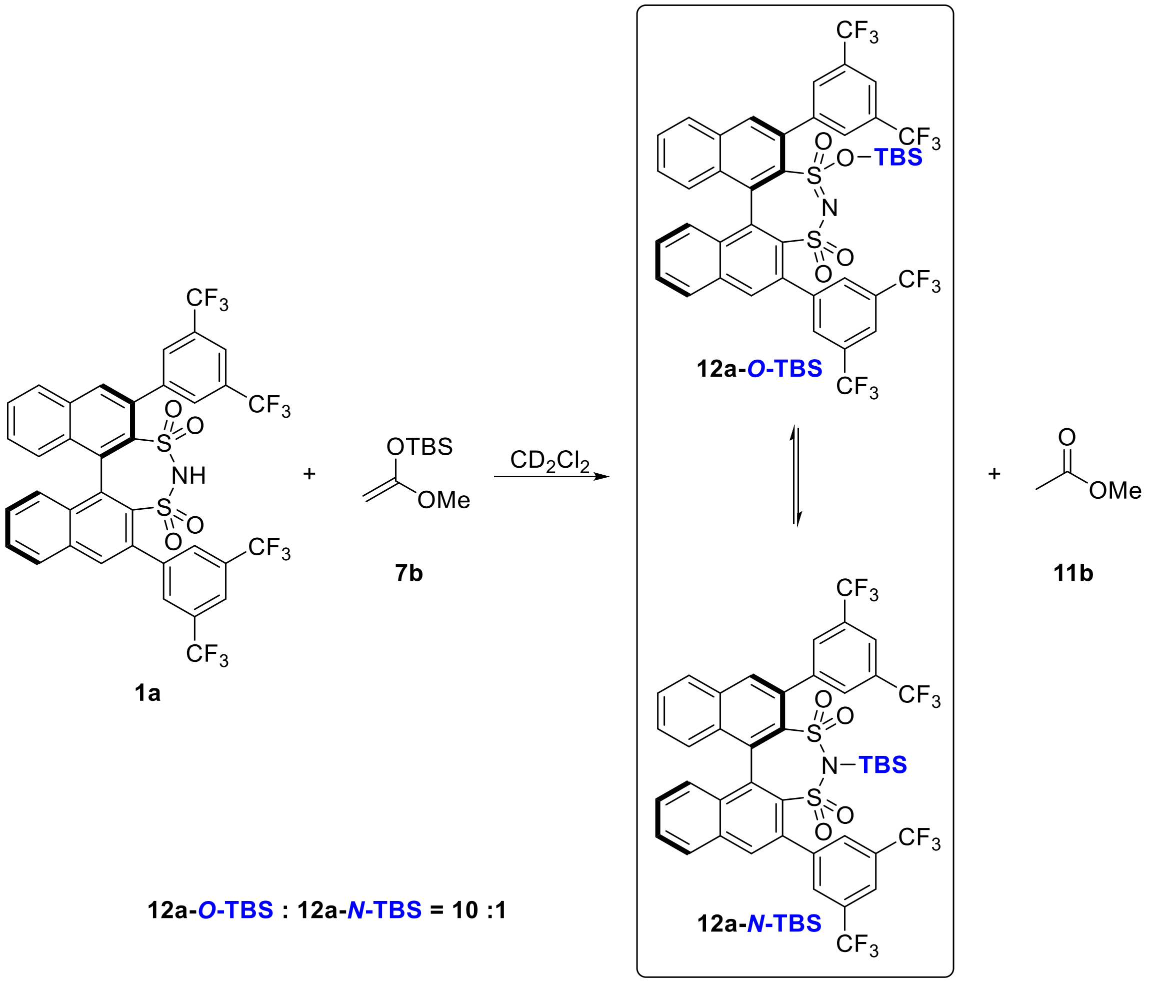

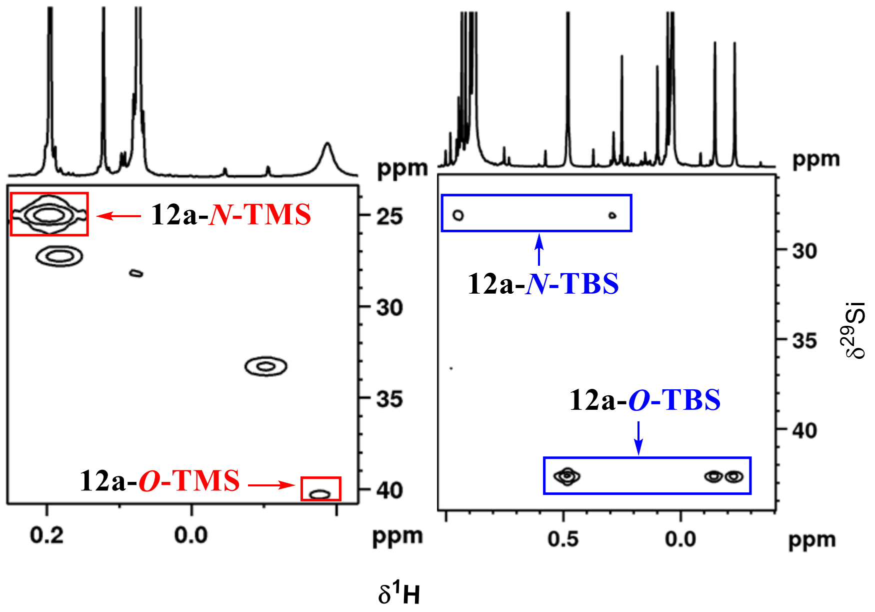

Supplementary Figure 8. ^{1}H-^{29}Si-HMBC Spectra. Left: **12a-*O*-TMS** and **12a-*N*-TMS**. Right: **12a-*O*-TBS** and **12a-*N*-TBS.**

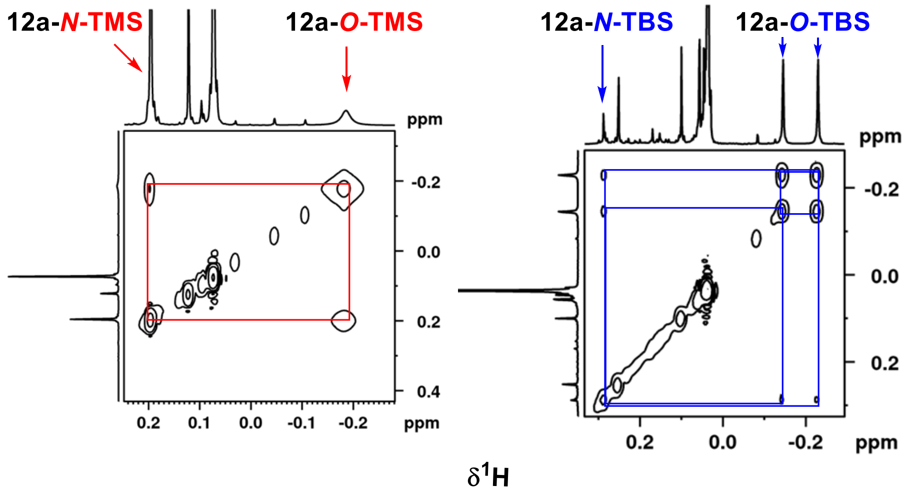

Supplementary Figure 9. 2D-EXSY-Spectra. Left: exchange of **12a-*N*-TMS** and **12a-*O*-TMS**. Right: exchange of **12a-*N*-TBS** and **12a-*O*-TBS**.

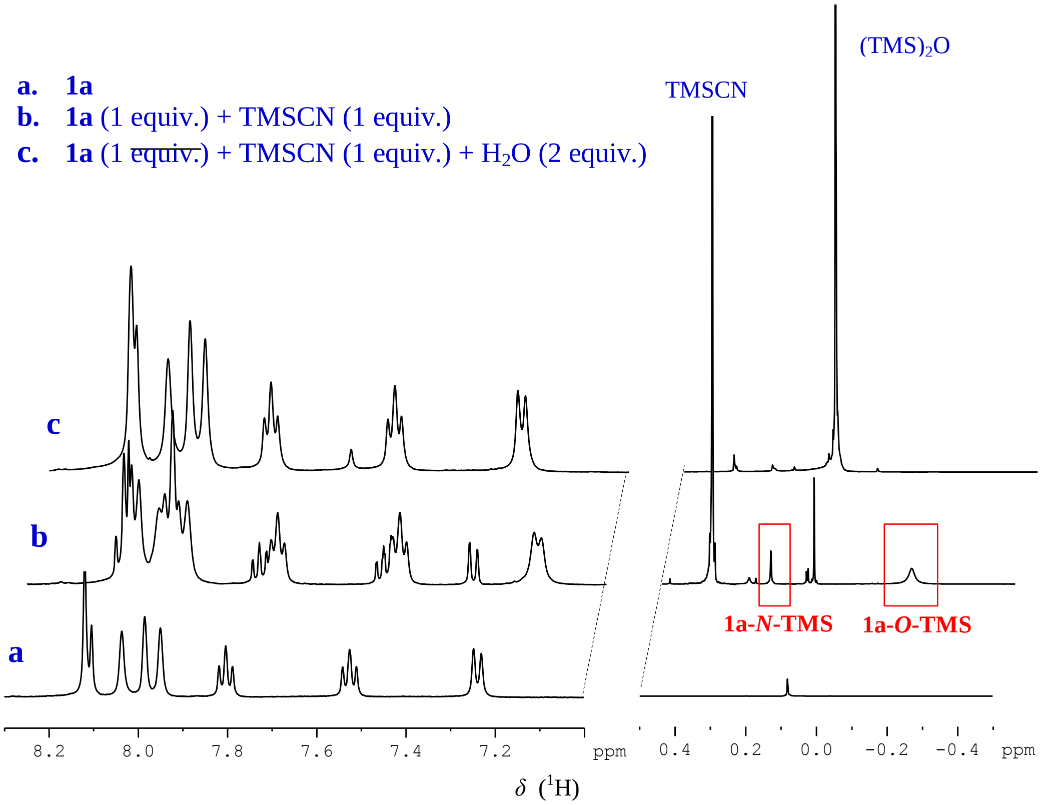

^{1}H-NMR Spectrum of **1a**, (**1a** + TMSCN) and (**1a** + TMSCN + H_2O)

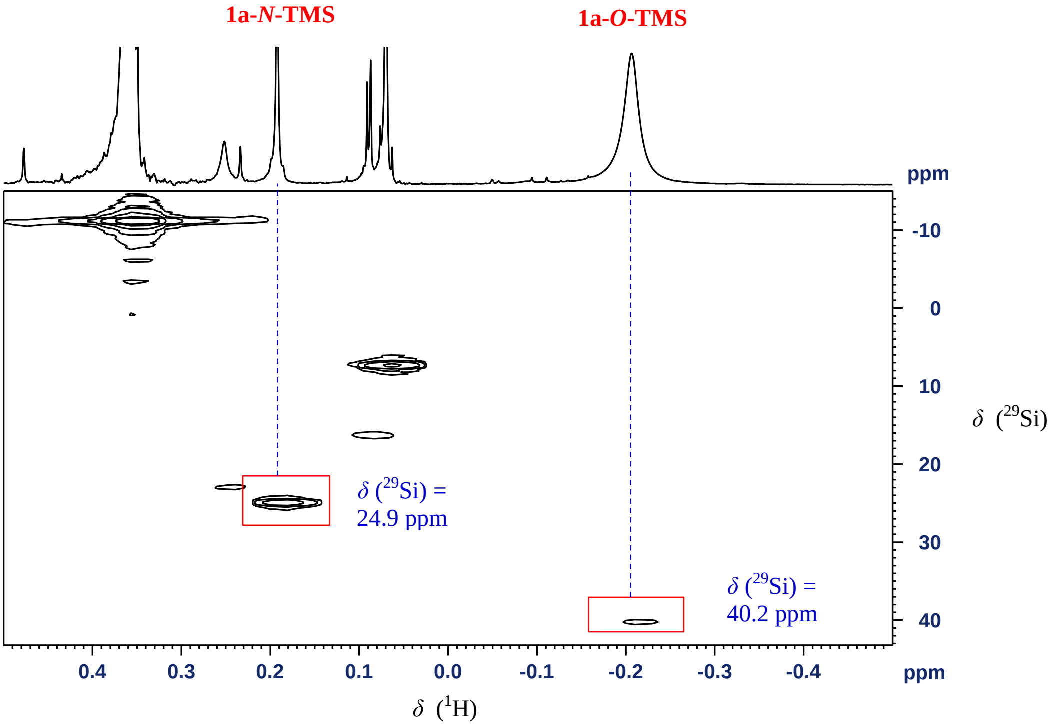

^{1}H-^{29}Si-HMBC Spectrum of **b**. (**1a** + TMSCN)

Supplementary Figure 10. NMR study of the build-up and decay of the 1a-TMS.

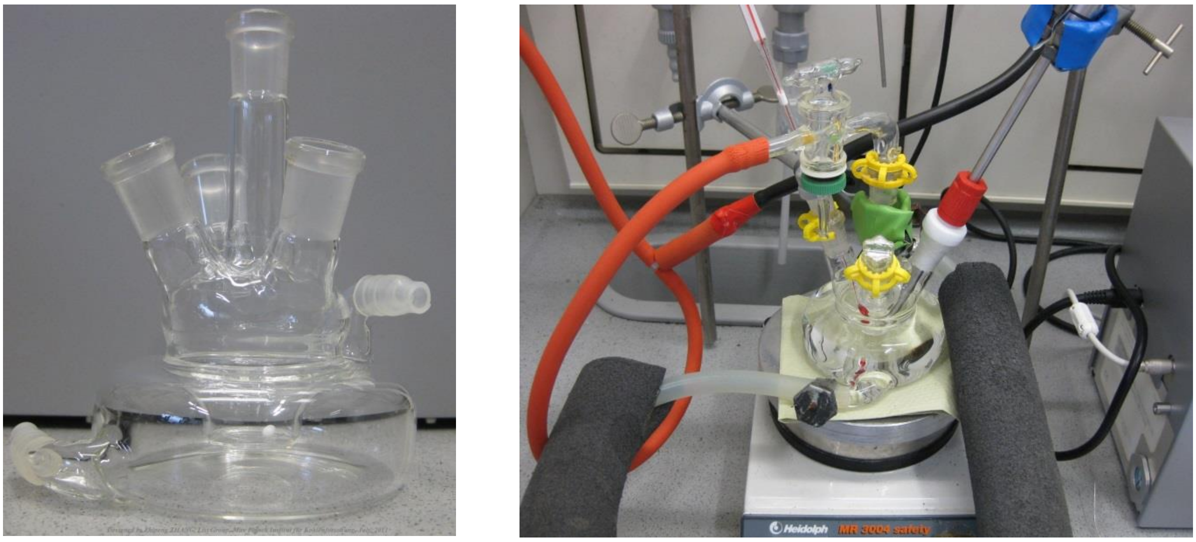

Supplementary Figure 11. Newly designed reactor for the *in situ* FT-IR measurements

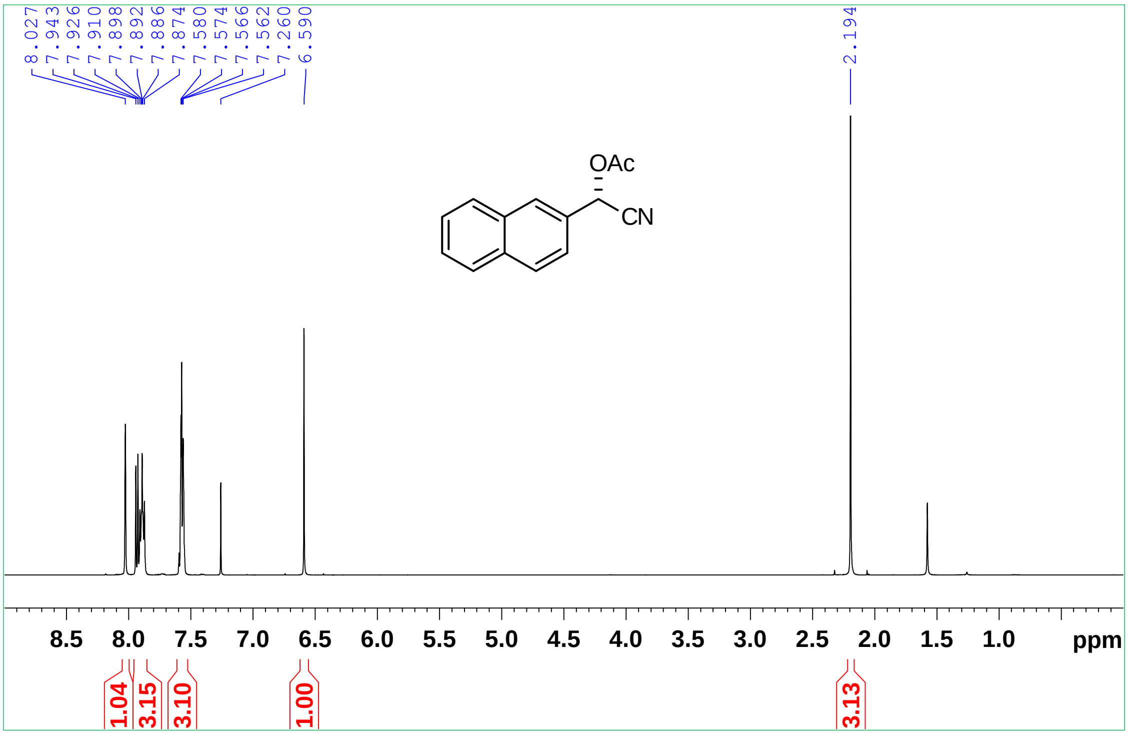

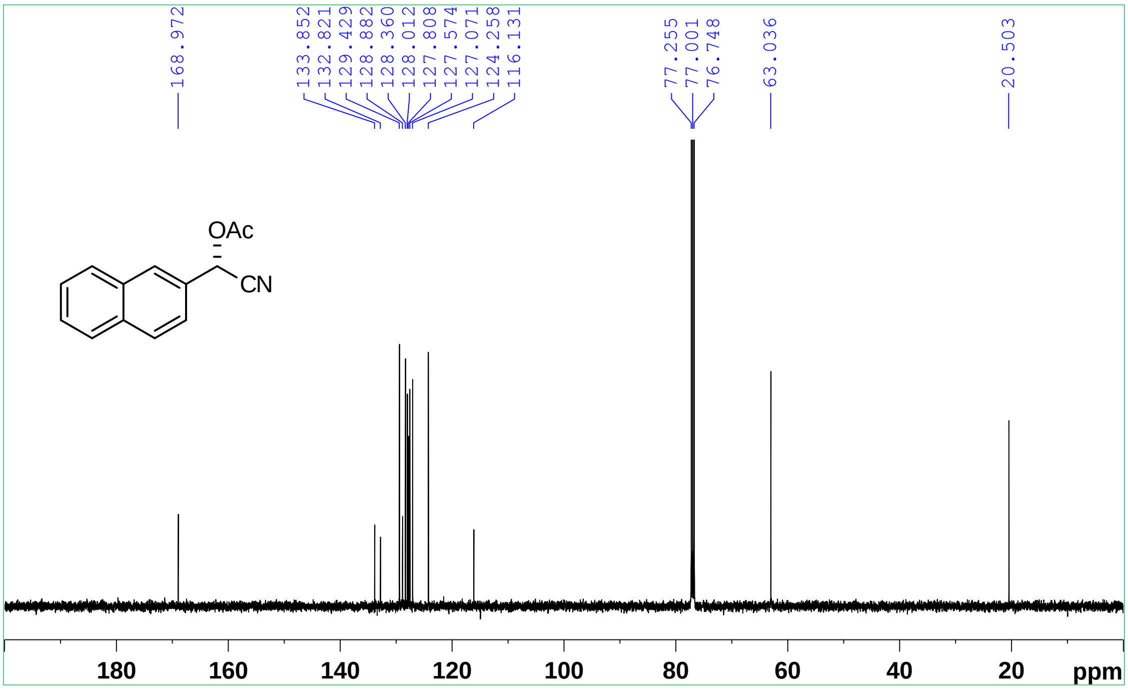

Supplementary Figure 12. **^{1}H and ^{13}C NMR spectra of (*S*)-cyano(naphthalene-2-yl) methyl acetate 5a**

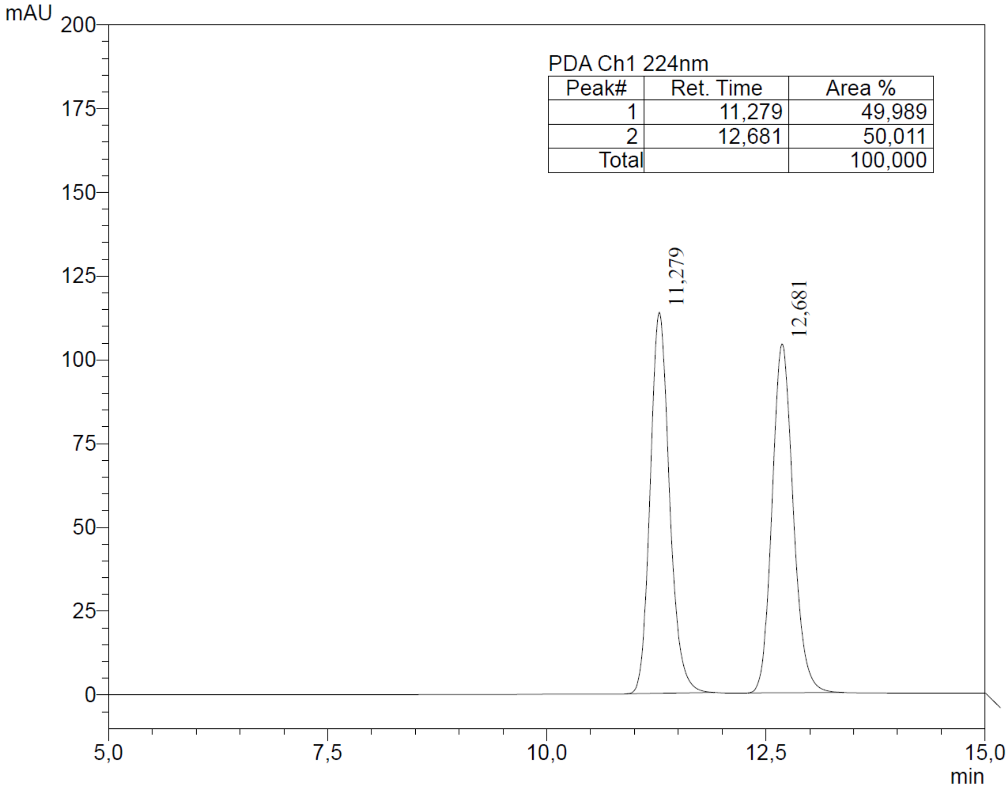

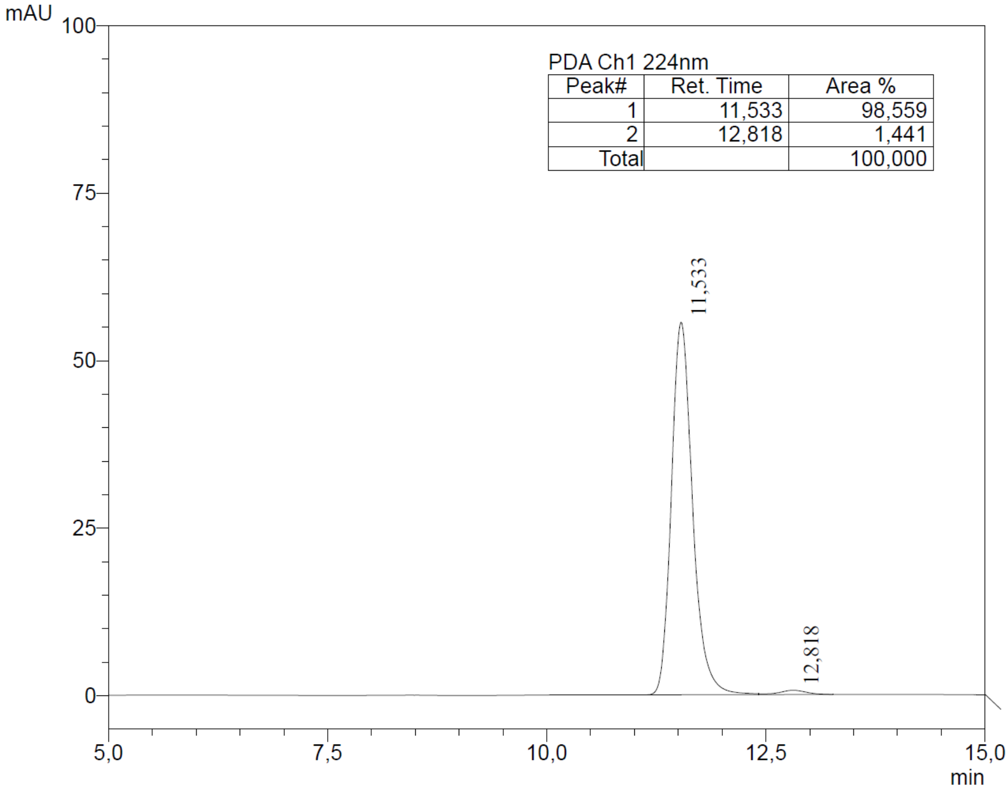

Supplementary Figure 13. HPLC spectra of (*S*)-cyano(naphthalene-2-yl)methyl acetate 5a. Diacel Chiralpak AD-3, *n*-heptane:isopropanol = 99:1, flow = 1.0 mL/min, 25 ℃, λ = 224 nm, t_R(major) = 11.5 min, t_R(minor) = 12.8 min, e.r. = 98.5:1.5.

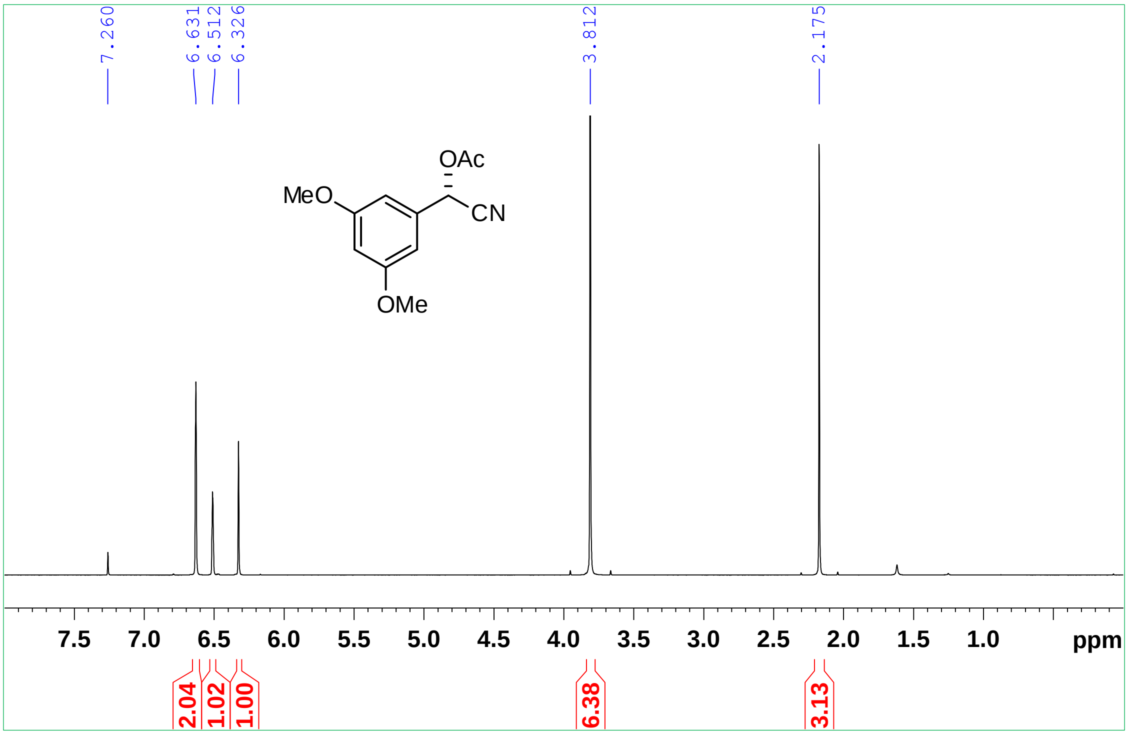

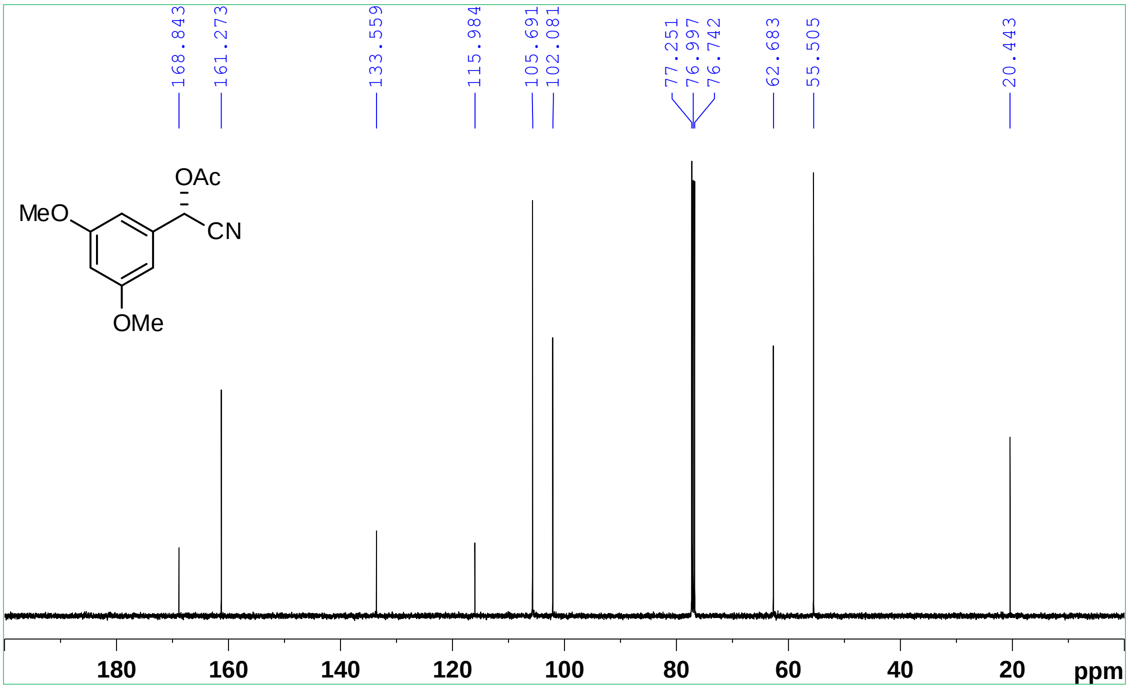

Supplementary Figure 14. ^{1}H and ^{13}C NMR spectra of (*S*)-cyano(3,5-dimethoxyphenyl) methyl acetate 5b

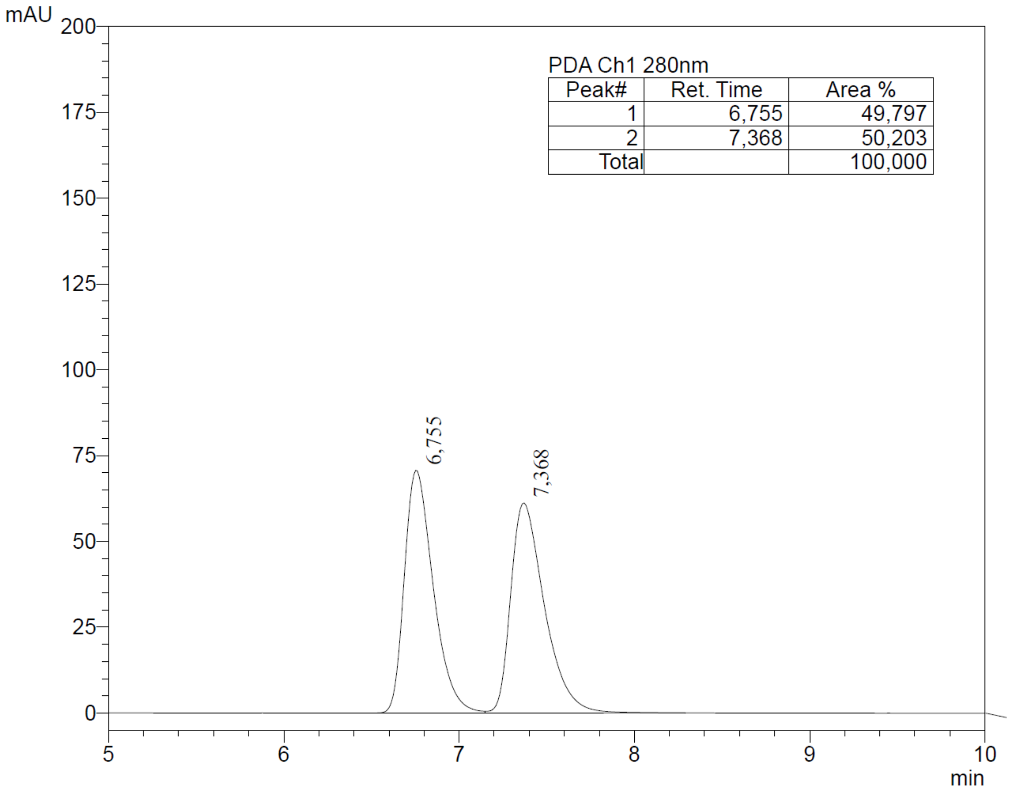

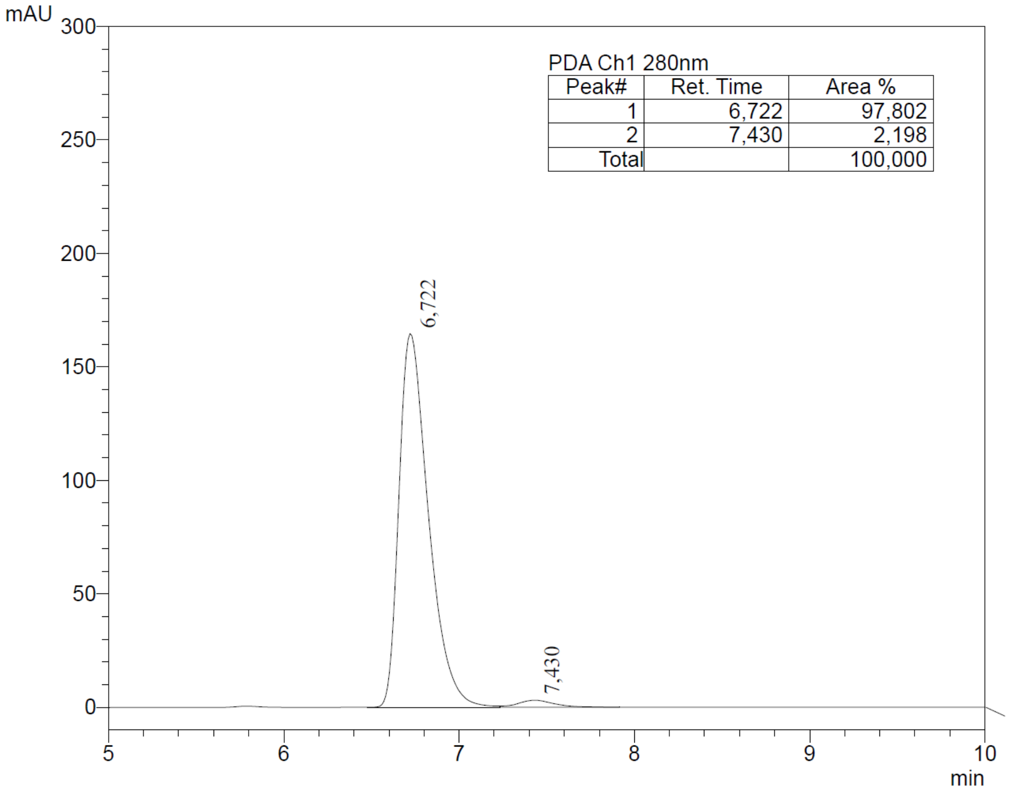

Supplementary Figure 15. HPLC spectra of (*S*)-cyano(3,5-dimethoxyphenyl)methyl acetate 5b. Diacel Chiralpak OD-3, *n*-heptane:isopropanol = 90:10, flow = 1.0 mL/min, 25 ℃, λ = 280 nm, t_R(major) = 6.7 min, t_R(minor) = 7.4 min, e.r. = 98:2.

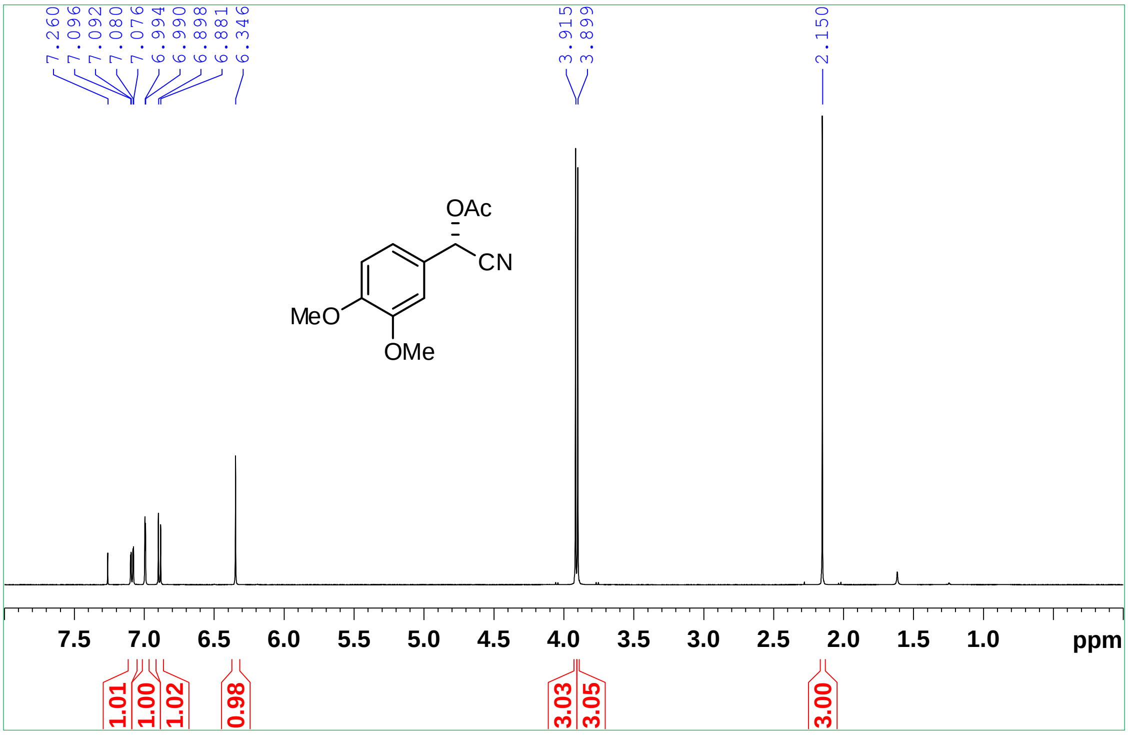

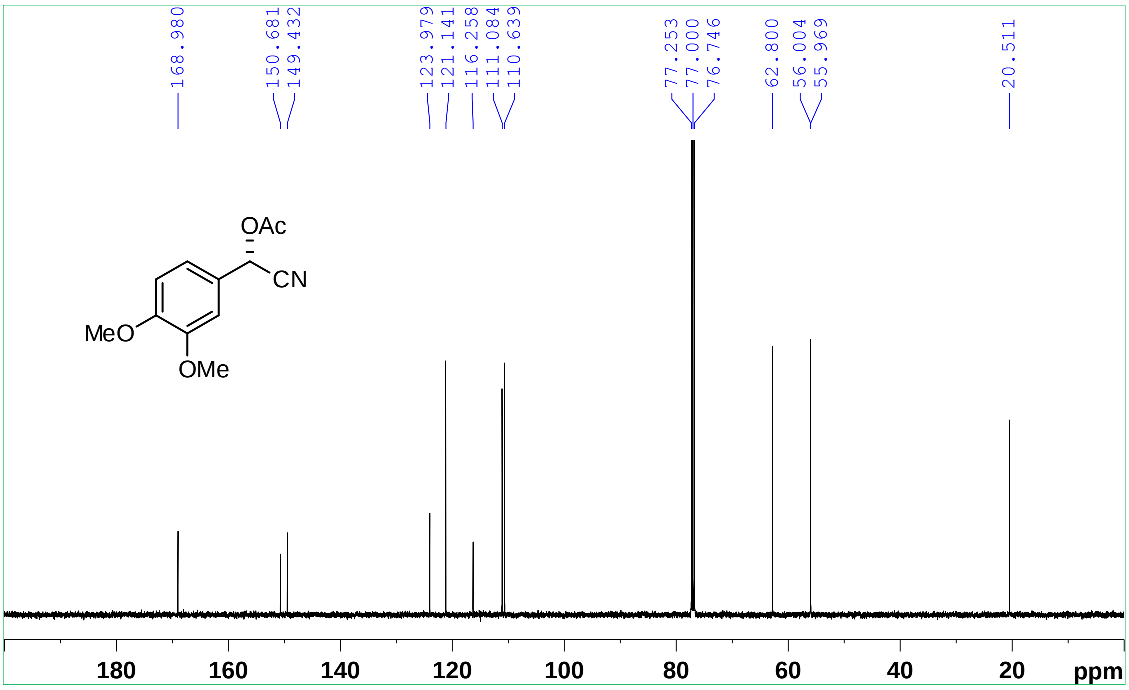

Supplementary Figure 16. **^{1}H and ^{13}C NMR spectra of (*S*)-cyano(3,4-dimethoxyphenyl) methyl acetate 5c**

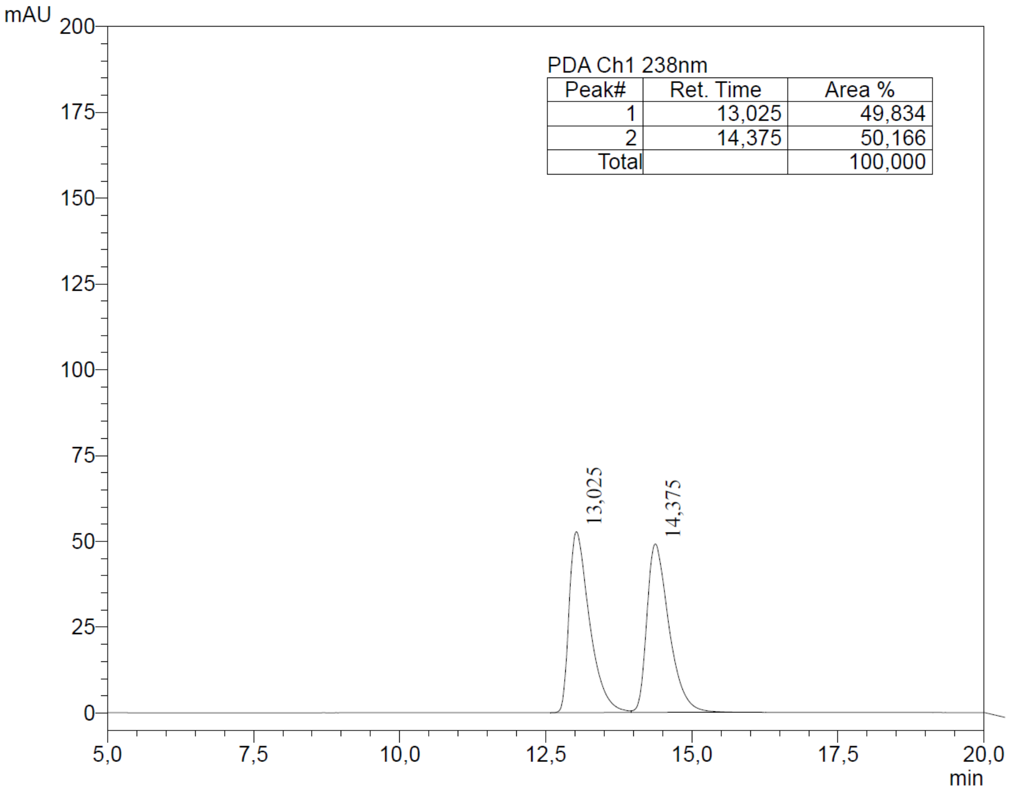

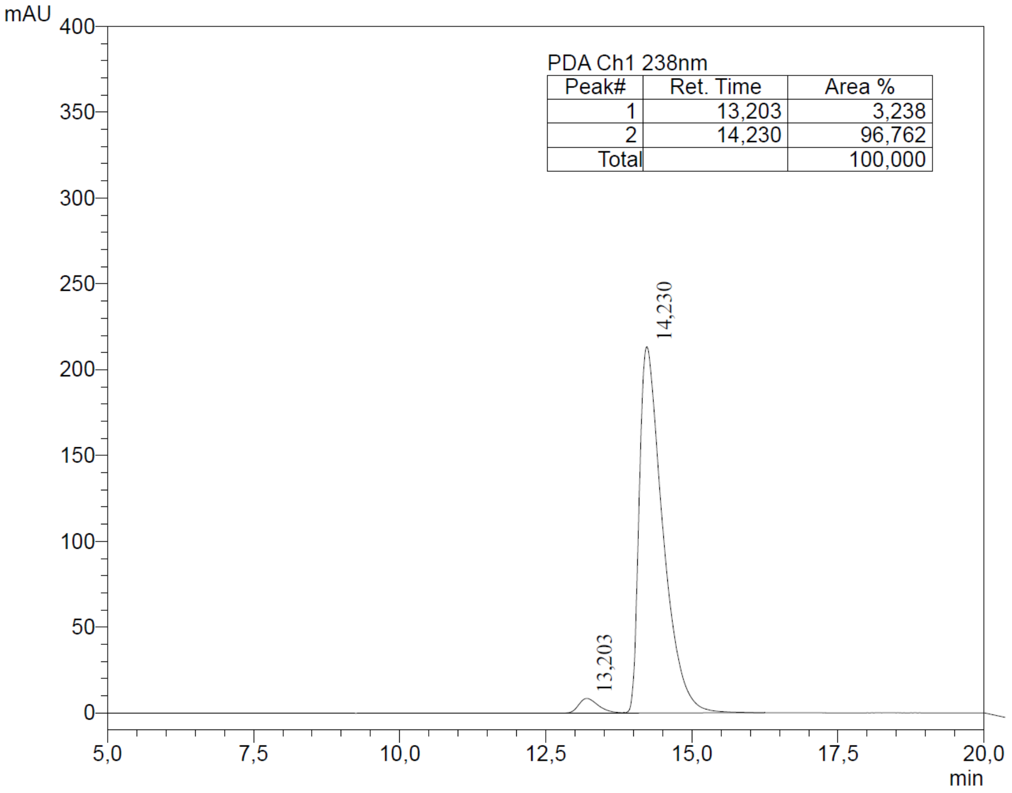

Supplementary Figure 17. HPLC spectra of (*S*)-cyano(3,4-dimethoxyphenyl)methyl acetate 5c. Diacel Chiralpak OD-3, *n*-heptane:isopropanol = 95:5, flow = 1.0 mL/min, 25 ℃, λ = 238 nm, t_R(minor) = 13.2 min, t_R(major) = 14.2 min, e.r. = 97:3.

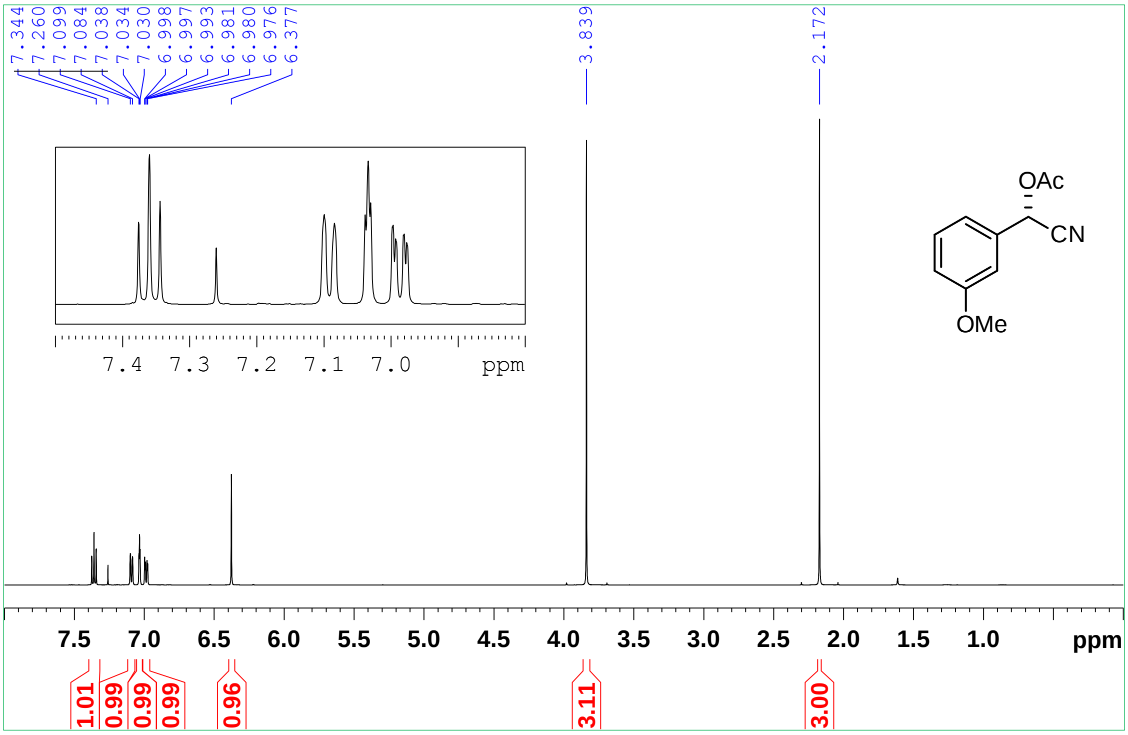

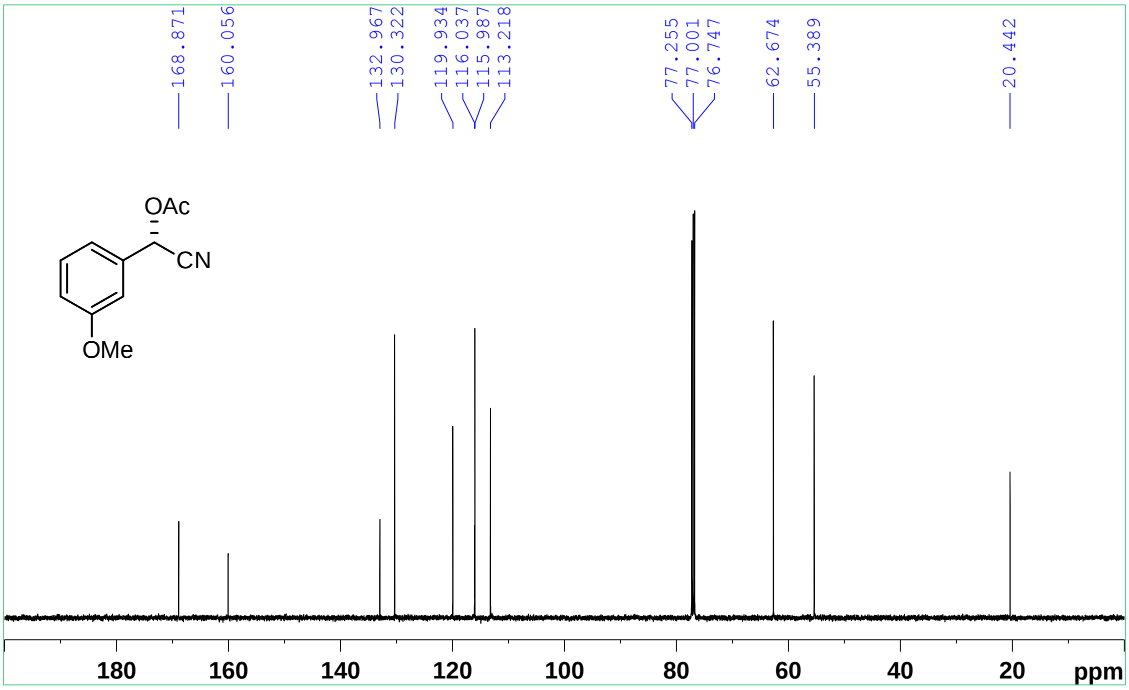

Supplementary Figure 18. ^{1}**H and** ^{13}**C NMR spectra of (*S*)-cyano(3-methoxyphenyl) methyl acetate 5d**

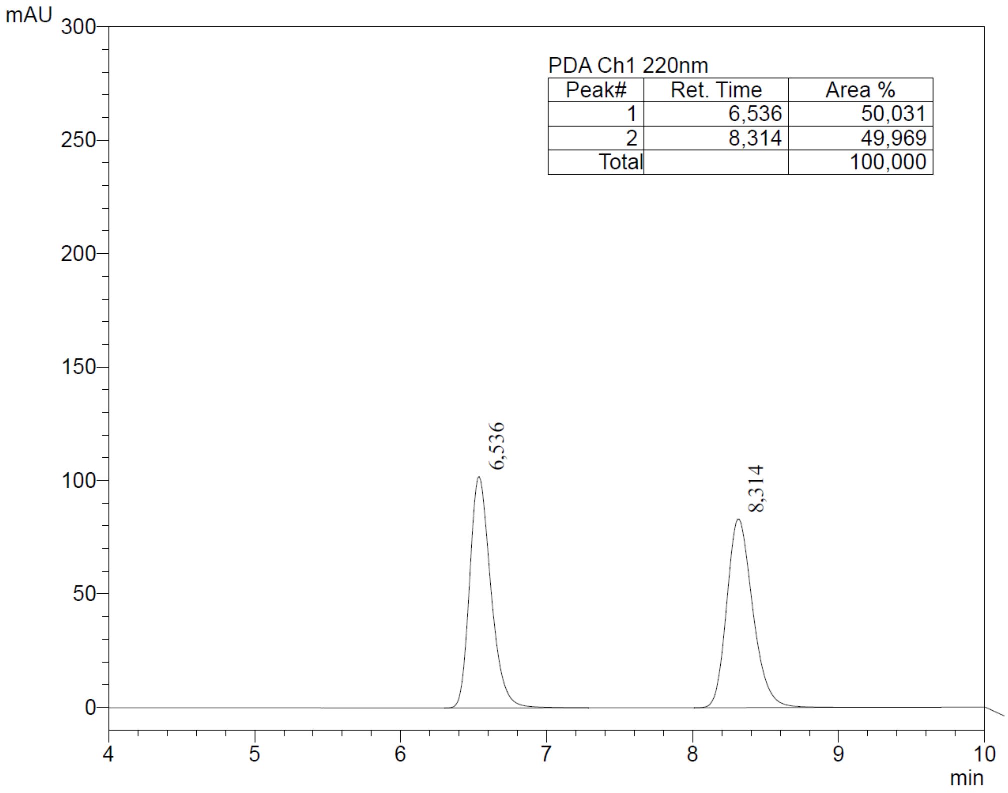

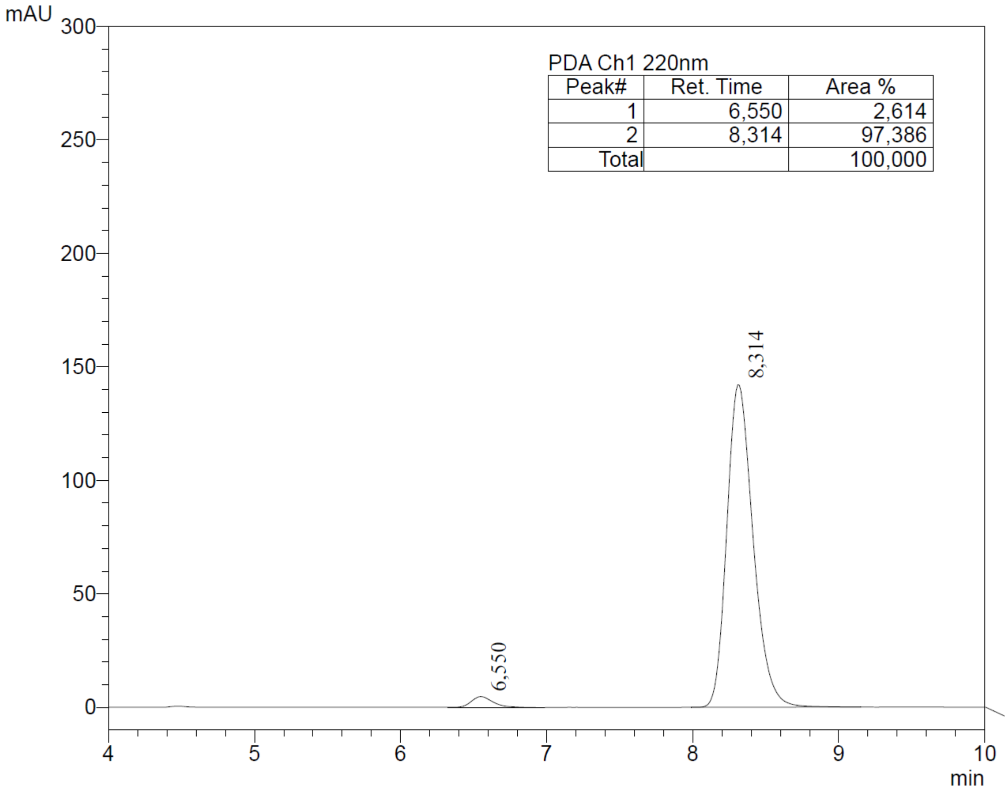

Supplementary Figure 19. HPLC spectra of (*S*)-cyano(3-methoxyphenyl)methyl acetate 5d. Diacel Chiralpak OD-3, *n*-heptane:isopropanol = 90:10, flow = 1.0 mL/min, 25 ℃, λ = 220 nm, t_R(minor) = 6.6 min, t_R(major) = 8.3 min, e.r. = 97.5:2.5.

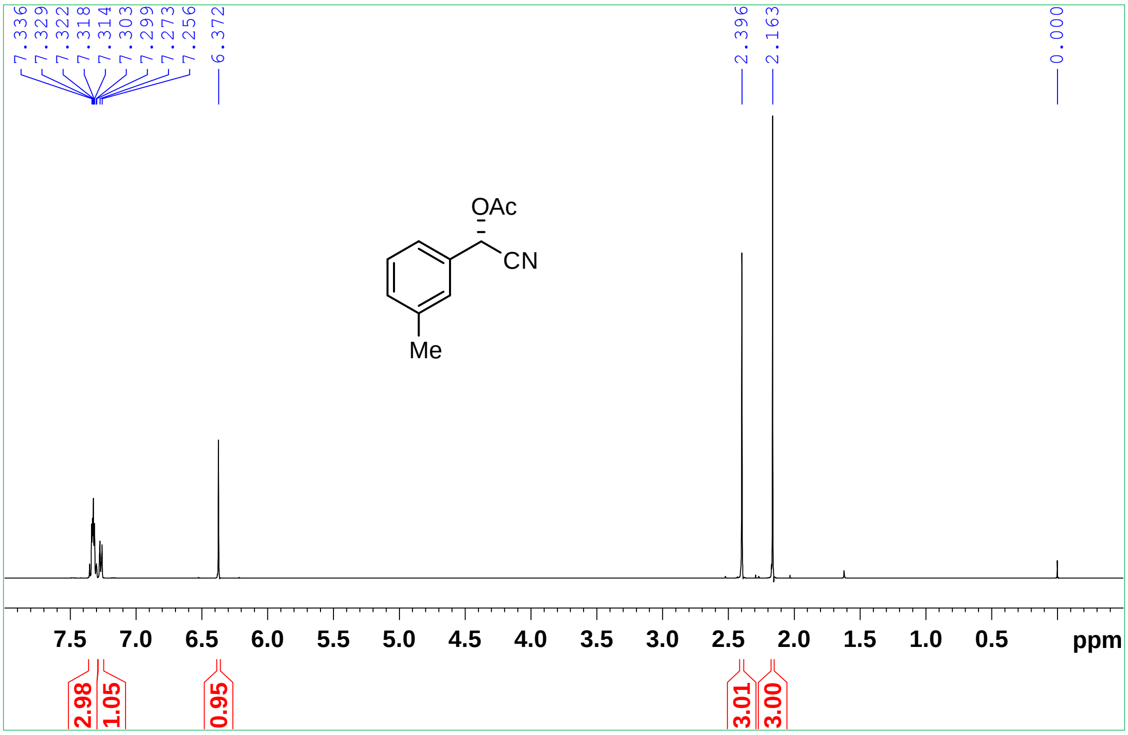

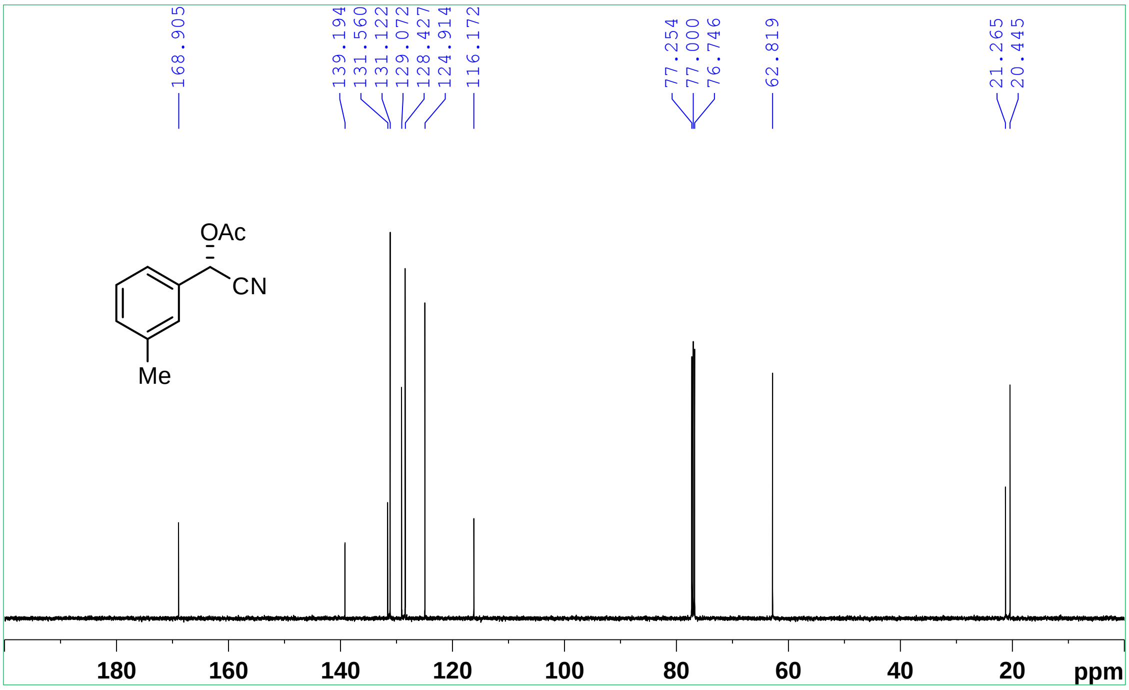

Supplementary Figure 20. ^{1}**H and** ^{13}**C NMR spectra of (*S*)-cyano(*m*-tolyl)methyl acetate 5e**

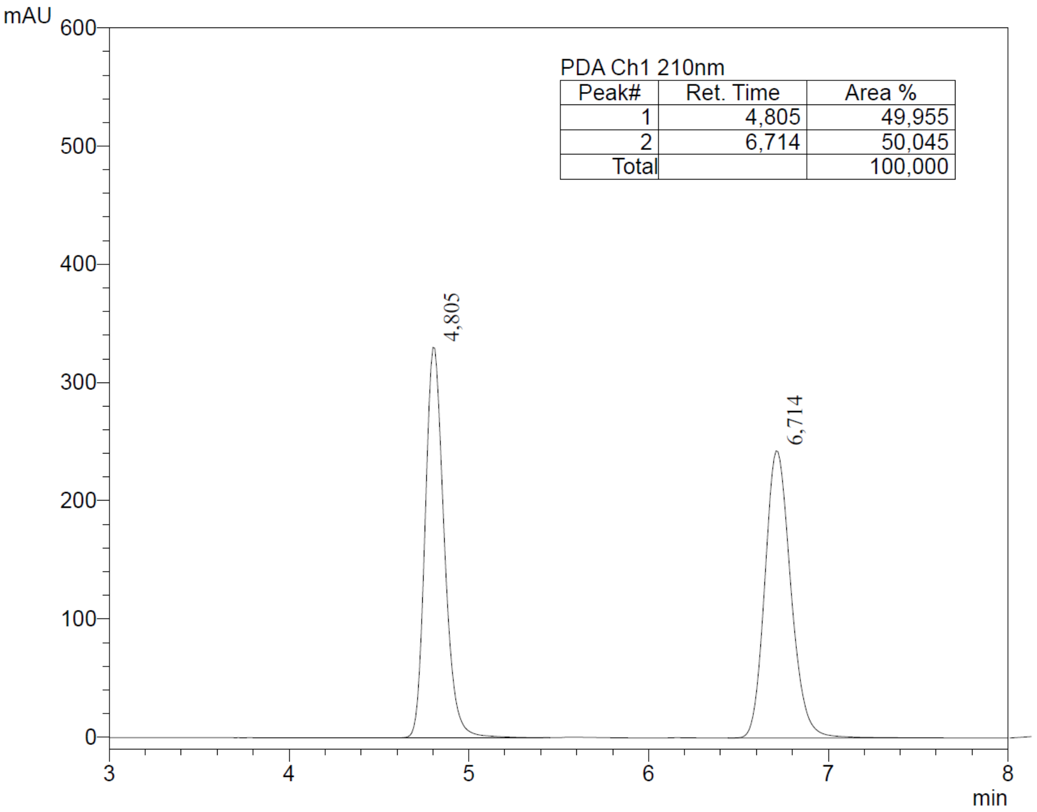

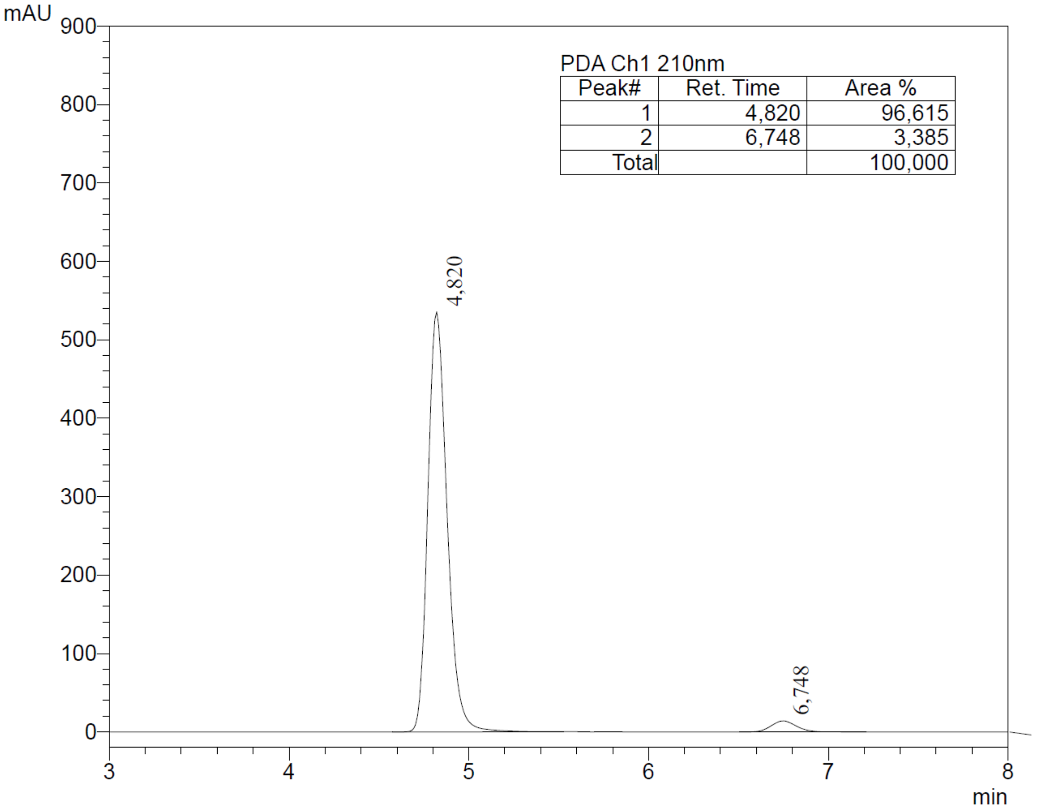

Supplementary Figure 21. HPLC spectra of (*S*)-cyano(*m*-tolyl)methyl acetate 5e. Diacel Chiralpak AS-3, *n*-heptane:isopropanol = 90:10, flow = 1.0 mL/min, 25 ℃, λ = 210 nm, t_R(major) = 4.8 min, t_R(minor) = 6.8 min, e.r. = 96.5:3.5.

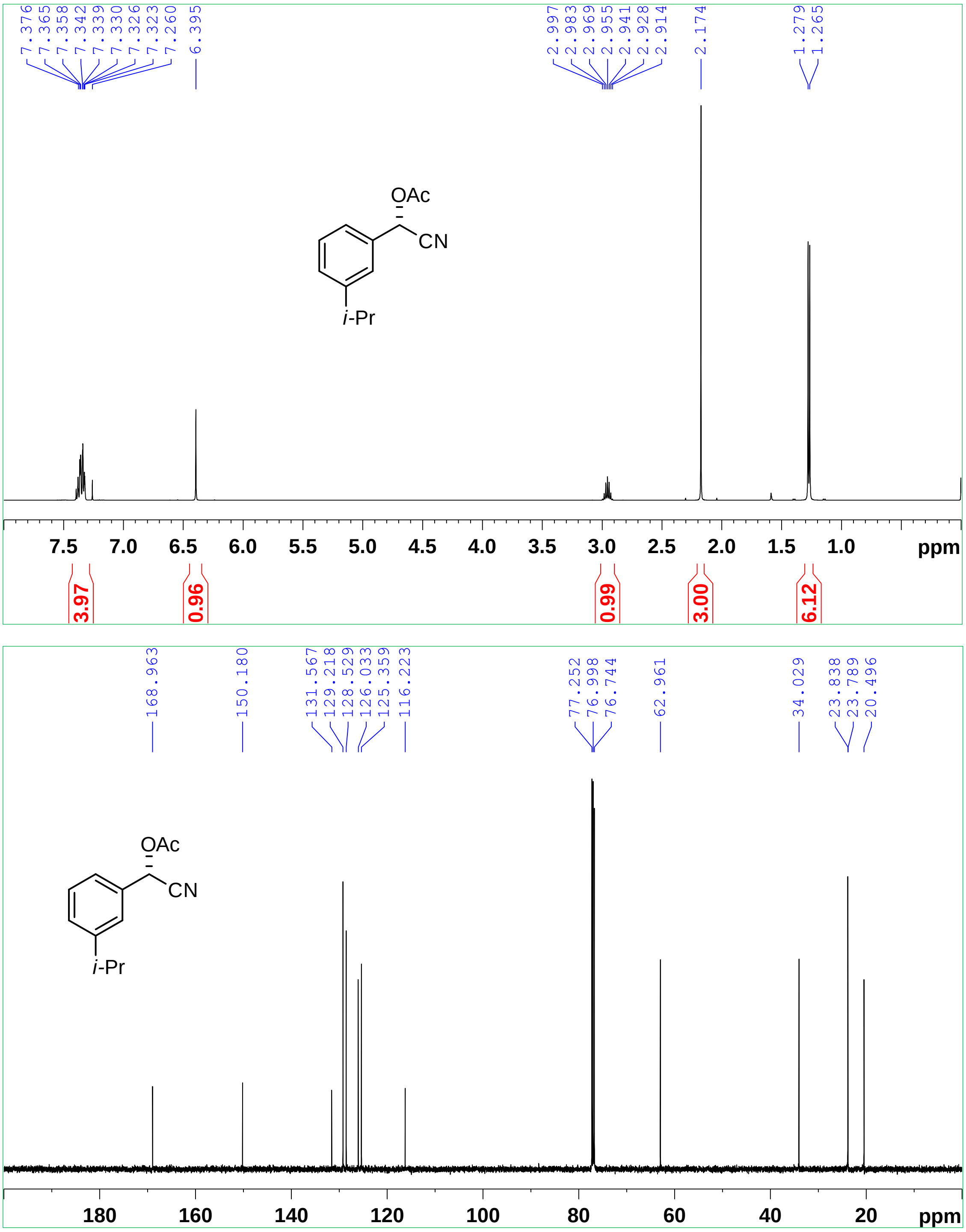

Supplementary Figure 22. **^{1}H and ^{13}C NMR spectra of (*S*)-cyano(3-isopropylphenyl) methyl acetate 5f**

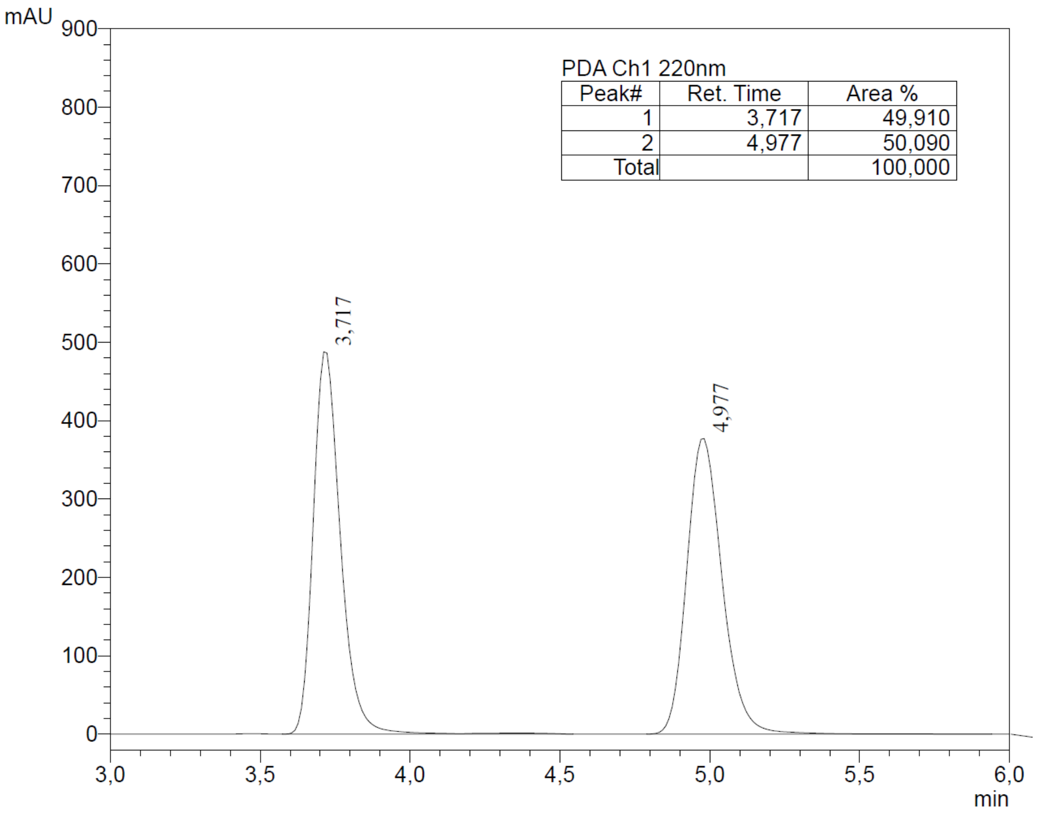

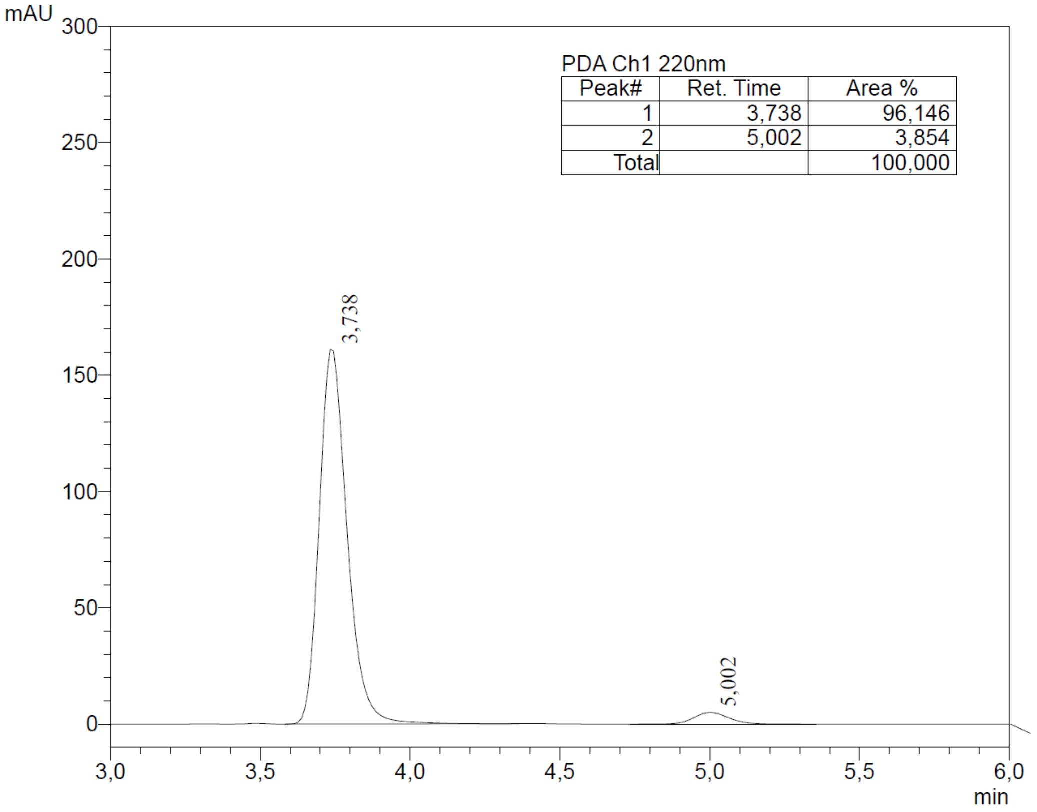

Supplementary Figure 23. HPLC spectra of (*S*)-cyano(3-isopropylphenyl)methyl acetate 5f. Diacel Chiralpak AS-3, *n*-heptane:isopropanol = 90:10, flow = 1.0 mL/min, 25 ℃, λ = 220 nm, t_R(major) = 3.7 min, t_R(minor) = 5.0 min, e.r. = 96:4.

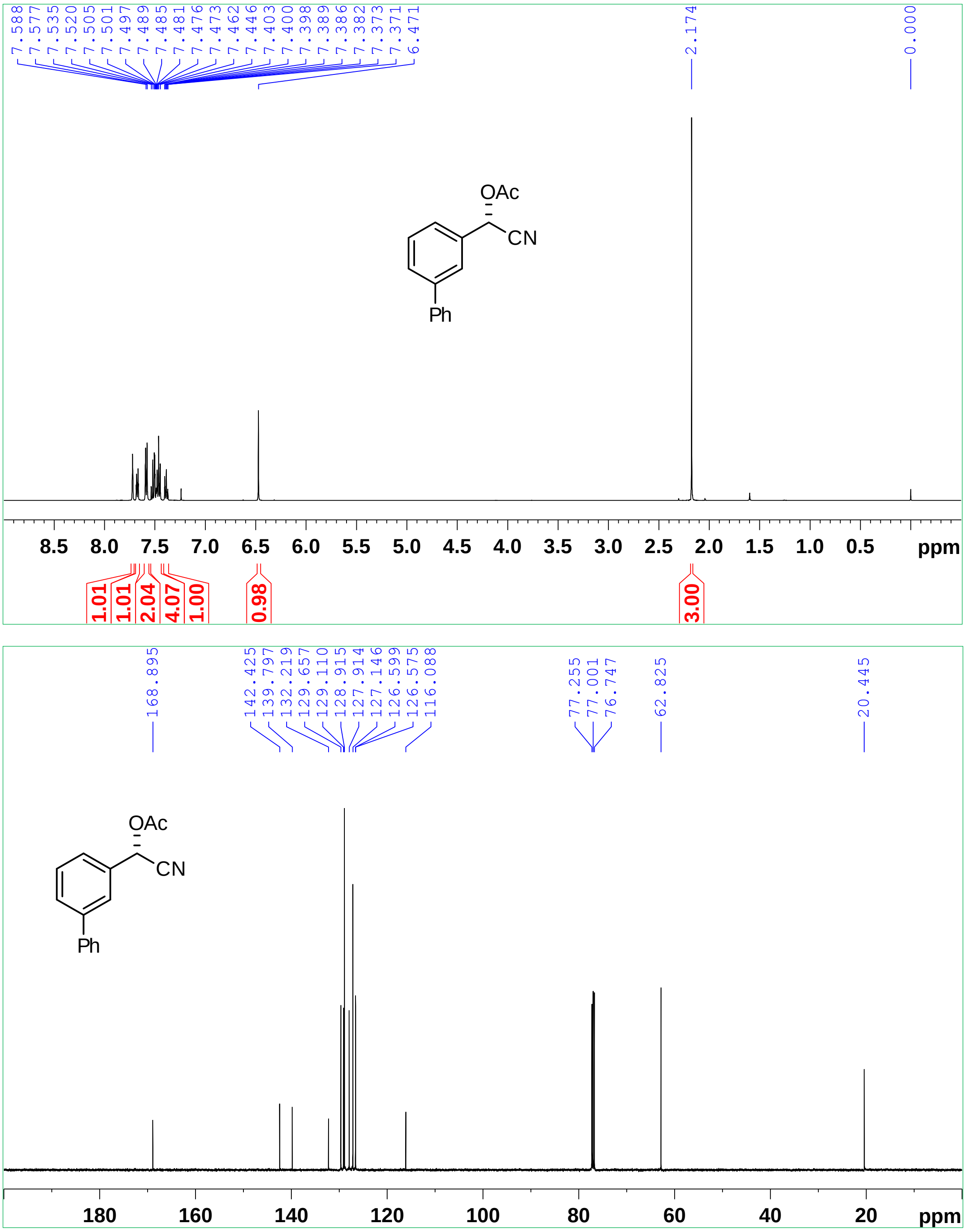

Supplementary Figure 24. ^{1}**H and** ^{13}**C NMR spectra of (*S*)-[1,1'-biphenyl]-3-yl(cyano) methyl acetate 5g**

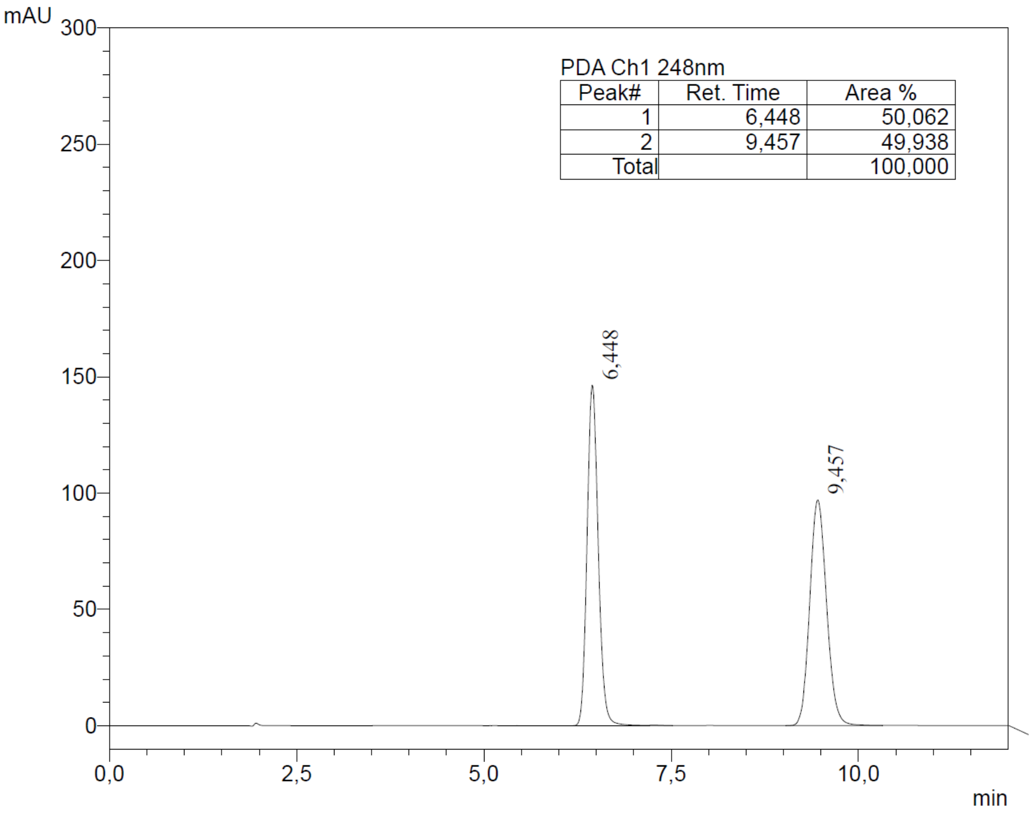

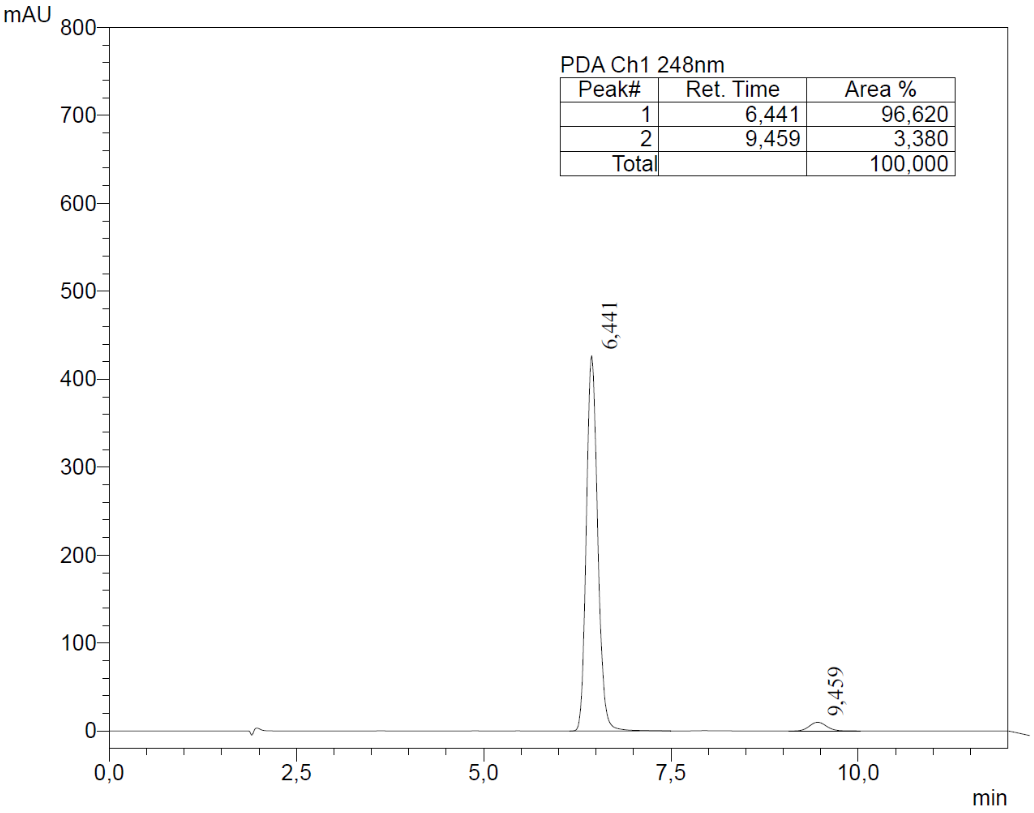

Supplementary Figure 25. HPLC spectra of (*S*)-[1,1'-biphenyl]-3-yl(cyano)methyl acetate 5g. Diacel Chiralpak AS-3, *n*-heptane:isopropanol = 90:10, flow = 1.0 mL/min, 25 ℃, λ = 248 nm, t_R(major) = 6.4 min, t_R(minor) = 9.5 min, e.r. = 96.5:3.5.

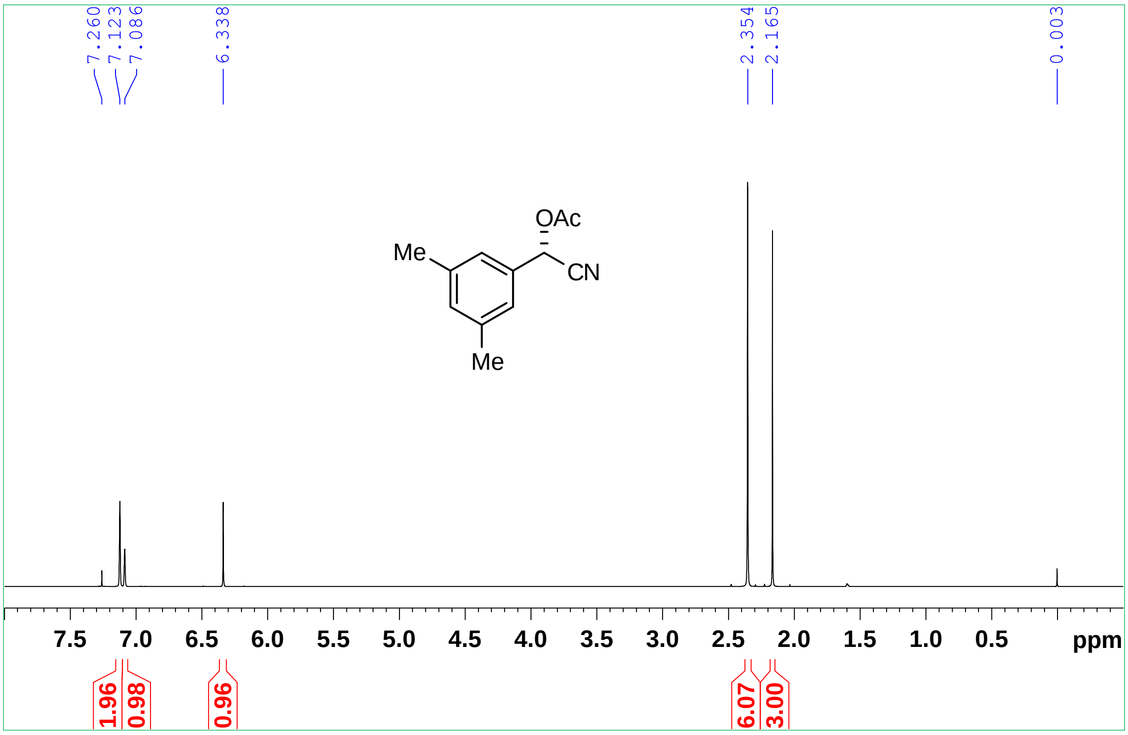

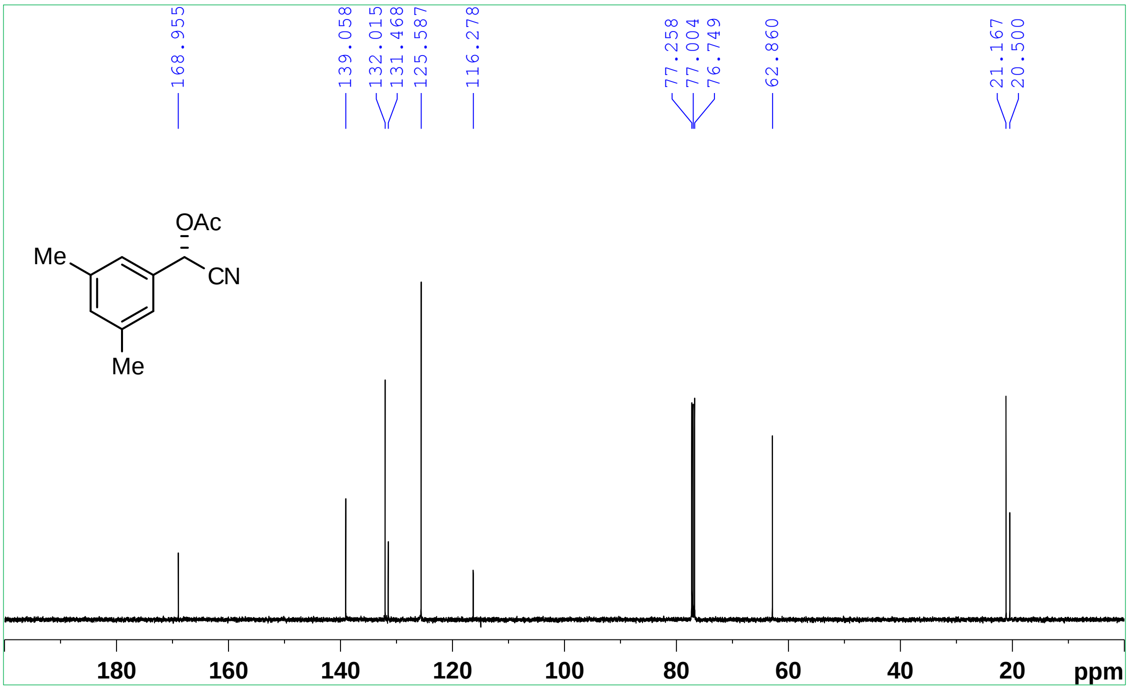

Supplementary Figure 26. ^{1}H and ^{13}C NMR spectra of (*S*)-cyano(3,5-dimethylphenyl) methyl acetate 5h

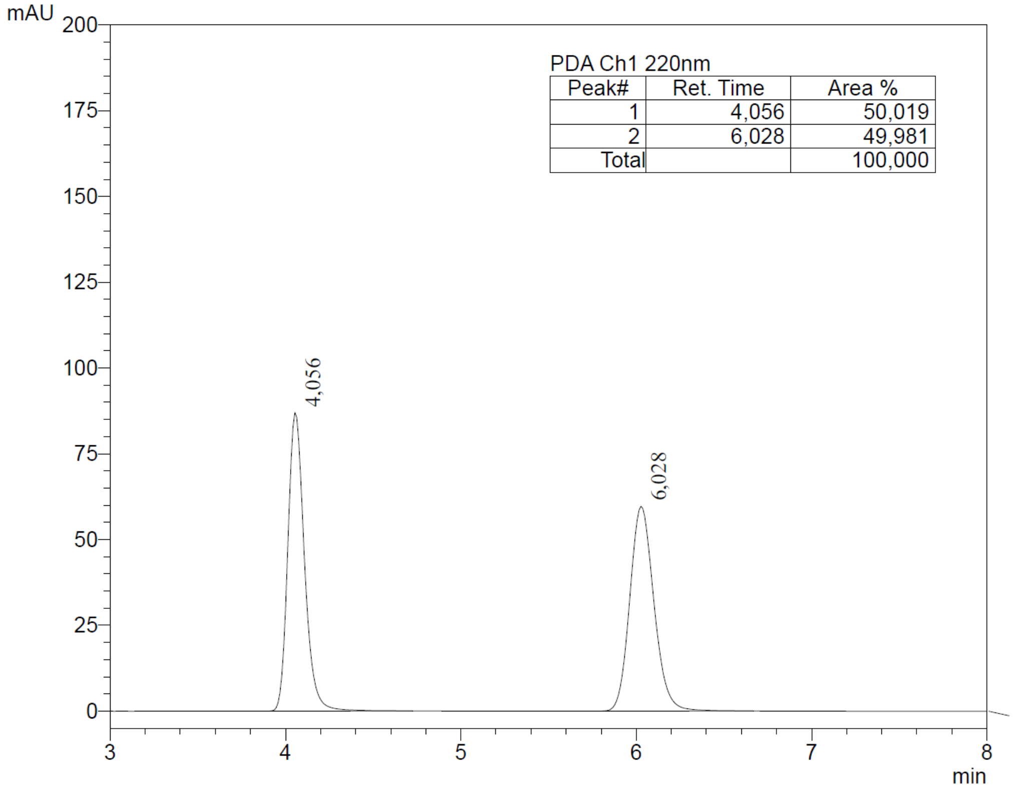

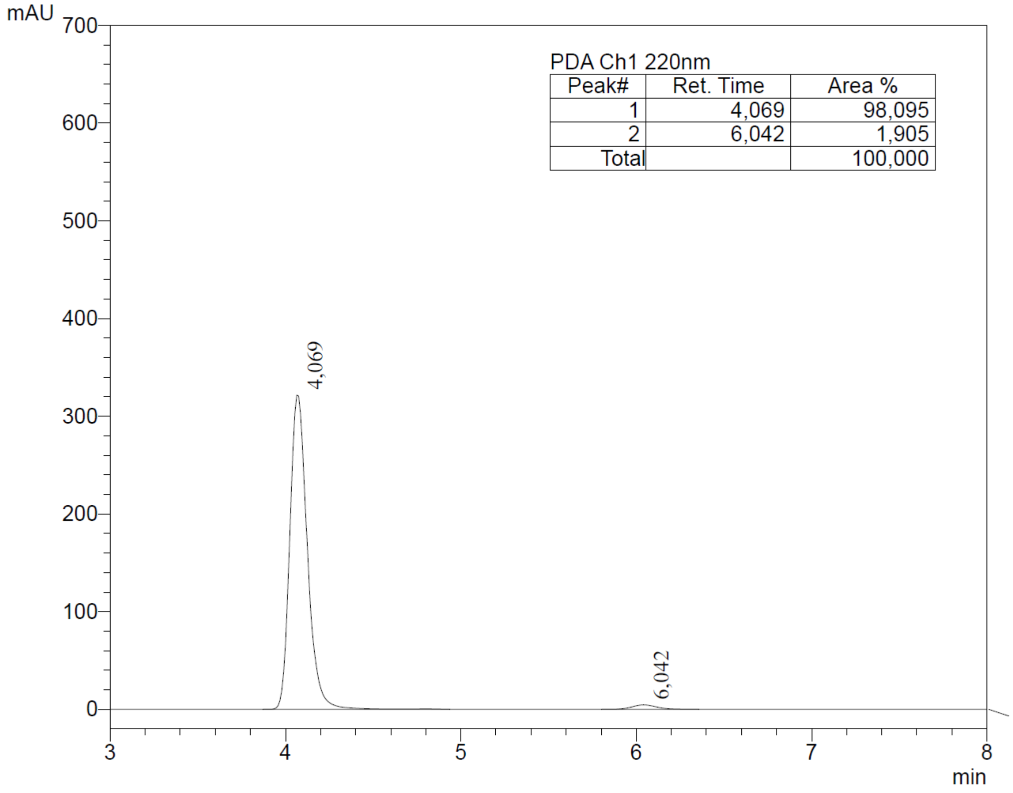

Supplementary Figure 27. HPLC spectra of (*S*)-cyano(3,5-dimethylphenyl)methyl acetate 5h. Diacel Chiralpak AS-3, *n*-heptane:isopropanol = 90:10, flow = 1.0 mL/min, 25 ℃, λ = 220 nm, t_R(major) = 4.0 min, t_R(minor) = 6.0 min, e.r. = 98:2.

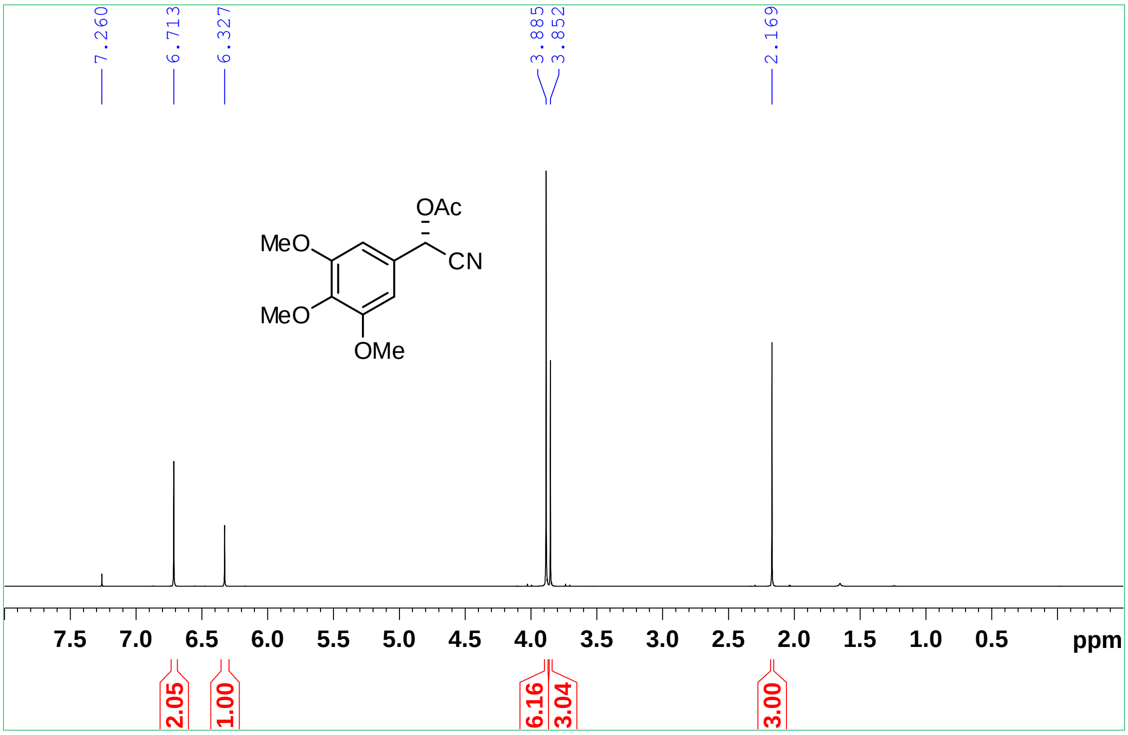

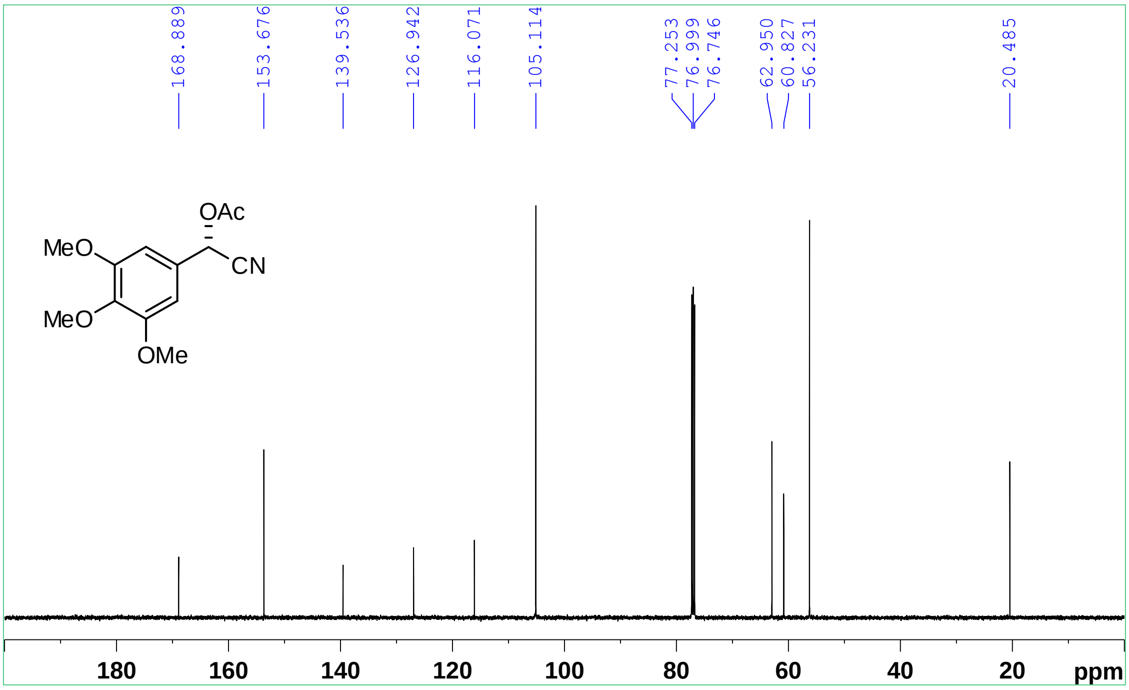

Supplementary Figure 28. ^{1}**H and** ^{13}**C NMR spectra of (*S*)-cyano(3,4,5-trimethoxyphenyl) methyl acetate 5i**

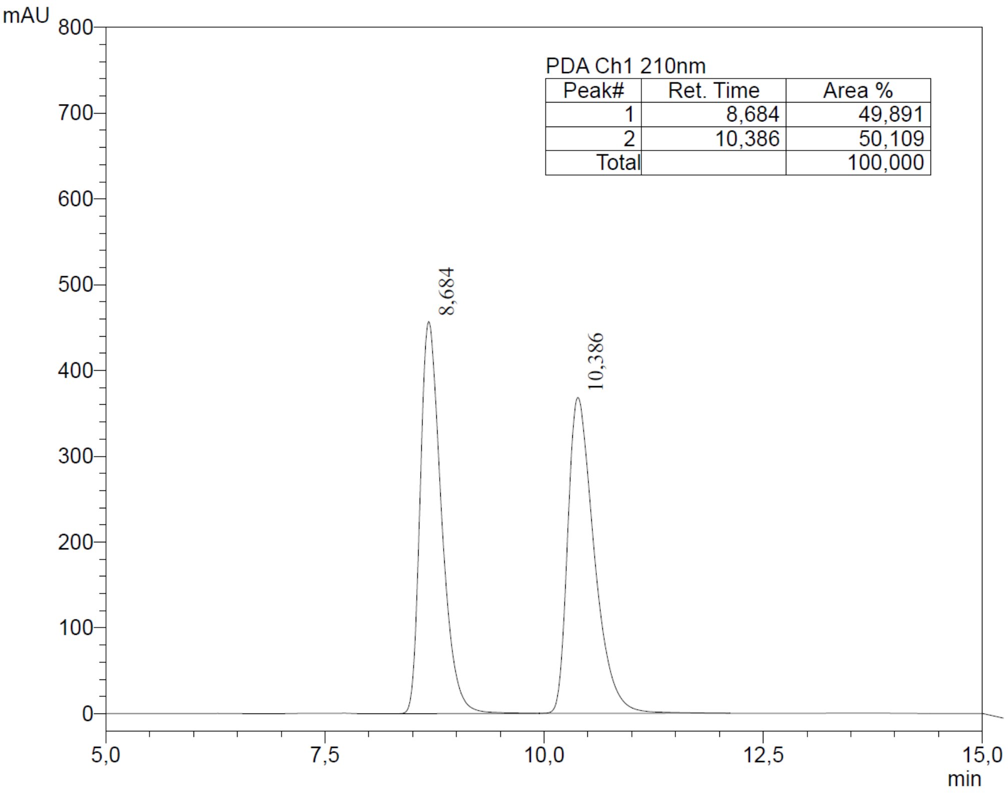

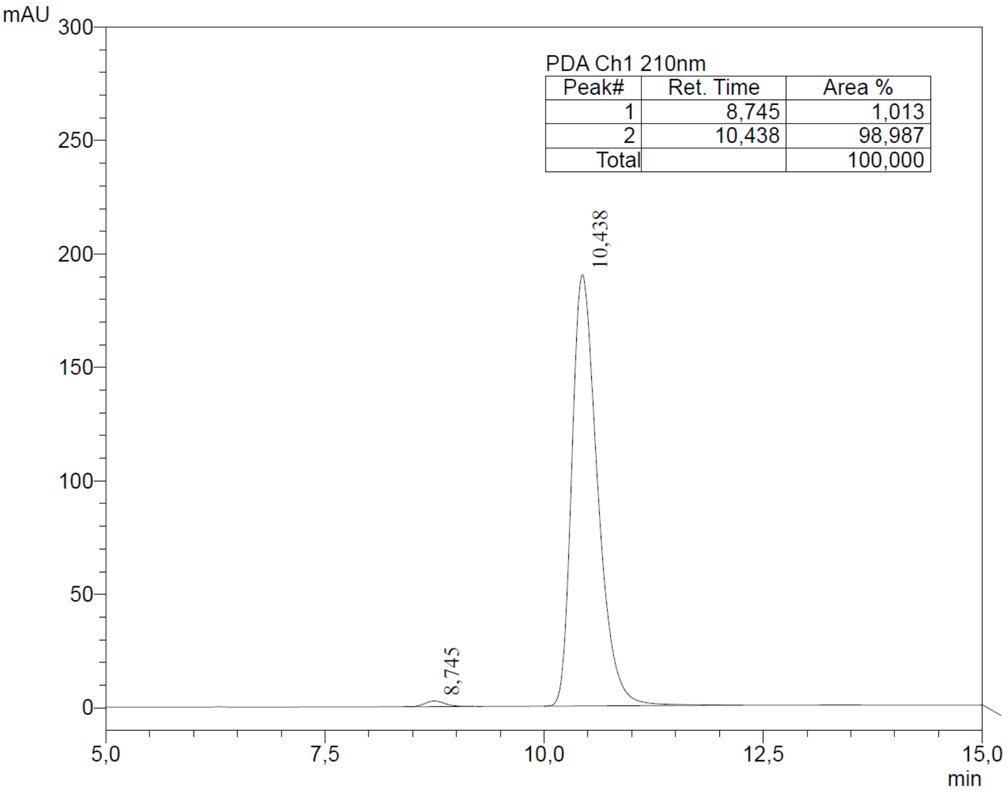

Supplementary Figure 29. HPLC spectra of (*S*)-cyano(3,4,5-trimethoxyphenyl)methyl acetate 5i. Diacel Chiralpak OD-3, *n*-heptane:isopropanol = 90:10, flow = 1.0 mL/min, 25 ℃, λ = 210 nm, t_R(minor) = 8.7 min, t_R(major) = 10.4 min, e.r. = 99:1.

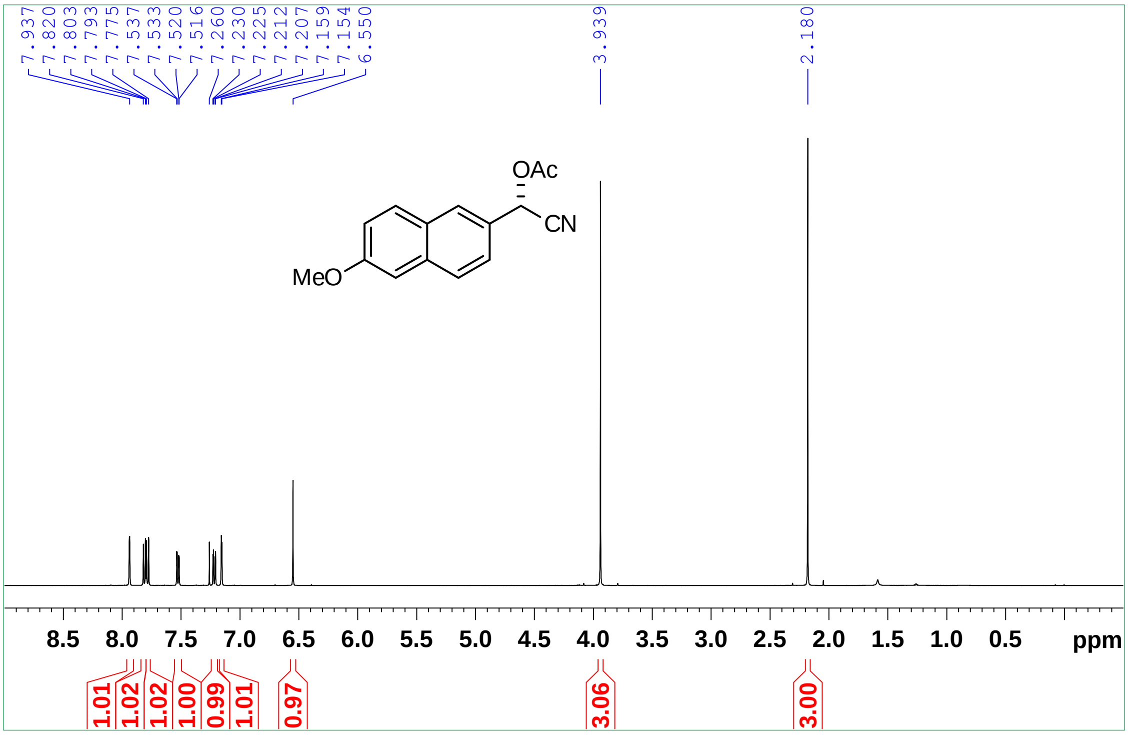

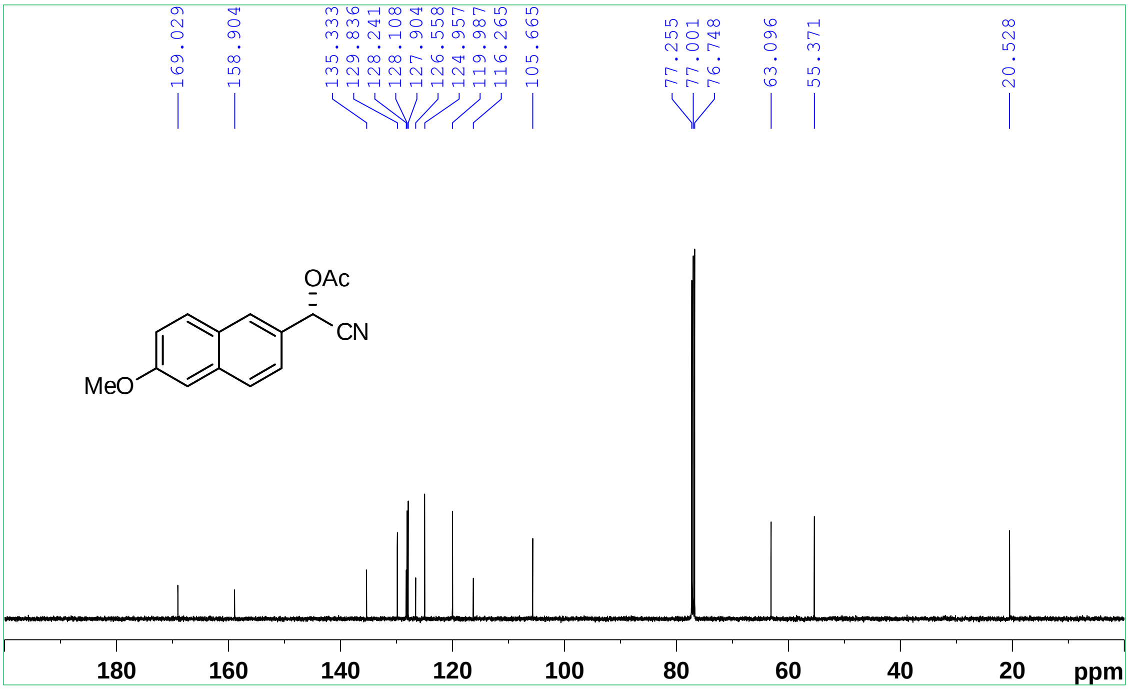

Supplementary Figure 30. ^{1}**H and** ^{13}**C NMR spectra of (*S*)-cyano(6-methoxynaphthalen-2-yl)methyl acetate 5j**

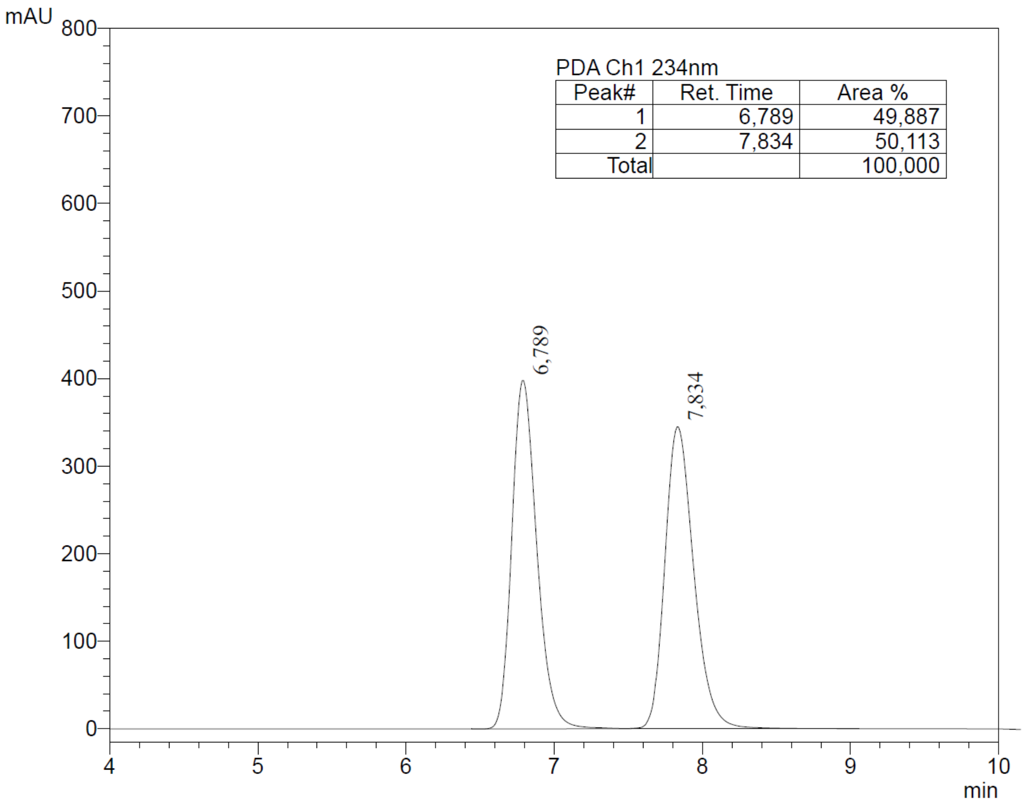

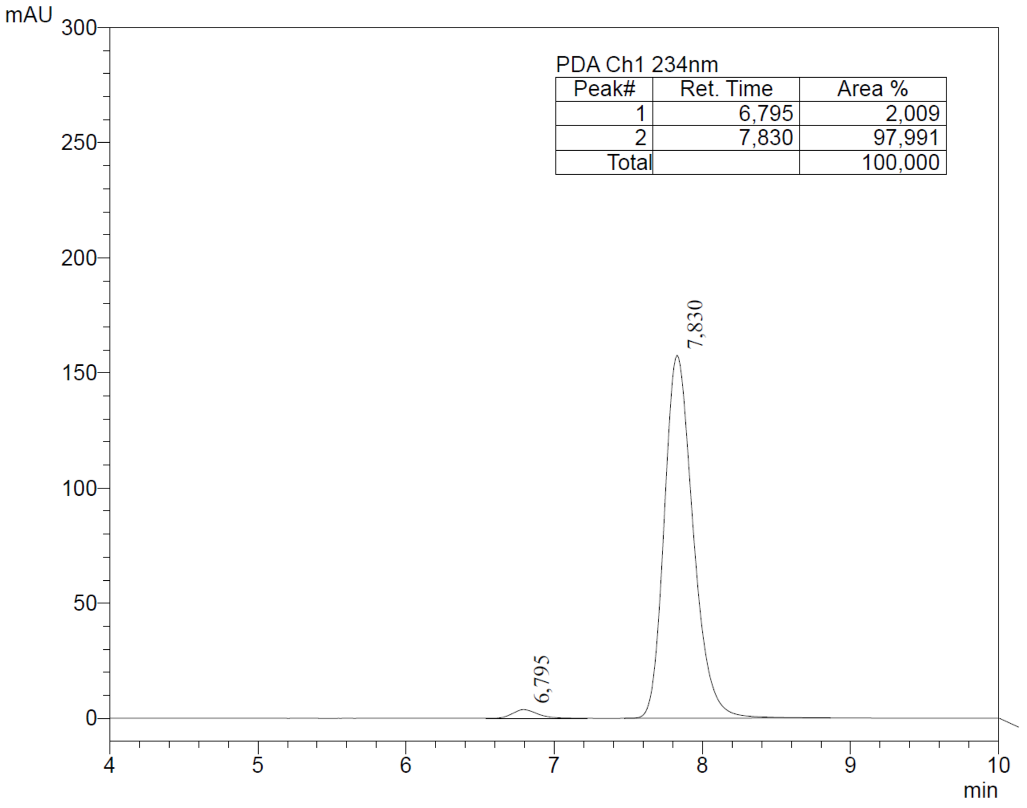

Supplementary Figure 31. HPLC spectra of (*S*)-cyano(6-methoxynaphthalen-2-yl)methyl acetate 5j. Diacel Chiralpak OD-3, *n*-heptane:isopropanol = 90:10, flow = 1.0 mL/min, 25 ℃, λ = 234 nm, t_R(minor) = 6.8 min, t_R(major) = 7.8 min, e.r. = 98:2.

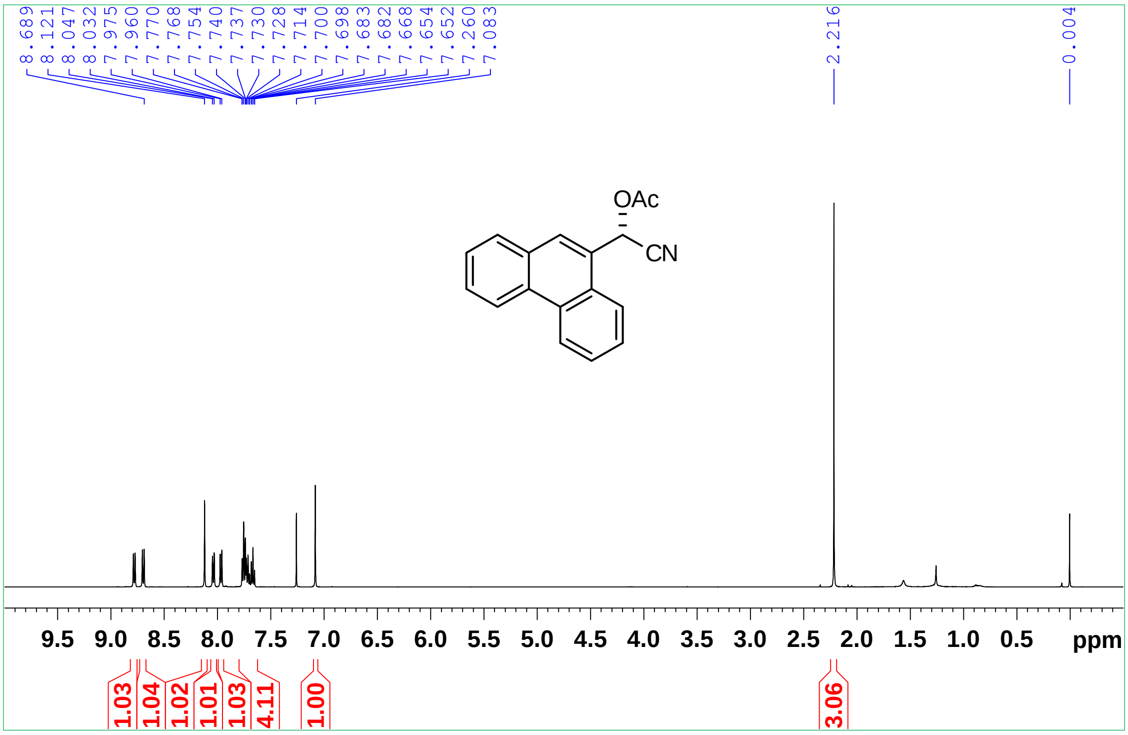

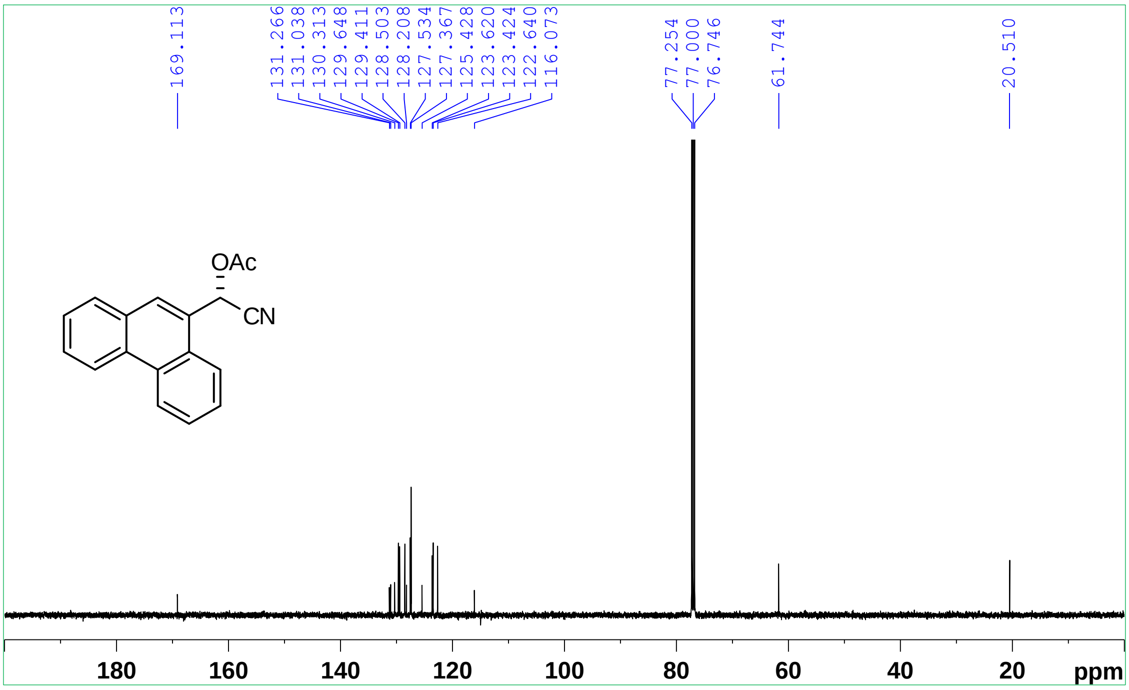

Supplementary Figure 32. ^{1}H and ^{13}C NMR spectra of (*S*)-cyano(phenanthren-9-yl)methyl acetate 5k

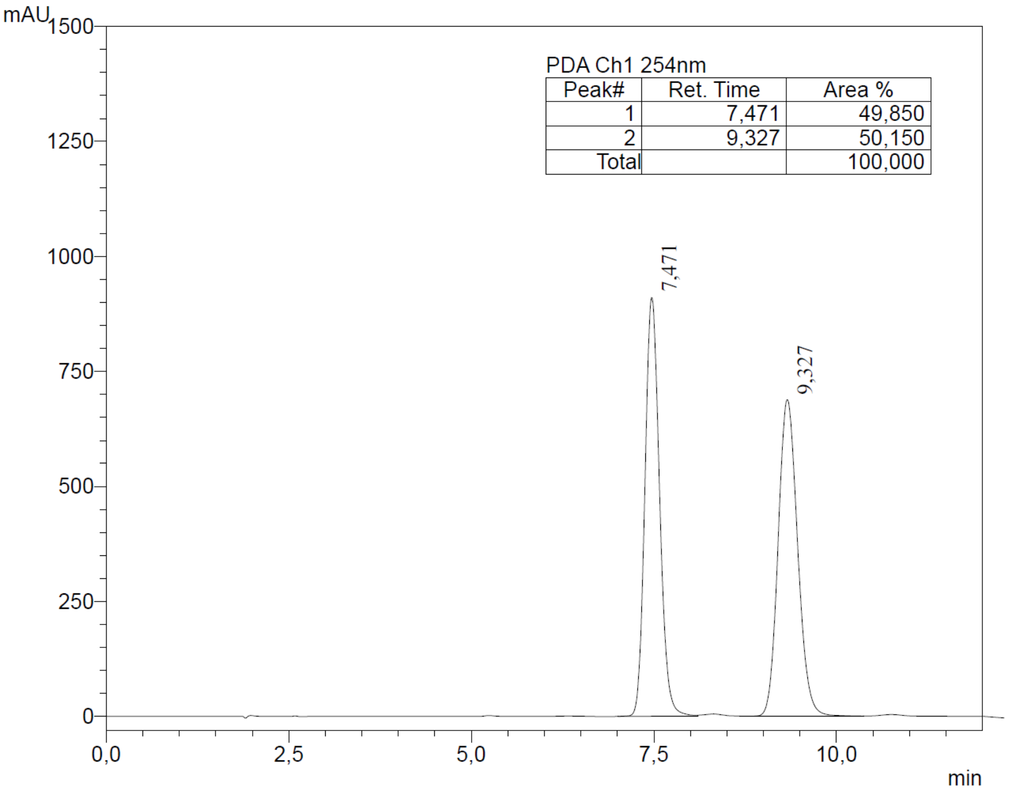

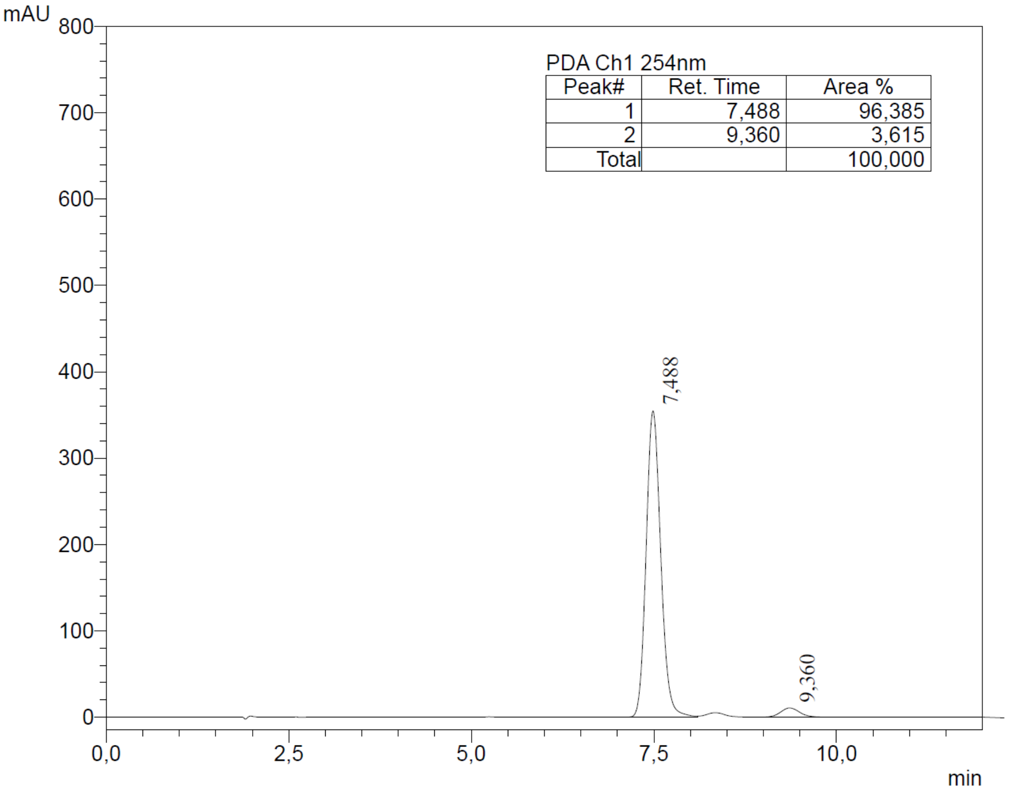

Supplementary Figure 33. HPLC spectra of (*S*)-cyano(phenanthren-9-yl)methyl acetate 5k. Diacel Chiralpak AS-3, *n*-heptane:isopropanol = 90:10, flow = 1.0 mL/min, 25 ℃, λ = 254 nm, t_R(major) = 7.5 min, t_R(minor) = 9.4 min, e.r. = 96.5:3.5.

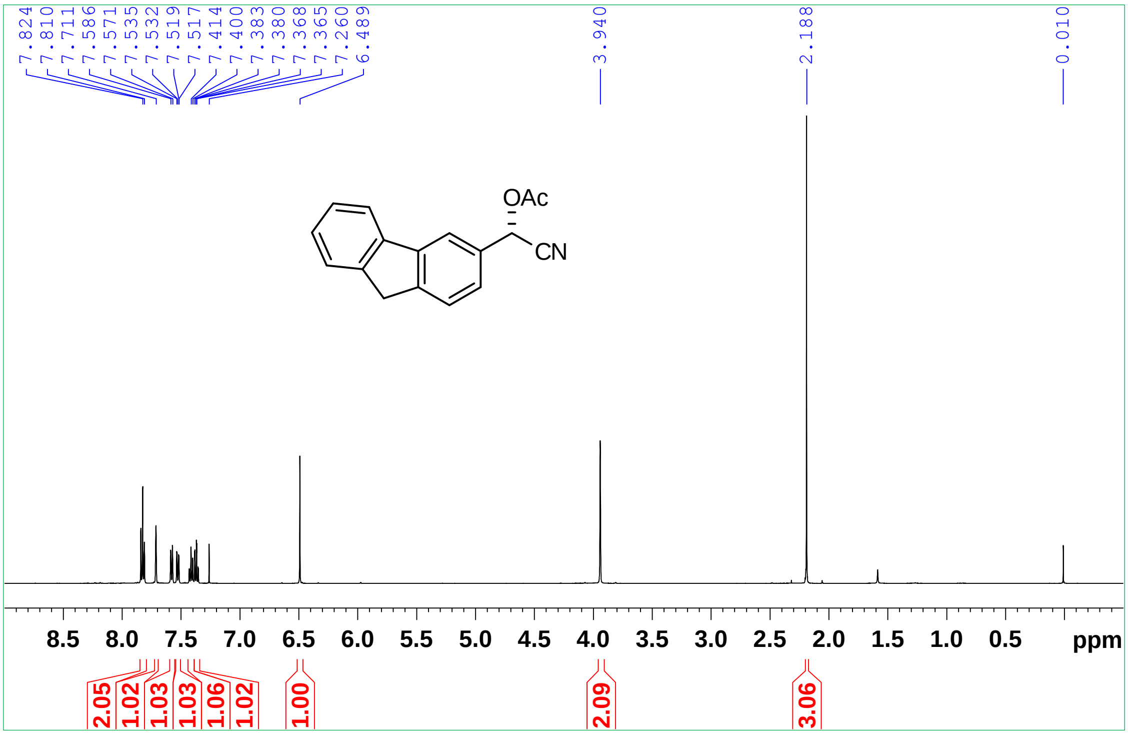

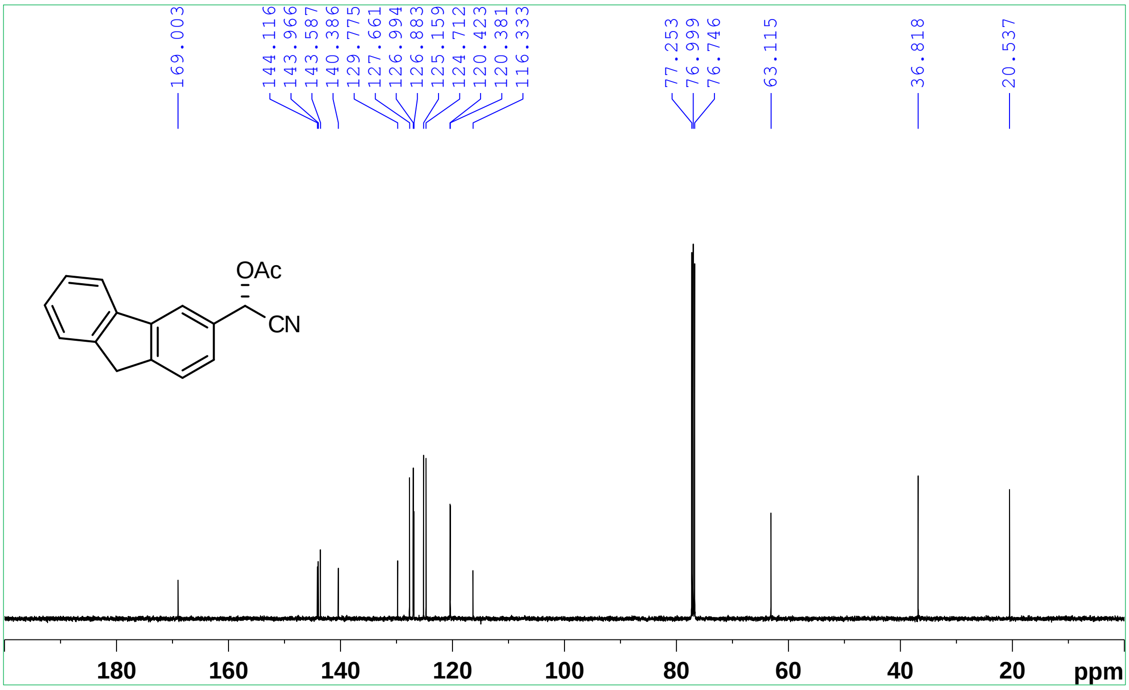

Supplementary Figure 34. **^{1}H and ^{13}C NMR spectra of (*S*)-cyano(9H-fluoren-3-yl)methyl acetate 5l**

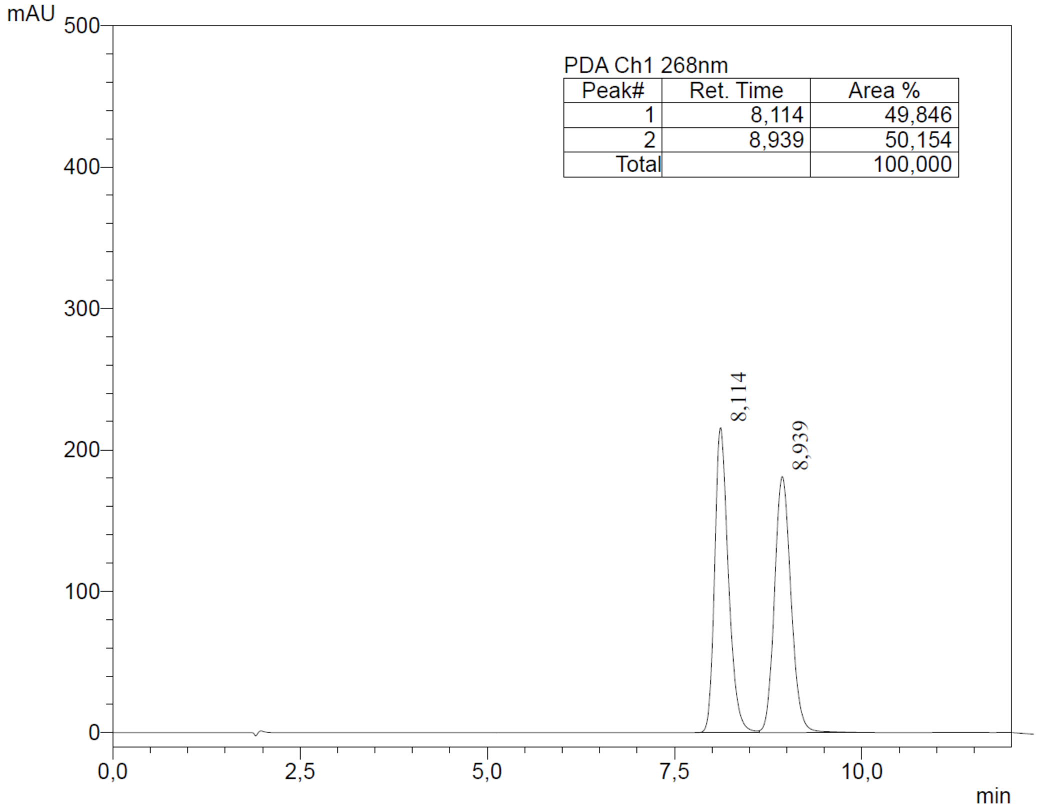

Peak#	Ret. Time	Area %
1	8,114	49,846
2	8,939	50,154
Total		100,000

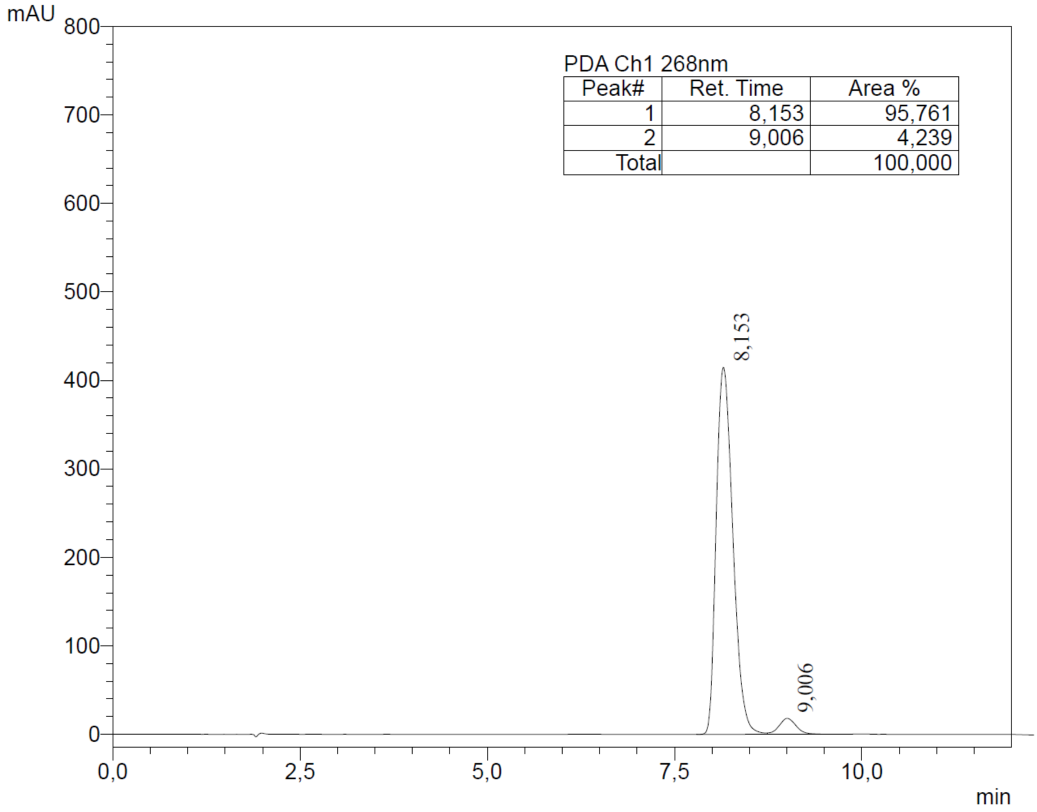

Peak#	Ret. Time	Area %
1	8,153	95,761
2	9,006	4,239
Total		100,000

Supplementary Figure 35. HPLC spectra of (*S*)-cyano(9H-fluoren-3-yl)methyl acetate 5l. Diacel Chiralpak AS-3, *n*-heptane:isopropanol = 90:10, flow = 1.0 mL/min, 25 ℃, λ = 268 nm, t_R(major) = 8.2 min, t_R(minor) = 9.0 min, e.r. = 96:4.

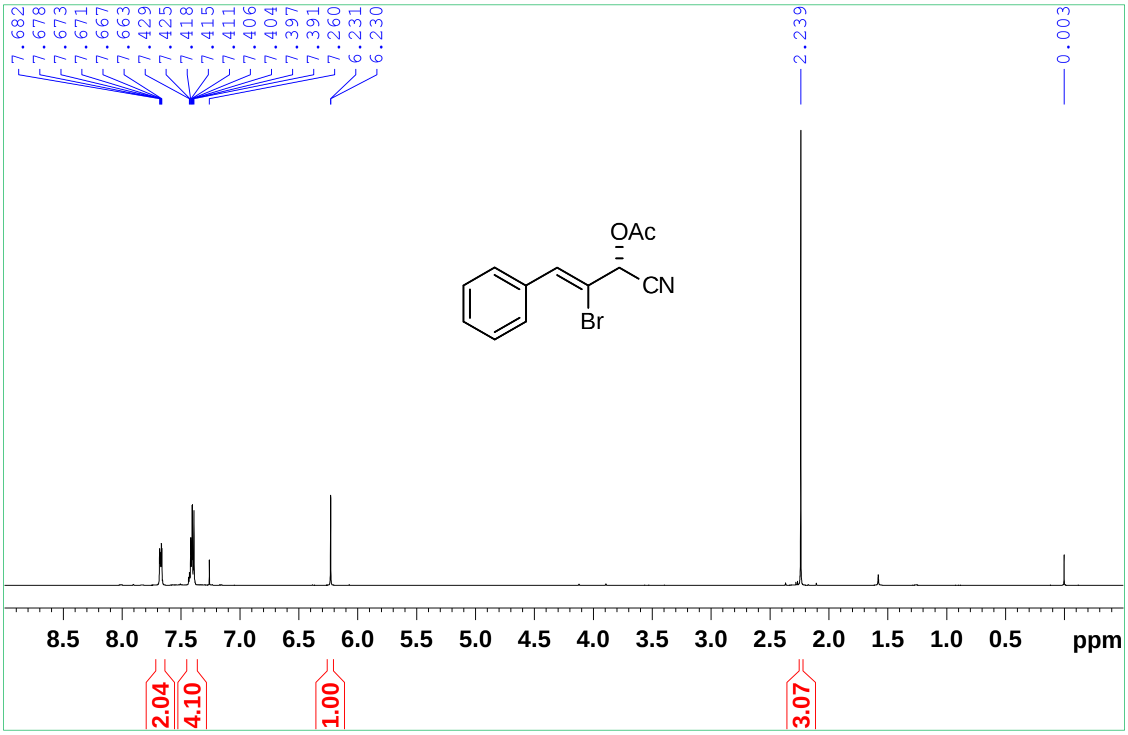

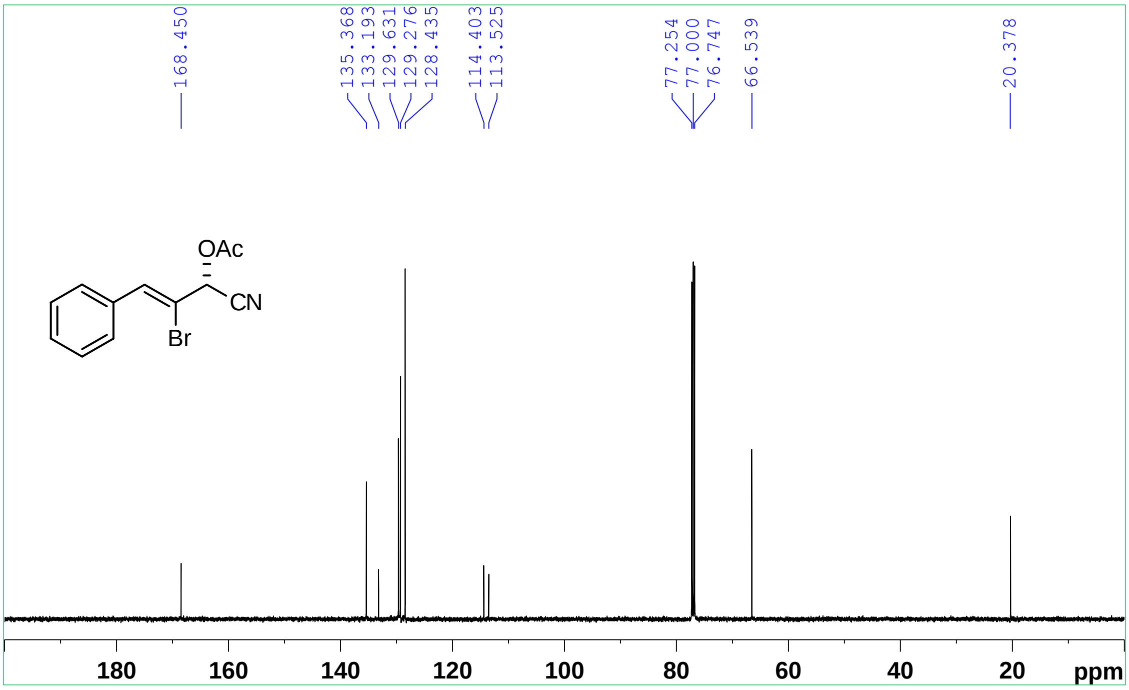

Supplementary Figure 36. **^{1}H and ^{13}C NMR spectra of (*R*, *Z*)-2-bromo-1-cyano-3-phenylallyl acetate 5m**

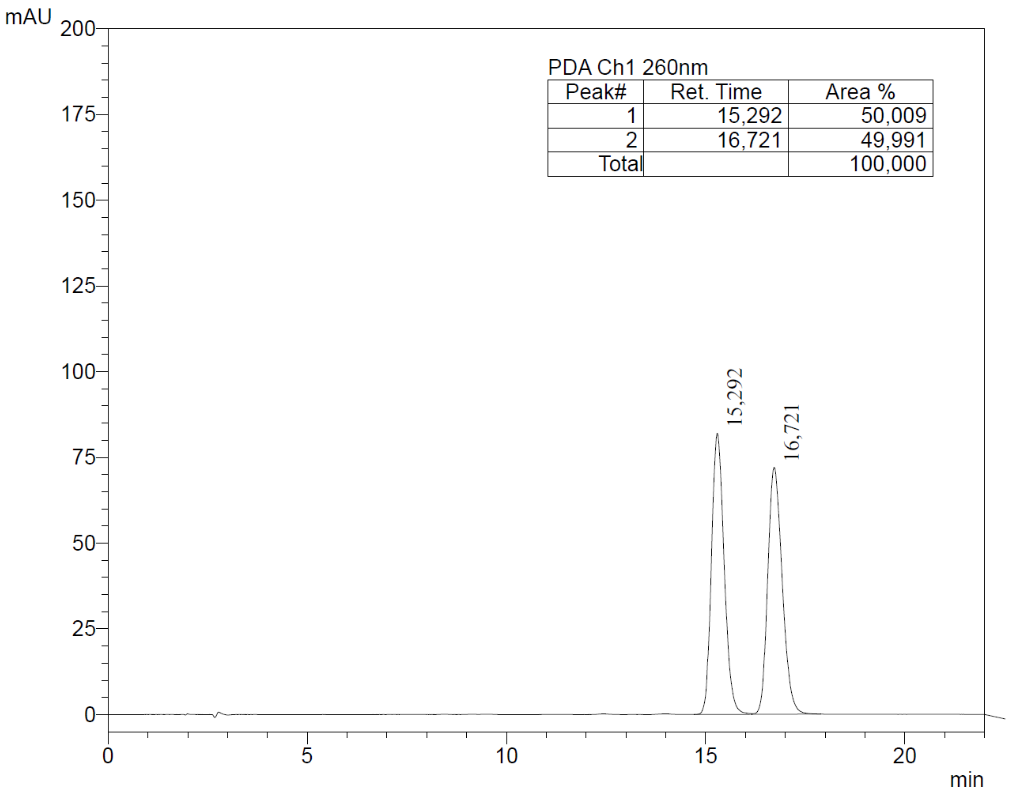

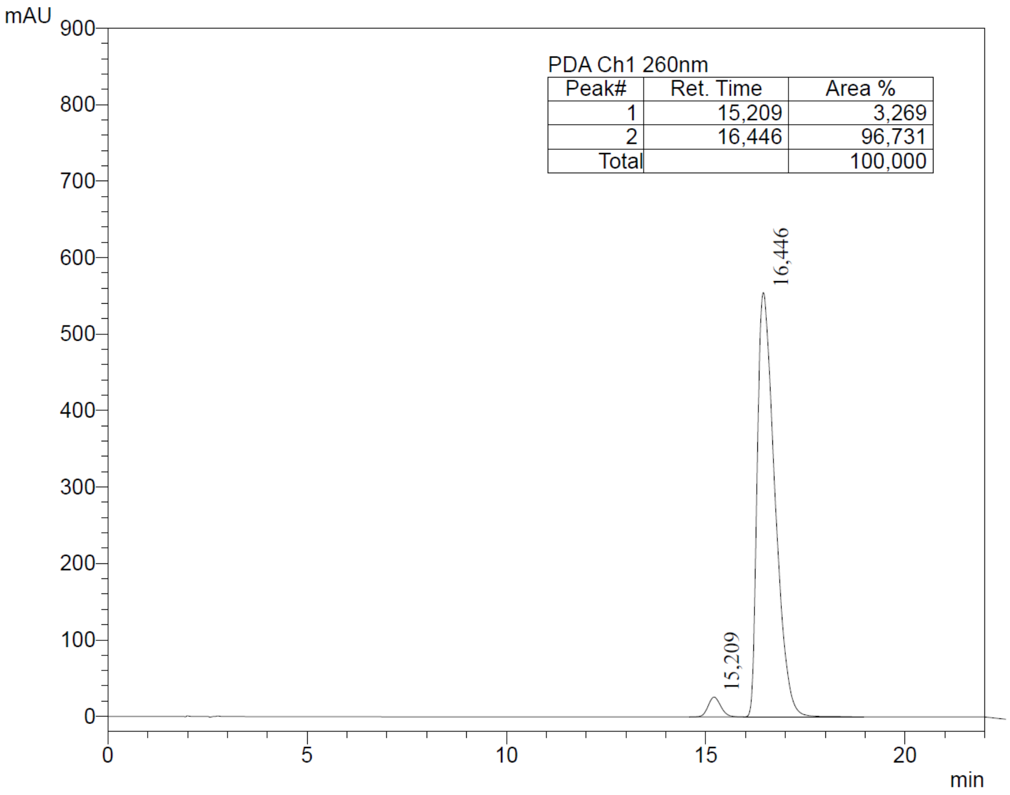

Supplementary Figure 37. HPLC spectra of (*R*, *Z*)-2-bromo-1-cyano-3-phenylallyl acetate 5m. Diacel Chiralpak AS-3, *n*-heptane:isopropanol = 99:1, flow = 1.0 mL/min, 25 ℃, λ = 260 nm, t_R(minor) = 15.2 min, t_R(major) = 16.4 min, e.r. = 96.5:3.5.

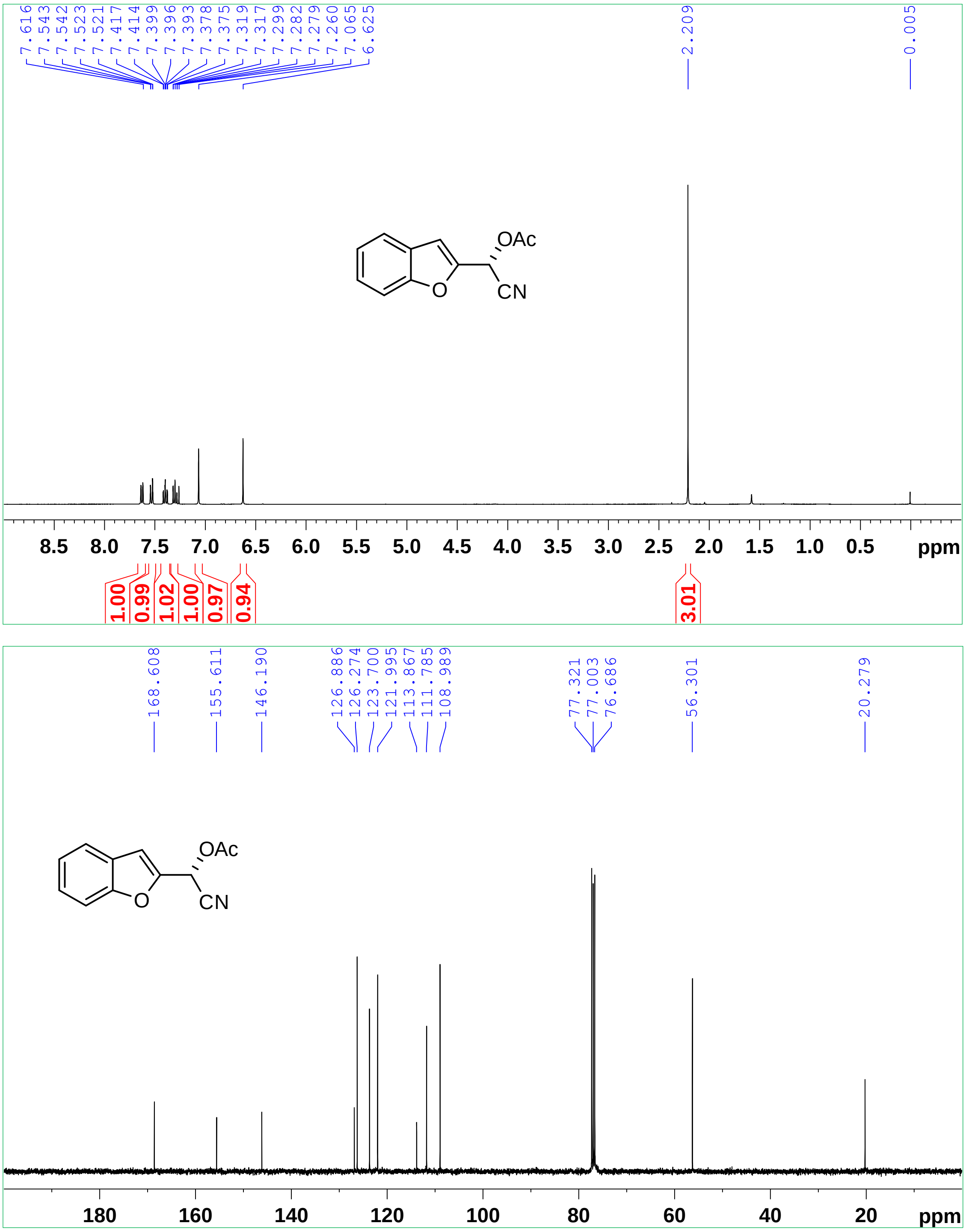

Supplementary Figure 38. ^{1}**H and** ^{13}**C NMR spectra of (*R*)-benzofuran-2-yl(cyano) methyl acetate 5n**

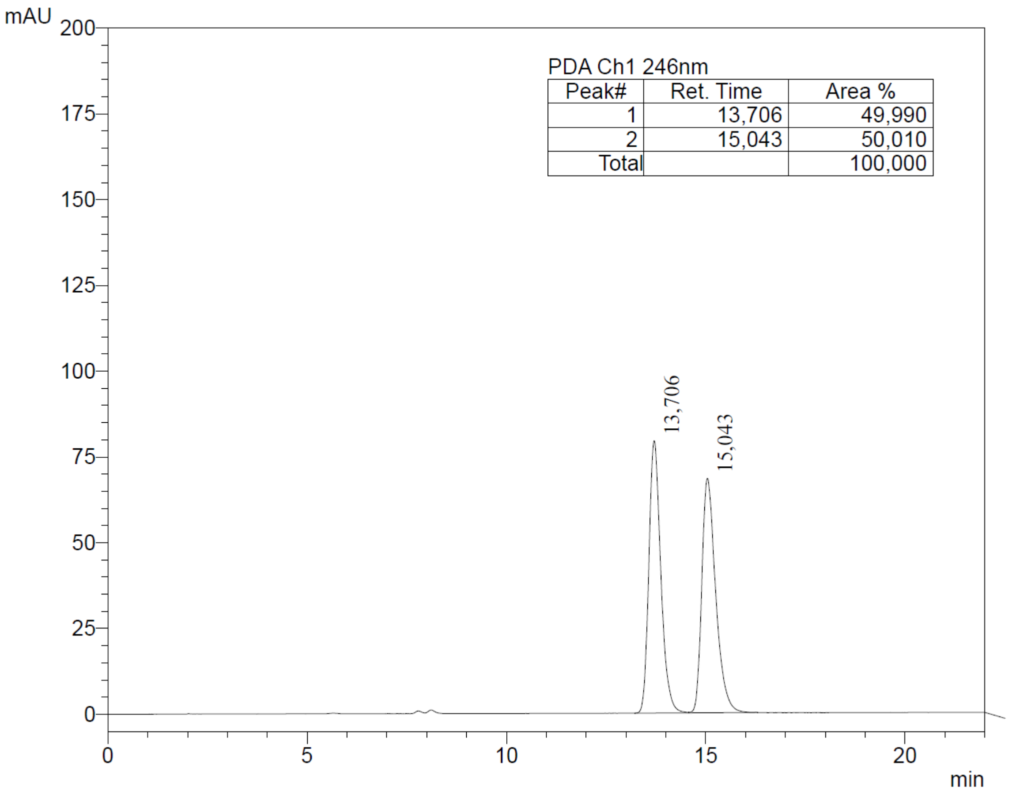

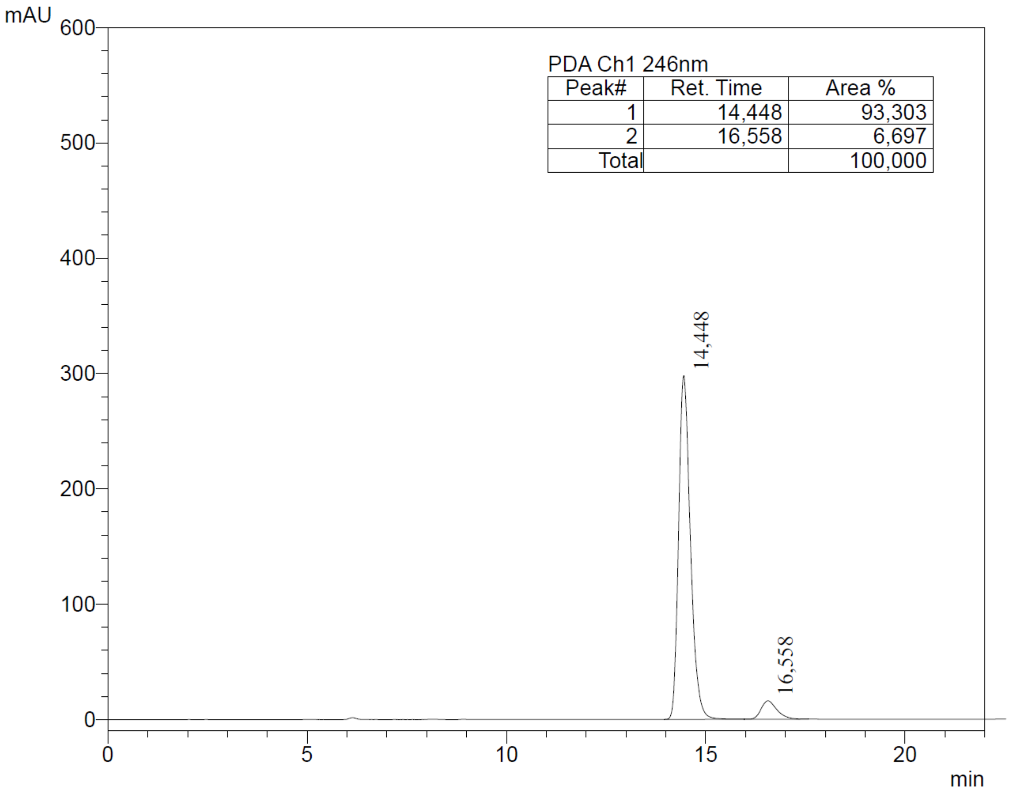

Supplementary Figure 39. HPLC spectra of (*R*)-benzofuran-2-yl(cyano)methyl acetate 5n. Diacel Chiralpak OD-3, *n*-heptane:isopropanol = 99.5:0.5, flow = 1.0 mL/min, 25 ℃, λ = 246 nm, t_R(major) = 14.4 min, t_R(minor) = 16.6 min, e.r. = 93.5:6.5.

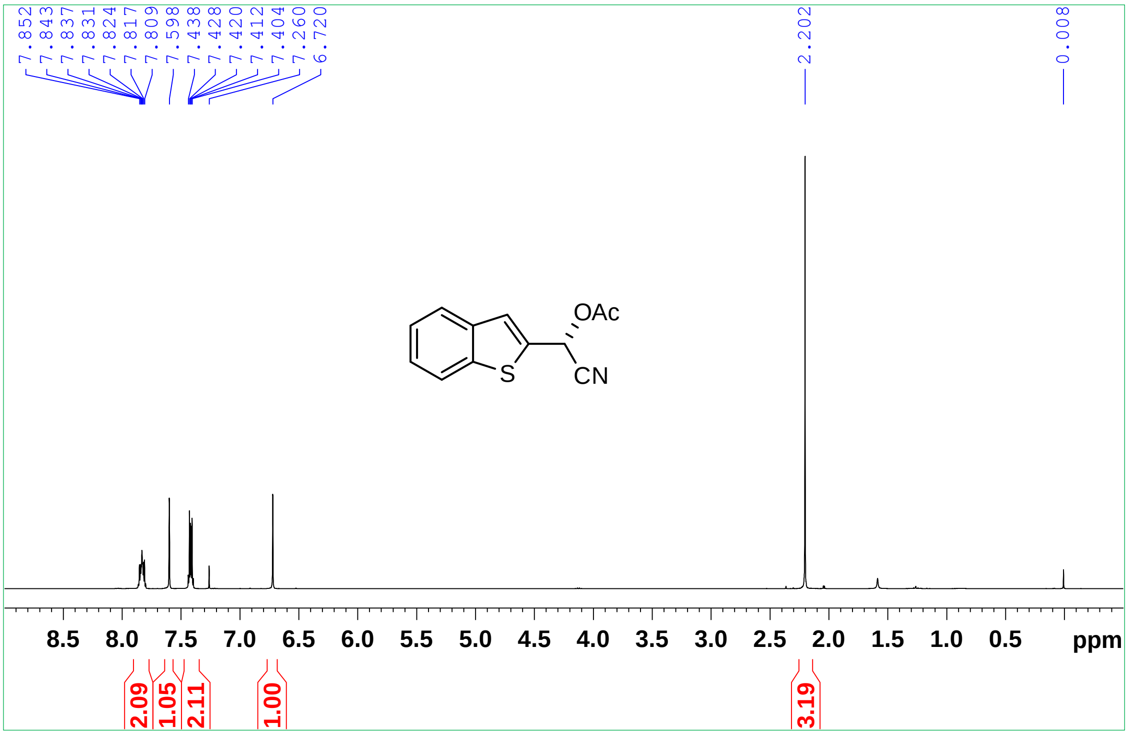

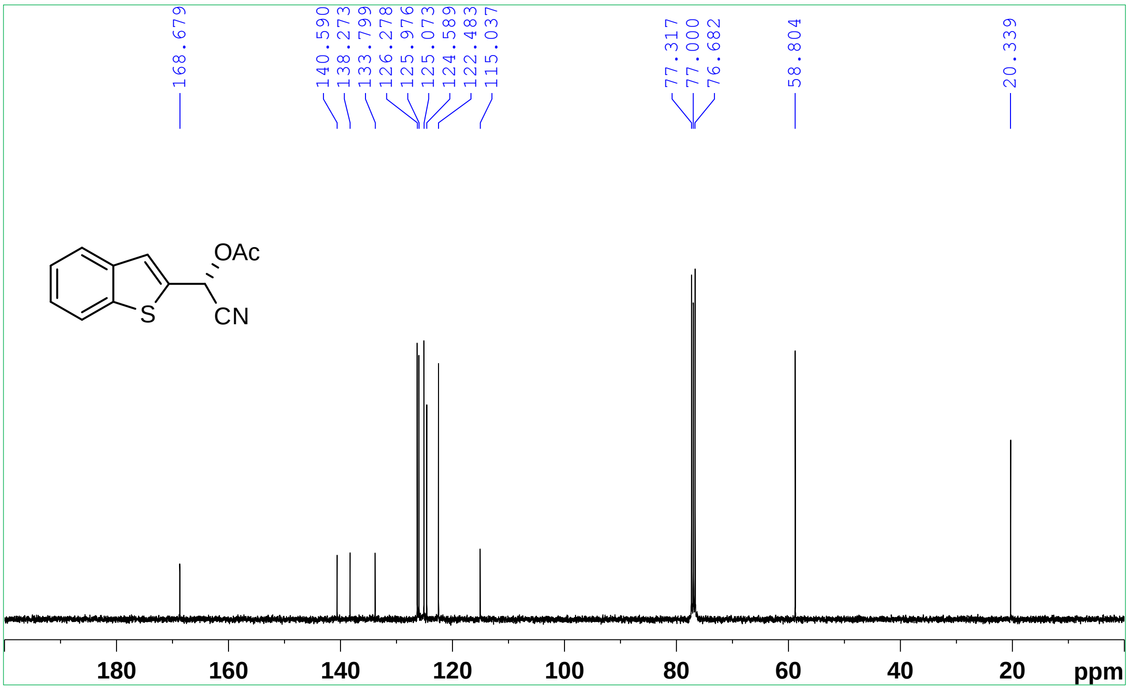

Supplementary Figure 40. **^{1}H and ^{13}C NMR spectra of (*R*)-benzo[*b*]thiophen-2-yl(cyano) methyl acetate 5o**

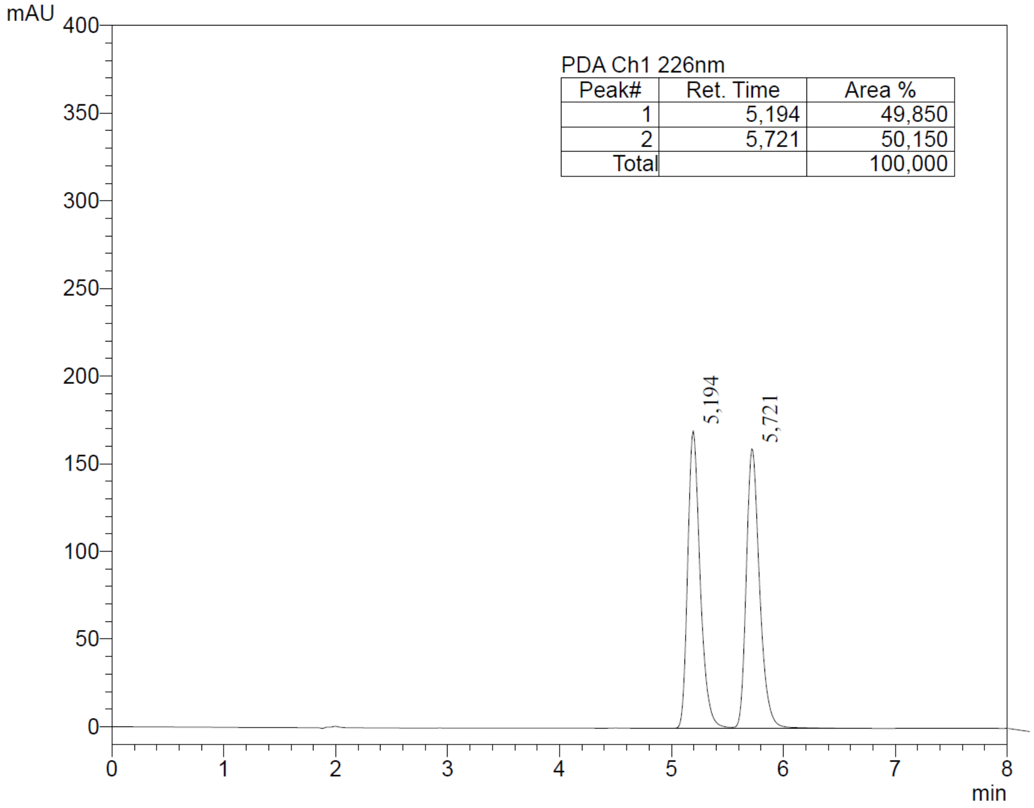

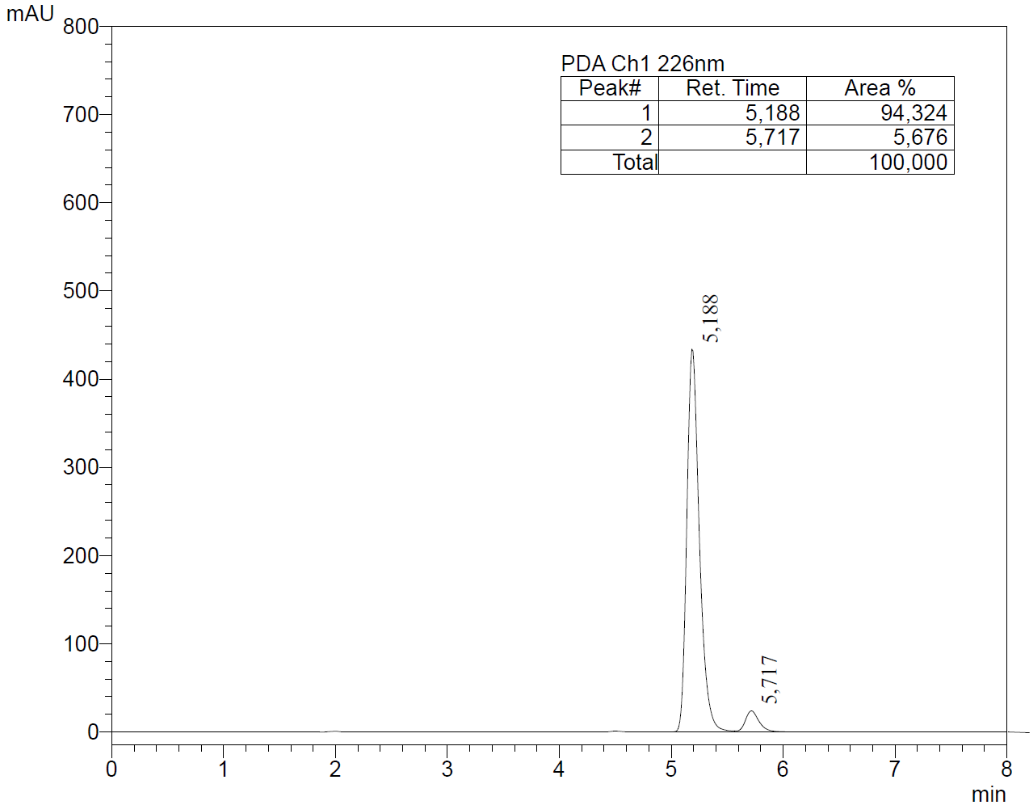

Supplementary Figure 41. HPLC spectra of (*R*)-benzo[*b*]thiophen-2-yl(cyano)methyl acetate 5o. Diacel Chiralpak AD-3, *n*-heptane:isopropanol = 90:10, flow = 1.0 mL/min, 25 ℃, λ = 226 nm, t_R(major) = 5.2 min, t_R(minor) = 5.7 min, e.r. = 94.5:5.5.

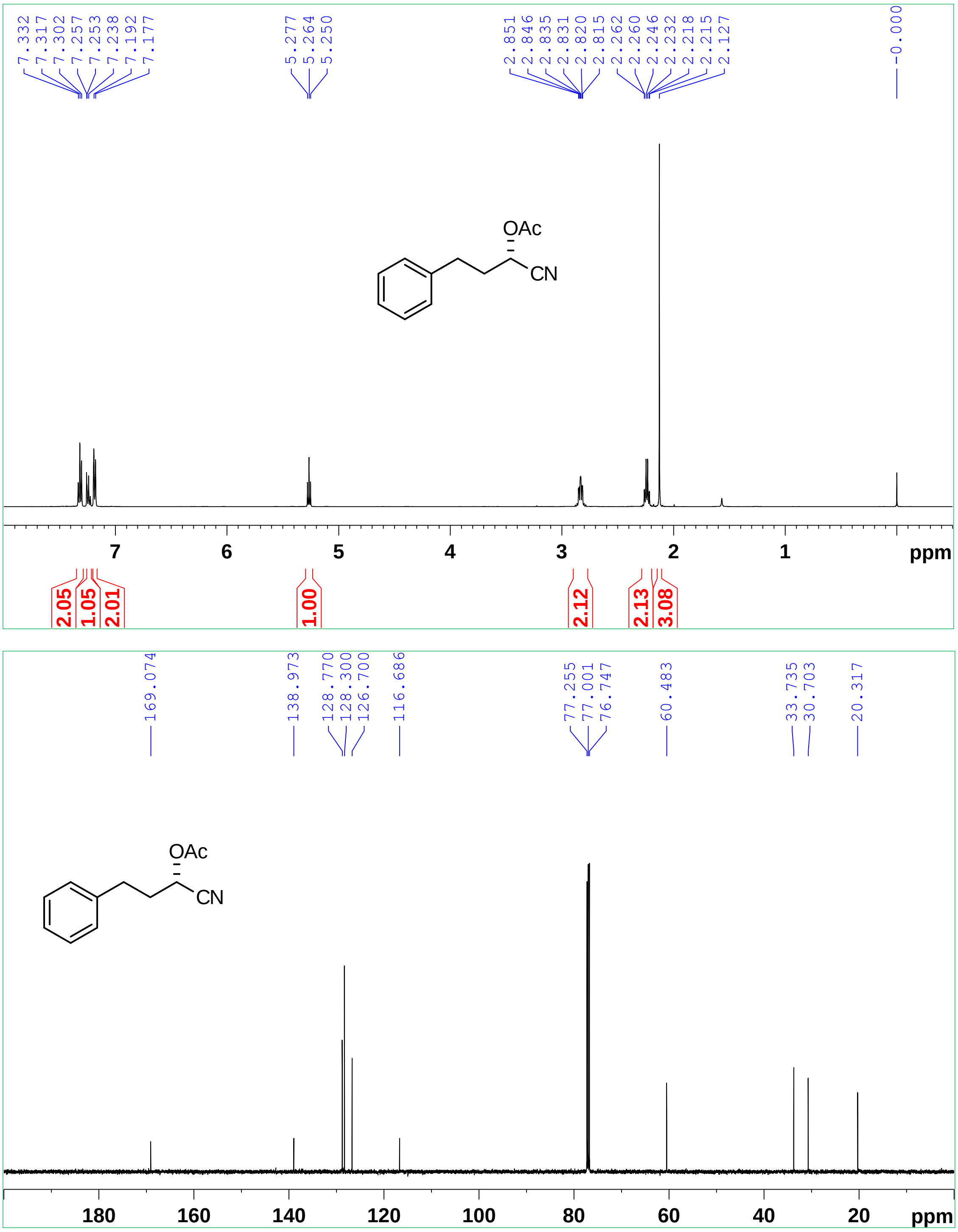

Supplementary Figure 42. ^{1}**H and** ^{13}**C NMR spectra of (*S*)-1-cyano-3-phenylpropyl acetate 5p**

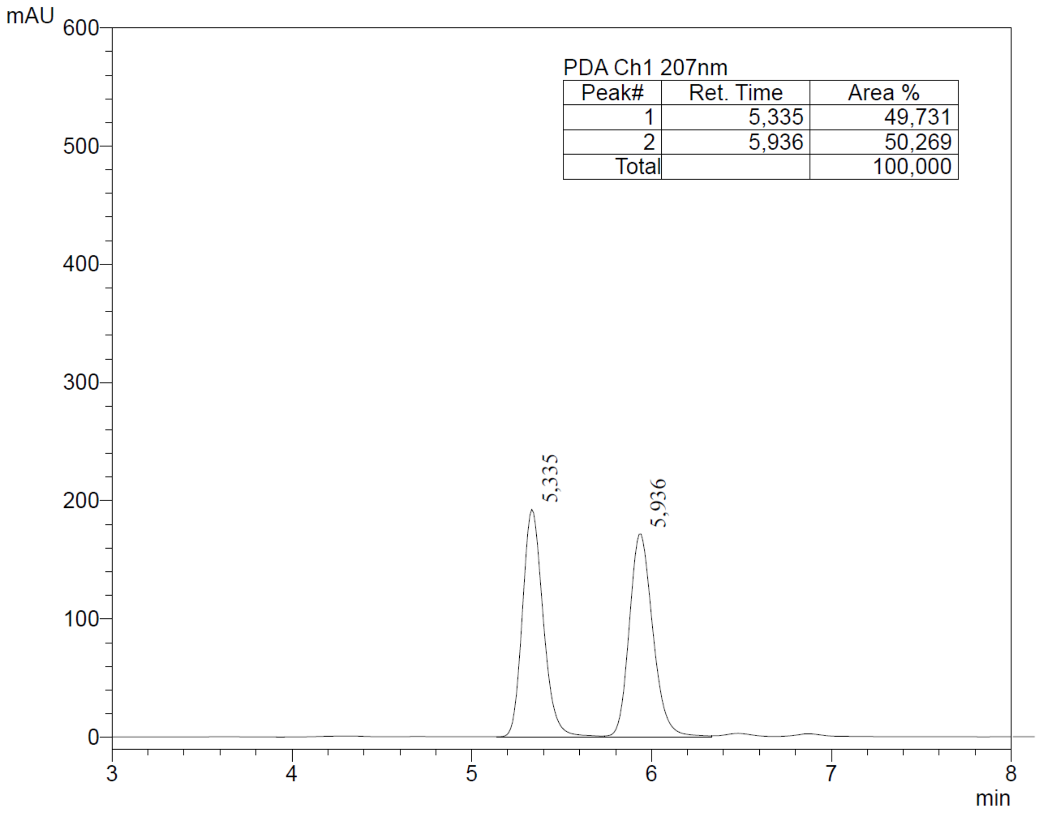

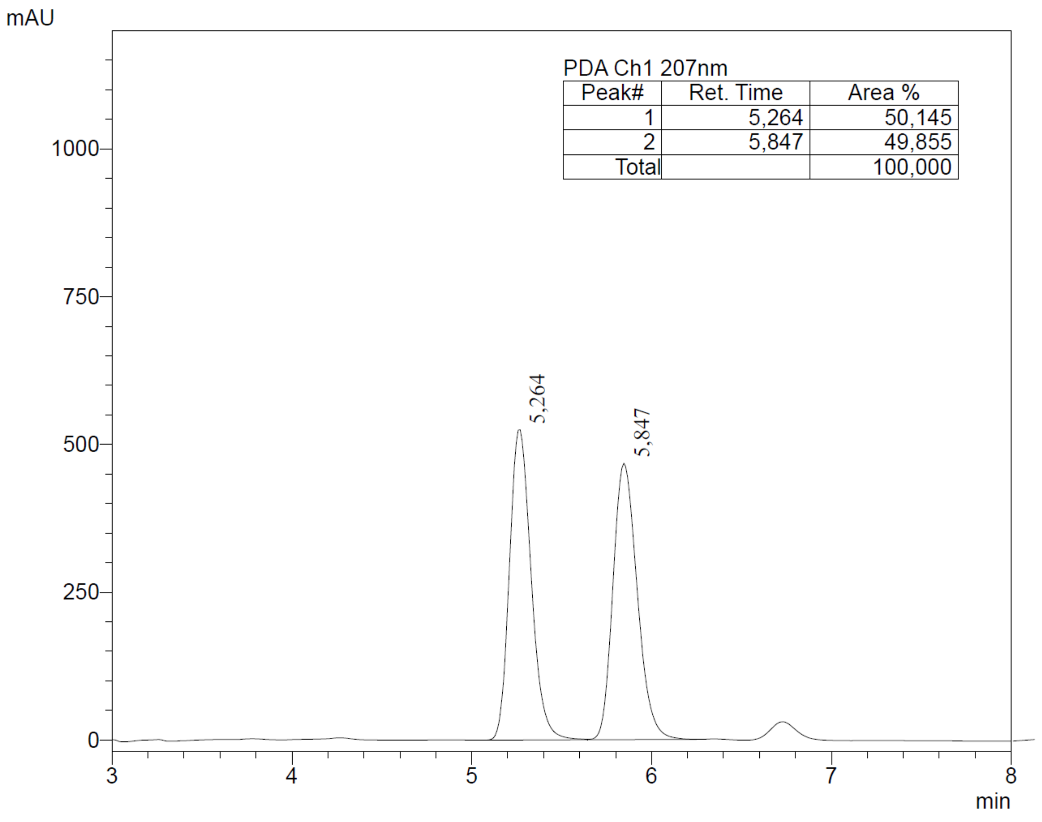

Supplementary Figure 43. HPLC spectra of (*S*)-1-cyano-3-phenylpropyl acetate 5p. Diacel Chiralpak AS-3, *n*-heptane:isopropanol = 90:10, flow = 1.0 mL/min, 25 ℃, λ = 207 nm, t_R(major) = 5.3 min, t_R(minor) = 5.8 min, e.r. = 50:50.

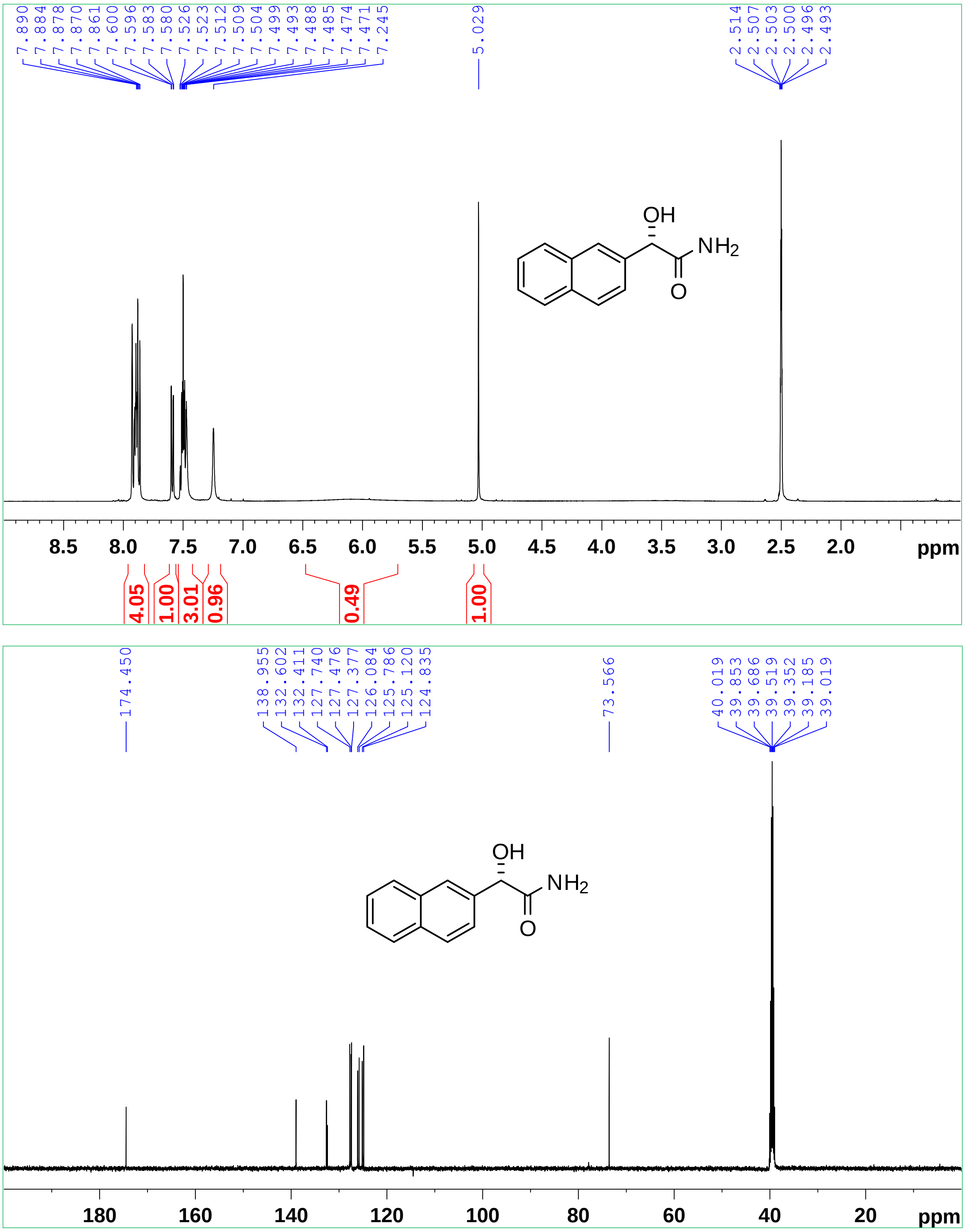

Supplementary Figure 44. ^{1}H and ^{13}C NMR spectra of (*S*)-2-hydroxy-2-(naphthalen-2-yl)acetamide 6

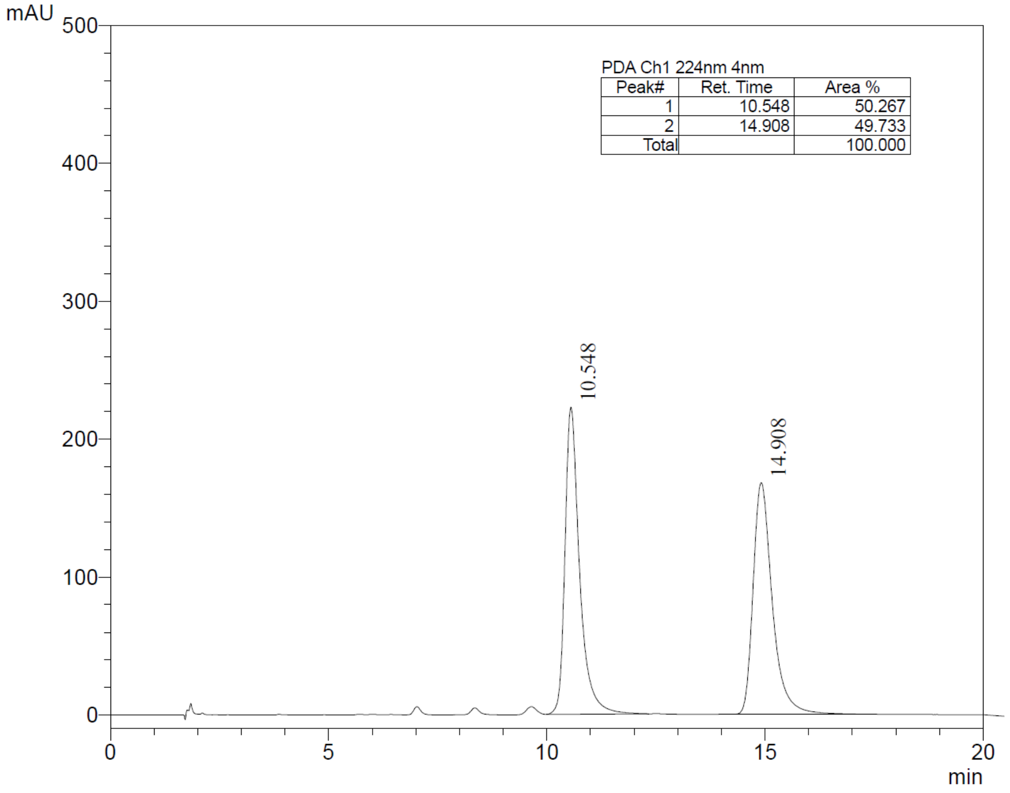

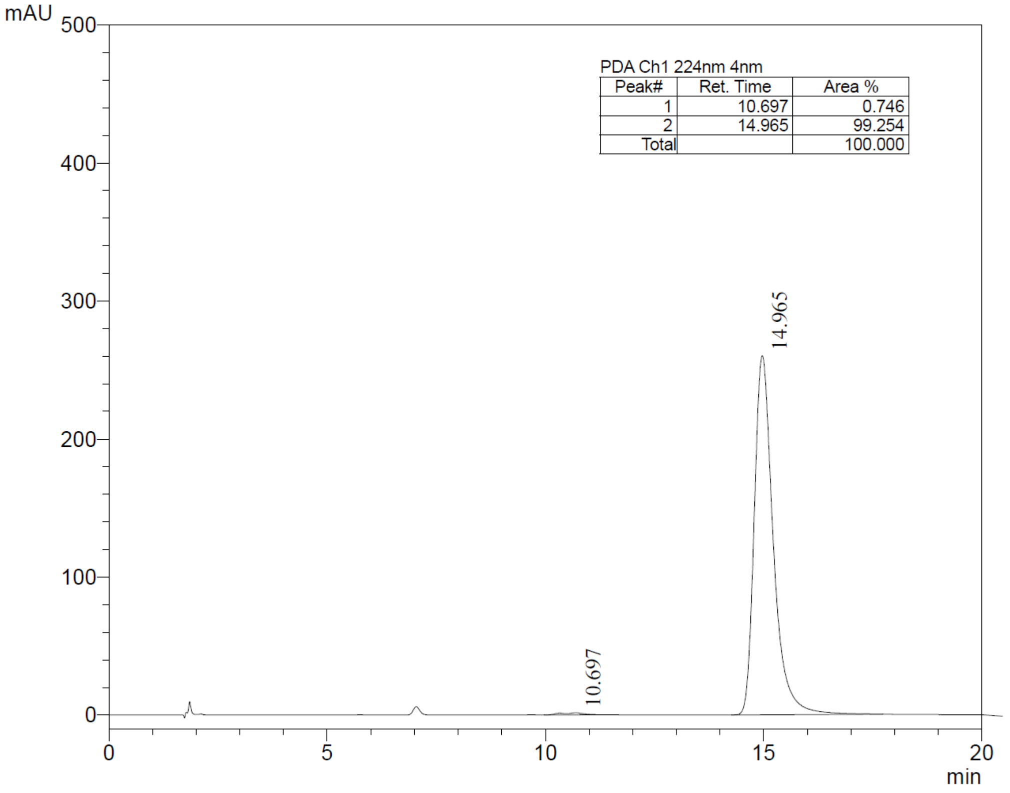

Supplementary Figure 45. HPLC spectra of (*S*)-2-hydroxy-2-(naphthalen-2-yl)acetamide 6. Diacel Chiralpak AD-3, *n*-heptane:isopropanol = 90:10, flow = 1.0 mL/min, 25 ℃, λ = 224 nm, t_R(minor) = 10.7 min, t_R(major) = 15.0 min, e.r. = 99.2:0.8.

Supplementary Tables

Supplementary Table 1. Optimization of Reaction Conditions - Solvent Screening[a].

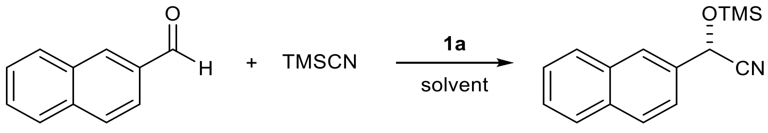

Entry	Solvent	Temp. (℃)	Time	Yield (%)[b]	e.r.[c]
1	**Et_2O**	-30	6 d	98	**92 : 8**
2	THF	-30	6 d	64	84.5 : 15.5
3	$(TMS)_2O$	-30	6 d	96	83.5 : 16.5
4	CH_2Cl_2	-30	6 d	95	61 : 39
5	DME	-30	6 d	50	86 : 14
6	hexane	-30	6 d	97	85 : 15
7	EtOAc	-30	6 d	96	87.5 : 12.5
8	1,4-dioxane	r.t.	40 h	96	67 : 33
9	toluene	r.t.	2 d	97	83 : 17

[a] Reactions were performed with aldehyde **2a** (0.05 mmol, 0.16 M), TMSCN (0.1 mmol), and catalyst **1a** (3 mol%) in solvent. [b] Isolated yields of the corresponding acetate **5**. [c] e.r. values of corresponding acetate **5** were determined by HPLC analysis using a chiral stationary phase.

Supplementary Table 2. Ratios and chemical shifts of silylated disulfonimides with different silylating agents.

Entry	Silylating agent	Disulfonimide	Ratio of *N*- and *O*-silylated species[a]	δ ^{29}Si (ppm)	
				N-silylated species	*O*-silylated species
1	**7a**	**1a**	1:3	24.89	40.38
2	**7b**	**1a**	1:10	28.25	42.49
3	**7a**	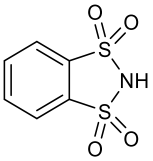	>99:1	21.81	-
4	**7b**	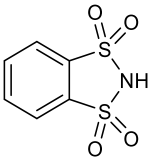	1:2	28.95	43.86
5	$(TMS)_2NH$	$(CH_3SO_2)_2NH$	-	22.61	39.72

The trends for the change in ratio between *N*- and *O*-silylated species going from TMS to TBS are in agreement with the literature[1,2].

Supplementary Discussion

Discussion concerning “Induction period” vs. "Dormant period"

According to IUPAC, “The initial slow phase of a chemical reaction which later accelerates” is called “induction period” [3]. “Slow” means that the reaction rate during this phase is different from zero. A typical “induction period” features a slower conversion to product but product formation is observed from the beginning onwards with an increasing rate.

In some catalytic reactions, a pre-catalyst needs to undergo a transformation to form the active catalyst, before the catalyst can take effect. Time is required for this transformation, hence the “induction period” (the progress as shown in the **red** rectangle of scheme below).

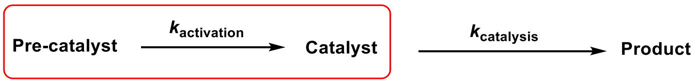

In principle, any reaction initiated by a pre-catalyst, should feature an "induction period", whether it is detectable or not. In such a typical case, once the pre-catalyst has been transformed into the active catalyst, it is stable during the reaction condition and immediately starts catalyzing the reaction. However, a different scenario operates when a reaction that features an "induction period" is accompanied with a quenching process as shown in the **green** rectangle of the scheme below.

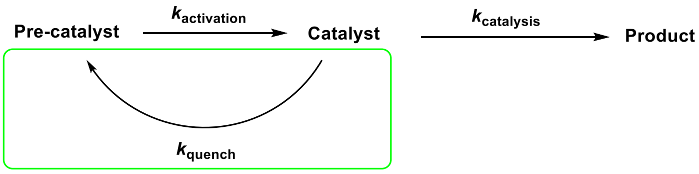

For the cyanosilylation of aldehydes catalyzed by disulfonimides **1**, there are two stages before the reaction to the desired product starts (as shown in the scheme below).

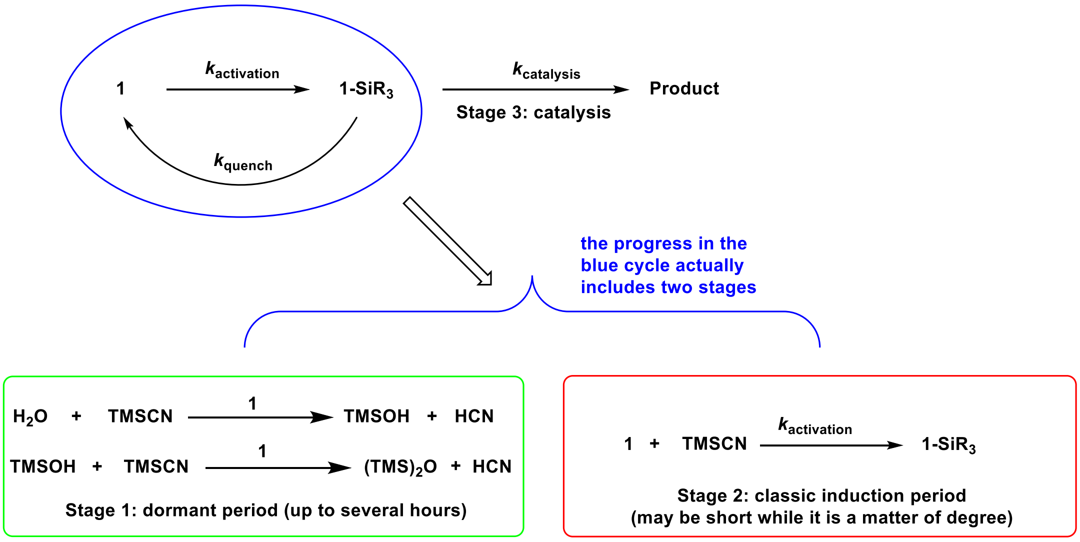

Stage 1 is the "dormant period", which is a very rare phenomenon in homogeneous catalysis. During **stage 1**, the desired reaction is completely dormant. **Stage 2** is the typical “induction period” where pre-catalyst **1** undergoes a transformation to form the active catalyst **1-SiR$_3$**. It is clear that **stage 1** and **stage 2** are different.

The cyanosilylation under study not only features a "dormant period", but also a short "induction period", which is between the point when the "dormant period" ends and the point when the desired reaction reaches the steady state (as shown in the following reaction profile monitored by *in situ* FT IR).

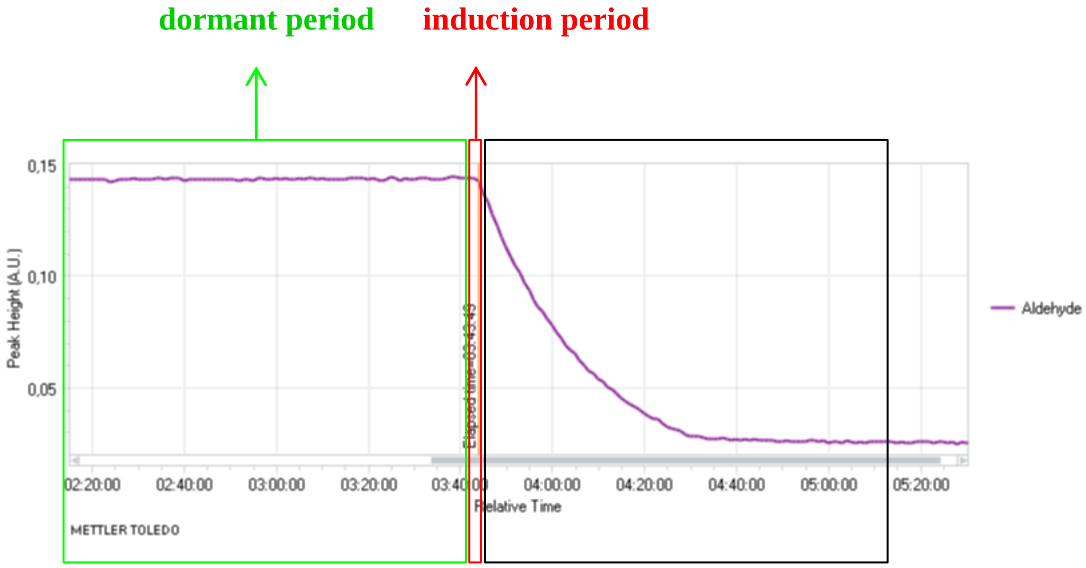

Supplementary Methods

General Information

Unless otherwise stated, all reagents were purchased from commercial suppliers and used without further purification. All solvents used in the reactions were distilled from appropriate drying agents prior to use. Liquid aldehydes were distilled and stored under argon. 2-naphthaldehyde was recrystallized from ethanol. Reactions were monitored by thin layer chromatography on silica gel or aluminum oxide pre-coated plastic sheets (0.2 mm, Macherey-Nagel). Visualization was accomplished by irradiation with UV light at 254 nm and/or PMA or $KMnO_4$ stain. Column chromatography was performed on silica gel (60, particle size 0.040-0.063 mm, Merck). Proton and carbon NMR spectra were recorded on a Bruker AV-500 or Bruker AV-400 spectrometer in deuterated solvent. ^{1}H chemical shifts are reported in ppm (δ) relative to tetramethylsilane with the solvent resonance employed as the internal standard (CD_2Cl_2, δ 5.32 ppm; $CDCl_3$, δ 7.26 ppm; DMSO-d6, δ 2.50 ppm). Data are reported as follows: chemical shift, multiplicity (s = singlet, d = doublet, t = triplet, q = quartet, m = multiplet, br = broad), coupling constants (Hz) and integration. ^{13}C chemical shifts are reported in ppm from tetramethylsilane with the solvent resonance as the internal standard (CD_2Cl_2, 53.8 ppm; $CDCl_3$, δ 77.0 ppm; DMSO-d6, δ 39.5 ppm). High resolution mass spectra were recorded on a Bruker APEX III FTMS (7 T magnet). The enantiomeric ratios were determined by HPLC analysis employing a chiral stationary phase column specified in the individual experiment, by comparing the samples with the appropriate racemic mixtures. Reaction progress profiles were recorded using a METTLER TOLEDO ReactIR® 15. The catalysts **1a-f** were synthesized according to the literature known procedures[4-8]. Abbreviations: e.r. (enantiomeric ratio), TLC (thin layer chromatography), THF (tetrahydrofuran), DME (1,2-dimethoxyethane).

General Procedure for the Asymmetric Cyanosilylation of Aldehydes Catalyzed by 1

Caution: TMSCN must be used carefully in a well-ventilated hood due to its high toxicity!

- General procedure for the asymmetric cyanosilylation of aldehydes catalyzed by **1**.

A dried vial with a Teflon-coated magnetic stirring bar was charged with disulfonimide **1** (0.0015 mmol, 3 mol%), aldehyde **2** (0.05 mmol, 1 equiv.) and Et_2O (0.31 mL). TMSCN (0.1 mmol, 2 equiv.) was added to the reaction mixture via a microliter syringe and the vial was cooled to –30 ℃ immediately. The reaction mixture was stirred at –30 ℃ and the

progress of the reaction was monitored by TLC. The reaction was quenched with 2% (v/v) trifluoroacetic acid (TFA) in CH_2Cl_2 (0.38 mL) and water (2 µL) after the progress of the reaction was determined to be complete. The reaction mixture was warmed up to room temperature and stirred for 2 hours to hydrolyze the TMS-protected cyanohydrin. The solvent and the volatile compounds were evaporated under reduced pressure at ambient temperature. Then CH_2Cl_2 (1 mL), acetic anhydride (47 µL, 10 equiv.) and pyridine (36 µL, 9 equiv.) were added and the reaction mixture was stirred at room temperature overnight. The solvent and volatile compounds were evaporated under reduced pressure at 40 °C. The residue was purified by column chromatography on silica gel using isohexane/ethyl acetate (4:1) as the eluent giving the respective cyanohydrin acetate in its pure form. The e.r. was determined by HPLC analysis of the cyanohydrin acetate using a chiral stationary phase.

- General procedure for the *in situ* study of the asymmetric cyanosilylation of aldehydes monitored by *in situ* FT-IR.

Disulfonimide **1a** (8.2 mg, 0.01 mmol) and Et_2O (4.54 mL) were placed in a pre-dried newly designed reactor (Supplementary Fig. 11) equipped with a Teflon-coated magnetic stirring bar and thermometer under argon. A probe rod of ReactIR 15 (METTLER TOLEDO) was inserted into the solution, the flask was connected to a high performance cryostat and the temperature was set to 20 °C. The scanning (interval: 1 min) was started and 2-naphthaldehyde (**2a**, 156.2 mg, 1.000 mmol) was added after a few minutes. The stretching vibration absorption of carbonyl group (1703 cm^{-1}) in **2a** was monitored. TMSCN (250 µL, 2.00 mmol) was added into the reaction mixture after 5 minutes and the reaction profile (IR vs time) was recorded. The reaction was quenched with water (10 µL) after most 2-naphthaldehyde (**2a**) had been converted to the product. The conversion of **2a** was determined by ^{1}H NMR.

Determination of Absolute Configuration

The absolute configuration was established by comparison of the optical rotation with the literature value for a sample of (*S*)-cyano(naphthalene-2-yl)methyl acetate **5a**:
Optical rotation: $[\alpha]_D^{25}$ = +23.9 (c = 0.50, $CHCl_3$); Lit: $[\alpha]_D^{25}$ = +20.9 (c = 1.13, $CHCl_3$) for a sample with 92.5:7.5 e.r. (*S*) [9].
All other compounds **5** were assigned by analogy.

Large-scale Reaction

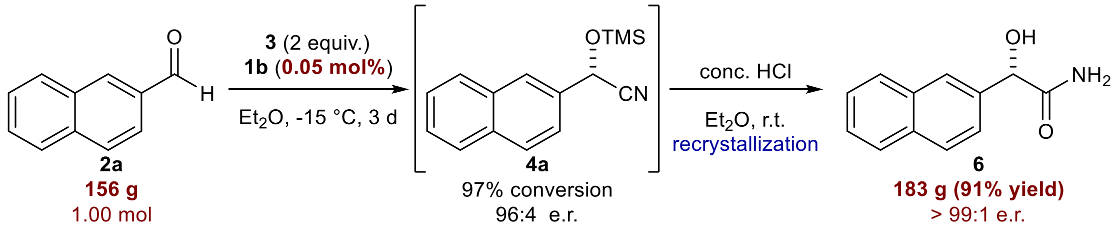

Caution: TMSCN must be used carefully in a well-ventilated hood due to its high toxicity!

Step 1: To a dried 4 L three-neck round-bottom flask with a Teflon-coated magnetic stirring bar, **2a** (156.2 g, 1.0 mol), **1b** (610 mg, 0.500 mmol, 0.0500 mol%) and Et_2O (2.5 L) were added. The solution was cooled to –15 ℃ and TMSCN (250 mL, 2.0 mol) was added. The reaction mixture was stirred at –15 ℃ for 3 d and a small portion of reaction mixture (0.3 mL) was sampled and converted to cyanohydrin acetate using the general procedure. The conversion was determined to be 97% by ^{1}H NMR spectroscopy and the e.r. was determined to be 96:4 by HPLC analysis. The reaction mixture was warmed to room temperature slowly and the Et_2O and all volatile compounds remaining in the flask were evaporated and condensed with a cold trap immersed in liquid N_2 under reduced pressure. A mixture of TMS-protected cyanohydrin **4a** and catalyst **1b** was obtained as light yellow solid.

Step 2: A mechanical stirrer was fixed on the flask and Et_2O (300 mL) was added. The resulting solution was cooled to 0 ℃. Conc. HCl (1.0 L) was added into the solution in one portion, the reaction mixture was warmed to room temperature slowly and stirred for 48 hours. The reaction mixture, which contains a large amount of solids, was then filtered and washed with Et_2O. A colorless solid was obtained and dried under high vacuum at 60 ℃. Product **6a** (183 g, 91%) was obtained as colorless powder. The e.r. of **6a** was determined to be higher than 99:1 by HPLC analysis. The filtrate was diluted with water (1.0 L) and extracted with Et_2O (3×300 mL). The combined organic layers, which contain catalyst **1b** were dried over $MgSO_4$ and evaporated. The catalyst (510 mg, approx. 80%) was recovered as salt after purification of the residue by column chromatography on silica gel using isohexane/ethyl acetate (5:1 to 2:1) as eluent.

Reaction at Low Catalyst Loading

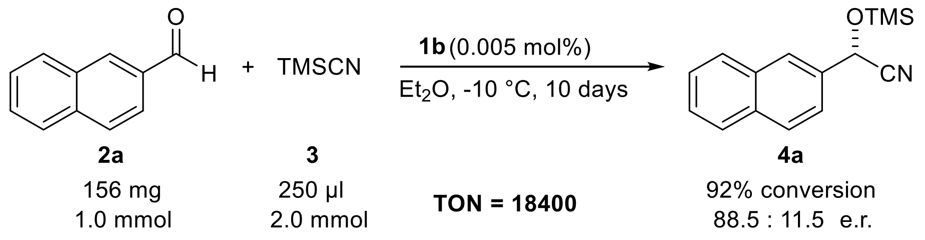

To a dried vial equipped with a Teflon-coated magnetic stirring bar, aldehyde **2a** (156 mg, 1.0 mmol) and Et_2O (2.0 mL) were added. Disulfonimide **1b** (1.0 mM solution in Et_2O, 50 µL, 0.00005 mmol, 0.005 mol%) and TMSCN (250 µL, 2.0 mmol) were added to the reaction mixture via a microliter syringe and the vial was cooled to –10 °C immediately. The reaction mixture was stirred at –10 °C for 10 d, then quenched and converted to cyanohydrin acetate as described in the general procedure. The conversion was determined to be 92% by ^{1}H NMR and the e.r. was determined to be 88.5:11.5 by HPLC analysis using a chiral stationary phase. The TON was calculated to be 18400.

NMR Study on the Silylated Catalyst 1a

The silylation of disulfonimides can lead to two different species: *N*- and *O*-silylated species[1,2]. Both species have been characterized in solution by NMR spectroscopy for flexible disulfonimides. In order to understand the silylation behavior of our non-flexible, bulky disulfonimide catalyst **1**, NMR measurements on the silylated catalyst **12a** were conducted after silylation of **1a** with two different silyl ketene acetals (**7a** and **7b**). The formed silylated catalysts **12a** were characterized using ^{1}H-^{29}Si-HMBC (Supplementary Fig. 8) and ^{1}H NMR experiments. The results obtained were compared to the literature values and are shown in Supplementary Table 2 [1,2].

The ratio between **12a-*O*-TMS** and **12a-*N*-TMS** was determined to be 3:1 based on the ^{1}H NMR signals of the BINOL-backbone. In contrast to this, the ratio between **12a-*O*-TBS** and **12a-*N*-TBS** was determined to be 10:1. The proton signal of the TMS group in **12a-*O*-TMS** is broad, which could be due to a fast exchange of the TMS group in the two possible diasteromeric structures of **12a-*O*-TMS**. Using 2D-EXSY experiments, we were also able to observe the exchange between the *N*- and *O*-silylated species (Supplementary Fig. 9).

Characterization of Products 5a-p and 6

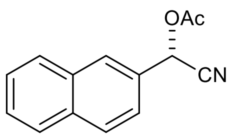

5a

(S)-cyano(naphthalene-2-yl)methyl acetate **5a**. Colorless solid, 11.0 mg. Yield: 98%. mp: 44–45 ℃. ^{1}H NMR (500 MHz, $CDCl_3$): δ 2.19 (s, 3H), 6.59 (s, 1H), 7.56–7.58 (m, 3H), 7.87–7.94 (m, 3H), 8.02 (s, 1H). ^{13}C NMR (125 MHz, $CDCl_3$): δ 20.50, 63.04, 116.13, 124.26, 127.07, 127.57, 127.81, 128.01, 128.36, 128.88, 129.43, 132.82, 133.85, 168.97. IR: 1748, 1370, 1210, 1019, 981, 943, 859, 817, 752 cm^{-1}. MS (EI) *m/z*: 43 (18%), 165 (99%), 166 (66%), 183 (100%), 225 (47%). HRMS (EI) *m/z*: calcd. for $C_{14}H_{11}NO_2$, 225.0788, found 225.0790.

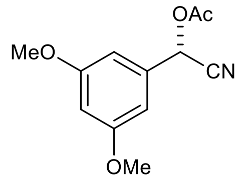

5b

(S)-cyano(3,5-dimethoxyphenyl)methyl acetate **5b**. Colorless liquid, 11.4 mg. Yield: 97%. ^{1}H NMR (500 MHz, $CDCl_3$): δ 2.18 (s, 3H), 3.81 (s, 6H), 6.33 (s, 1H), 6.51 (s, 1H), 6.63 (s, 2H). ^{13}C NMR (125 MHz, $CDCl_3$): δ 20.44, 55.51, 62.68, 102.08, 105.69, 115.98, 133.56, 161.27, 168.84. IR: 2919, 1751, 1597, 1460, 1204, 1157, 1022, 838 cm^{-1}. MS (EI) *m/z*: 43 (12%), 176 (15%), 193 (100%), 235 (31%). HRMS (ESI+) *m/z*: $[M+Na]^+$ calcd. for $C_{12}H_{13}NO_4Na$, 258.0735, found 258.0737.

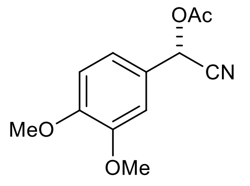

5c

(S)-cyano(3,4-dimethoxyphenyl)methyl acetate **5c**. Colorless solid, 11.1 mg. Yield: 94%. mp: 87–88 ℃. ^{1}H NMR (500 MHz, $CDCl_3$): δ 2.15 (s, 3H), 3.90 (s, 3H), 3.92 (s, 3H), 6.35 (s, 1H), 6.89 (d, J = 8.3 Hz, 1H), 6.99 (d, J = 2.1 Hz, 1H), 7.09 (dd, J = 8.3, 2.1 Hz, 1H). ^{13}C NMR (125 MHz, $CDCl_3$): δ 20.51, 55.97, 56.00, 62.80, 110.64, 111.08, 116.26, 121.14, 123.98, 149.43, 150.68, 168.98. IR: 2940, 1747, 1592, 1521, 1210, 1142, 1008, 825 cm^{-1}. MS (EI) *m/z*: 43 (18%), 160 (19%), 176 (100%), 193 (55%), 235 (51%). HRMS (ESI+) *m/z*: $[M+Na]^+$ calcd. for $C_{12}H_{13}NO_4Na$, 258.0738, found 258.0737.

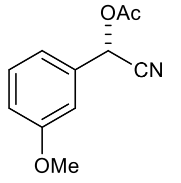

5d

(S)-cyano(3-methoxyphenyl)methyl acetate **5d**. Colorless liquid, 9.9 mg. Yield: 96%. ^{1}H NMR (500 MHz, $CDCl_3$): δ 2.17 (s, 3H), 3.84 (s, 3H), 6.38 (s, 1H), 6.99 (dd, J = 8.4, 2.3 Hz, 1H), 7.03 (t, J = 2.0 Hz, 1H), 7.09 (d, J = 7.7 Hz, 1H), 7.36 (t, J = 8.0 Hz, 1H). ^{13}C NMR (125 MHz, $CDCl_3$): δ 20.44, 55.39, 62.67, 113.22, 115.99, 116.04, 119.93, 130.32, 132.97, 160.06, 168.87. IR: 2919, 1750, 1603, 1491, 1371, 1208, 1020, 789, 693 cm^{-1}. MS (EI) *m/z*:

43 (25%), 146 (26%), 163 (100%), 205 (20%). HRMS (EI) *m/z*: calcd. for $C_{11}H_{11}NO_3$, 205.0737, found 205.0739.

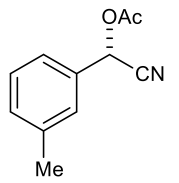

5e

(*S*)-cyano(*m*-tolyl)methyl acetate **5e**. Colorless liquid, 8.5 mg. Yield: 90%. ^{1}H NMR (500 MHz, $CDCl_3$): δ 2.16 (s, 3H), 2.40 (s, 3H), 6.37 (s, 1H), 7.26–7.27 (m, 1H), 7.30–7.35 (m, 3H). ^{13}C NMR (125 MHz, $CDCl_3$): δ 20.45, 21.27, 62.82, 116.17, 124.91, 128.43, 129.07, 131.12, 131.56, 139.19, 168.91. IR: 1751, 1370, 1208, 1020, 789, 698 cm^{-1}. MS (ESI+) *m/z*: 212 $[M+Na]^+$. HRMS (EI) *m/z*: calcd. for $C_{11}H_{11}NO_2$, 189.0791, found 189.0790.

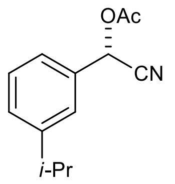

5f

(*S*)-cyano(3-isopropylphenyl)methyl acetate **5f**. Colorless liquid, 10.5 mg. Yield: 97%. ^{1}H NMR (500 MHz, $CDCl_3$): δ 1.27 (d, *J* = 6.9 Hz, 6H), 2.17 (s, 3H), 2.96 (hept, *J* = 6.9 Hz, 1H), 6.40 (s, 1H), 7.32–7.38 (m, 4H). ^{13}C NMR (125 MHz, $CDCl_3$): δ 20.50, 23.79, 23.84, 62.96, 116.22, 125.36, 126.03, 128.53, 129.22, 131.57, 150.18, 168.96. IR: 2963, 1751, 1370, 1208, 1020, 796, 702 cm^{-1}. MS (ESI+) *m/z*: 240 $[M+Na]^+$. HRMS (ESI+) *m/z*: $[M+Na]^+$ calcd. for $C_{13}H_{15}NO_2Na$, 240.0995, found 240.0995.

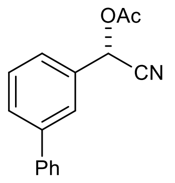

5g

(*S*)-[1,1'-biphenyl]-3-yl(cyano)methyl acetate **5g**. Colorless liquid, 11.8 mg. Yield: 94%. ^{1}H NMR (500 MHz, $CDCl_3$): δ 2.17 (s, 3H), 6.47 (s, 1H), 7.39 (tt, *J* = 7.5, 1.5 Hz, 1H), 7.45–7.54 (m, 4H), 7.58–7.59 (m, 2H), 7.67 (td, *J* = 7.5, 1.5 Hz, 1H), 7.72 (s, 1H). ^{13}C NMR (125 MHz, $CDCl_3$): δ 20.45, 62.83, 116.09, 126.58, 126.60, 127.15, 127.91, 128.92, 129.11, 129.66, 132.22, 139.80, 142.43, 168.90. IR: 1750, 1480, 1370, 1209, 1021, 758, 699 cm^{-1}. MS (ESI+) *m/z*: 274 $[M+Na]^+$. HRMS (ESI+) *m/z*: $[M+Na]^+$ calcd. for $C_{16}H_{13}NO_2Na$, 274.0837, found 274.0838.

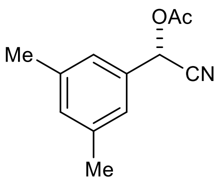

5h

(*S*)-cyano(3,5-dimethylphenyl)methyl acetate **5h**. Colorless liquid, 9.7 mg. Yield: 95%. ^{1}H NMR (500 MHz, $CDCl_3$): δ 2.17 (s, 3H), 2.35 (s, 6H), 6.34 (s, 1H), 7.09 (s, 1H), 7.12 (s, 2H). ^{13}C NMR (125 MHz, $CDCl_3$): δ 20.50, 21.17, 62.86, 116.28, 125.59, 131.47, 132.02, 139.06, 168.96. IR: 2923, 1752, 1611, 1370, 1207, 1017, 846, 694 cm^{-1}. MS (ESI+) *m/z*: 226 $[M+Na]^+$. HRMS (ESI+) *m/z*: $[M+Na]^+$ calcd. for $C_{12}H_{13}NO_2Na$, 226.0839, found 226.0838.

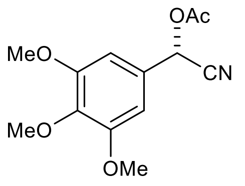

5i

(*S*)-cyano(3,4,5-trimethoxyphenyl)methyl acetate **5i**. Colorless liquid, 12.9 mg. Yield: 97%. ^{1}H NMR (500 MHz, $CDCl_3$): δ 2.17 (s, 3H), 3.85 (s, 3H), 3.89 (s, 6H), 6.33 (s, 1H), 6.71 (s, 2H). ^{13}C NMR (125 MHz, $CDCl_3$): δ 20.49, 56.23, 60.83, 62.95, 105.11, 116.07, 126.94, 139.54, 153.68, 168.89. IR: 2941, 1751, 1594, 1462, 1208, 1124, 1000, 827 cm^{-1}. MS (EI) *m/z*: 43 (23%), 190 (30%), 206 (99%), 223 (100%), 265 (59%). HRMS (ESI+) *m/z*: $[M+Na]^+$ calcd. for $C_{13}H_{15}NO_5Na$, 288.0843, found 288.0842.

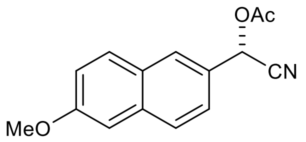

5j

(*S*)-cyano(6-methoxynaphthalen-2-yl)methyl acetate **5j**. Colorless solid, 12.6 mg. Yield: 99%. mp: 113–114 ℃. ^{1}H NMR (500 MHz, $CDCl_3$): δ 2.18 (s, 3H), 3.94 (s, 3H), 6.55 (s, 1H), 7.16 (d, *J* = 2.0 Hz, 1H), 7.22 (dd, *J* = 9.0, 2.5 Hz, 1H), 7.53 (dd, *J* = 8.5, 1.5 Hz, 1H), 7.78 (d, *J* = 9.0 Hz, 1H), 7.81 (d, *J* = 8.5 Hz, 1H), 7.94 (d, *J* = 1.5 Hz, 1H). ^{13}C NMR (125 MHz, $CDCl_3$): δ 20.53, 55.37, 63.10, 105.67, 116.27, 119.99, 124.96, 126.56, 127.90, 128.11, 128.24, 129.84, 135.33, 158.90, 169.03. IR: 2964, 1759, 1609, 1486, 1265, 1203, 983, 854 cm^{-1}. MS (ESI+) *m/z*: 278 $[M+Na]^+$. HRMS (ESI+) *m/z*: $[M+Na]^+$ calcd. for $C_{15}H_{13}NO_3Na$, 278.0785, found 278.0788.

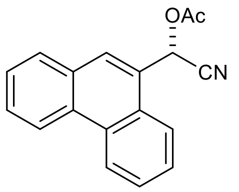

5k

(*S*)-cyano(phenanthren-9-yl)methyl acetate **5k**. Colorless solid, 13.6 mg. Yield: 99%. mp: 132–133 ℃. ^{1}H NMR (500 MHz, $CDCl_3$): δ 2.22 (s, 3H), 7.08 (s, 1H), 7.65–7.77 (m, 4H), 7.97 (d, *J* = 7.8 Hz, 1H), 8.04 (d, *J* = 7.8 Hz, 1H), 8.12 (s, 1H), 8.70 (d, *J* = 8.4 Hz, 1H), 8.78 (d, *J* = 7.9 Hz, 1H). ^{13}C NMR (125 MHz, $CDCl_3$): δ 20.51, 61.74, 116.07, 122.64, 123.42, 123.62, 125.43, 127.37, 127.53, 128.21, 128.50, 129.41, 129.65, 130.31, 131.04, 131.27, 169.11. IR: 1743, 1367, 1211, 987, 885, 747, 725 cm^{-1}. MS (ESI+) *m/z*: 298 $[M+Na]^+$. HRMS (ESI+) *m/z*: $[M+Na]^+$ calcd. for $C_{18}H_{13}NO_2Na$, 298.0839, found 298.0839.

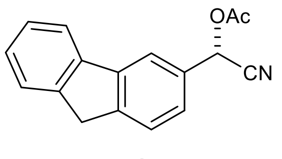

5l

(*S*)-cyano(9H-fluoren-3-yl)methyl acetate **5l**. Colorless solid, 13.1 mg. Yield: 99%. mp: 119–120 ℃. ^{1}H NMR (500 MHz, $CDCl_3$): δ 2.19 (s, 3H), 3.94 (s, 2H), 6.49 (s, 1H), 7.37 (dt, *J* = 7.4, 1.2 Hz, 1H), 7.41 (t, *J* = 7.5 Hz, 1H), 7.53 (d, *J* = 7.9 Hz, 1H), 7.58 (d, *J* = 7.4 Hz, 1H), 7.71 (s, 1H), 7.82 (t, *J* = 7.5 Hz, 2H). ^{13}C NMR (125 MHz, $CDCl_3$): δ 20.54, 36.82, 63.12, 116.33, 120.38, 120.42, 124.71, 125.16, 126.88, 126.99,

127.66, 129.78, 140.39, 143.59, 143.97, 144.12, 169.00. IR: 1745, 1369, 1204, 1016, 975, 837, 770, 739 cm^{-1}. MS (ESI+) *m/z*: 286 [M+Na]$^+$. HRMS (ESI+) *m/z*: [M+Na]$^+$ calcd. for $C_{17}H_{13}NO_2Na$, 286.0836, found 286.0838.

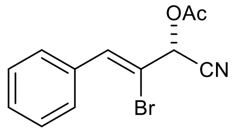

5m

(*R*, *Z*)-2-bromo-1-cyano-3-phenylallyl acetate **5m**. Colorless liquid, 11.5 mg. Yield: 82%. ^{1}H NMR (500 MHz, $CDCl_3$): δ 2.24 (s, 3H), 6.23 (d, *J* = 0.5 Hz, 1H), 7.37–7.43 (m, 4H), 7.66–7.68 (m, 2H). ^{13}C NMR (125 MHz, $CDCl_3$): δ 20.38, 66.54, 113.53, 114.40, 128.44, 129.28, 129.63, 133.19, 135.37, 168.45. IR: 1754, 1694, 1602, 1370, 1201, 1021, 754, 690 cm^{-1}. MS (ESI+) *m/z*: 302 [M+Na]$^+$. HRMS (ESI+) *m/z*: [M+Na]$^+$ calcd. for $C_{12}H_{10}NO_2BrNa$, 301.9785, found 301.9787.

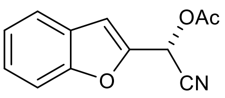

5n

(*R*)-benzofuran-2-yl(cyano)methyl acetate **5n**. Colorless liquid, 10.4 mg. Yield: 97%. ^{1}H NMR (400 MHz, $CDCl_3$): δ 2.21 (s, 3H), 6.63 (s, 1H), 7.07 (s, 1H), 7.30 (dd, *J* = 7.2, 6.8 Hz, 1H), 7.40 (dd, *J* = 7.2, 7.2 Hz, 1H), 7.53 (d, *J* = 8.4 Hz, 1H), 7.63 (d, *J* = 7.6 Hz, 1H). ^{13}C NMR (100 MHz, $CDCl_3$): δ 20.28, 56.30, 108.99, 111.79, 113.87, 122.00, 123.70, 126.27, 126.89, 146.19, 155.61, 168.61. IR: 1752, 1452, 1370, 1202, 1019, 819, 749 cm^{-1}. MS (EI) *m/z*: 43 (18%), 127 (23%), 155 (90%), 156 (100%), 173 (55%), 215 (54%). HRMS (EI) *m/z*: calcd. for $C_{12}H_9NO_3$, 215.0580, found 215.0582.

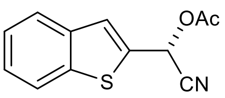

5o

(*R*)-benzo[*b*]thiophen-2-yl(cyano)methyl acetate **5o**. Colorless liquid, 11.3 mg. Yield: 98%. ^{1}H NMR (400 MHz, $CDCl_3$): δ 2.20 (s, 3H), 6.72 (s, 1H), 7.40–7.44 (m, 2H), 7.60 (s, 1H), 7.81–7.85 (m, 2H). ^{13}C NMR (100 MHz, $CDCl_3$): δ 20.34, 58.80, 115.04, 122.48, 124.59, 125.07, 125.98, 126.28, 133.80, 138.27, 140.59, 168.68. IR: 2961, 1750, 1370, 1258, 1204, 1014, 791 cm^{-1}. MS (EI) *m/z*: 43 (13%), 145 (14%), 171 (100%), 172 (78%), 189 (63%), 231 (48%). HRMS (EI) *m/z*: calcd. for $C_{12}H_9NO_2S$, 231.0352, found 231.0354.

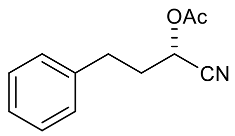

5p

(*S*)-1-cyano-3-phenylpropyl acetate **5p**. Colorless liquid, 4.6 mg. Yield: 45%. ^{1}H NMR (500 MHz, $CDCl_3$): δ 2.13 (s, 3H), 2.24 (td, *J* = 7.5, 7.0 Hz, 2H), 2.84 (t, *J* = 7.5 Hz, 2H), 5.26 (t, *J* = 7.0 Hz, 1H), 7.18 (d, *J* = 7.5 Hz, 2H), 7.24 (t, *J* = 7.5 Hz, 1H), 7.31 (t, *J* = 7.5 Hz, 2H). ^{13}C NMR (125 MHz, $CDCl_3$): δ 20.32, 30.70, 33.74, 60.48, 116.69, 126.70, 128.30,

128.77, 138.97, 169.07. IR: 1750, 1604, 1497, 1455, 1372, 1213, 1038, 747, 699 cm^{-1}. MS (ESI+) *m/z*: 226 $[M+Na]^+$. HRMS (ESI+) *m/z*: $[M+Na]^+$ calcd. for $C_{12}H_{13}NO_2Na$, 226.0837, found 226.0838.

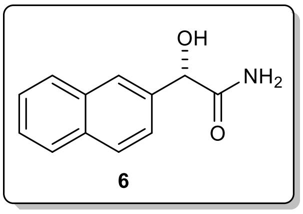

(*S*)-2-hydroxy-2-(naphthalen-2-yl)acetamide **6**. Colorless powder, 183 g. Yield: 91%. $[\alpha]_D^{25}$ = +60.8 (*c* = 0.50, DMSO) {Lit.[10] $[\alpha]_D^{22}$ = -62.8 (*c* = 0.5, DMSO) for (*R*)-2-hydroxy-2-(naphthalen-2-yl)acetamide}. ^{1}H NMR (500 MHz, DMSO-d6): δ 5.03 (s, 1H), 6.10 (br, 1H), 7.25 (s, 1H), 7.47–7.53 (m, 3H), 7.59 (dd, *J* = 8.5, 1.6 Hz, 1H), 7.86–7.93 (m, 4H). ^{13}C NMR (125 MHz, DMSO-d6): δ 73.57, 124.84, 125.12, 125.79, 126.08, 127.38, 127.48, 127.74, 132.41, 132.60, 138.96, 174.45. MS (ESI+) *m/z*: 224 $[M+Na]^+$. HRMS (ESI+) *m/z*: $[M+Na]^+$ calcd. for $C_{12}H_{11}NO_2Na$, 224.0682, found 224.0683.

Supplementary References

1. Blaschette, A., Wieland, E., Hamann, T. & Harris, R. K. Trimethylsilyl-dimesylamin: Darstellung, NMR-spektroskospische Charakterisierung und Reaktionsfaehigkeit als Silylierungsreagens. *Z. Naturforsch.* **47b**, 1693–1700 (1992).
2. Simchen, G. & Jonas, S. Hochreaktive Trialkylsilylierungsreagentien aus Bis(trifluormethansulfony1)imid-Silylierung von funktionellen Gruppen, Alkinen und reaktiven Aromaten). *J. Prakt. Chem.* **340**, 506–512 (1998).
3. McNaught, A. D. & Wilkinson, A. *IUPAC. Compendium of Chemical Terminology, 2nd ed. (the "Gold Book")* (Blackwell Scientific Publications, Oxford, 1997).
4. Garc á-Garc á, P., Lay, F., Garc á-Garc á, P., Rabalakos, C. & List, B. A powerful chiral counteranion motif for asymmetric catalysis. *Angew. Chem. Int. Ed.* **48,** 4363–4366 (2009).
5. Mahlau, M., Garc á-Garc á, P. & List, B. Asymmetric counteranion-directed catalytic Hosomi–Sakurai reaction. *Chem. Eur. J.* **18,** 16283–16287 (2012).
6. Wang, Q., Leutzsch, M., van Gemmeren, M. & List, B. Disulfonimide-catalyzed asymmetric synthesis of β^3-amino esters directly from *N*-Boc-Amino sulfones. *J. Am. Chem. Soc.* **135,** 15334–15337 (2013).

7. Gandhi, S. & List, B. Catalytic asymmetric three-component synthesis of homoallylic amines. *Angew. Chem. Int. Ed.* **52,** 2573–2576 (2013).
8. Guin, J., Rabalakos, C. & List, B. Highly enantioselective hetero-Diels–Alder reaction of 1,3-bis-(silyloxy)-1,3-dienes with aldehydes catalyzed by chiral disulfonimide. *Angew. Chem. Int. Ed*. **51,** 8859–8863 (2012).
9. Inagaki, M., Hiratake, J., Nishioka, T. & Oda, J. Lipase-catalyzed kinetic resolution with in situ racemization: one-pot synthesis of optically active cyanohydrin acetates from aldehydes. *J. Am. Chem. Soc.* **113,** 9360–9361 (1991).
10. Kimura, M., Kuboki, A. & Sugai, T. Chemo-enzymatic synthesis of enantiomerically pure (*R*)-2-naphthylmethoxyacetic acid. *Tetrahedron: Asymmetry* **13,** 1059–1068 (2002).
